# Transsulfuration metabolism is essential for ferroptosis resistance in quiescent endothelial cells

**DOI:** 10.1038/s41419-025-08333-1

**Published:** 2025-12-20

**Authors:** Roxana Elena Oberkersch, Jacopo Lidonnici, Sebastiano Andreuzza, Eleonora Zambon, Gabriele Imperato, Silvia Pedretti, Nico Mitro, Massimo Mattia Santoro

**Affiliations:** 1 Laboratory of Angiogenesis and Cancer Metabolism, Department of Biology, University of Padua, Padua, Italy; 2 DiSFeB, Department of Pharmacological and Biomolecular Sciences “Rodolfo Paoletti”, University of Milan, Milan, Italy; 3 Department of Experimental Oncology, IEO, European Institute of Oncology IRCCS, Milan, Italy

**Keywords:** Metabolic pathways, Cell signalling, Cardiovascular diseases

## Abstract

Angiogenesis, the formation of new blood vessels from pre-existing ones, is a crucial process involved in both physiological and pathological contexts. During angiogenesis, quiescent endothelial cells (QECs) forming the vascular bed begin to proliferate and switch their metabolism to support anabolic and energetic needs in response to growth factors and hypoxic conditions. Recent research has demonstrated that ferroptosis, an iron-dependent form of cell death mediated by lipid peroxidation, can affect angiogenesis. Cysteine, a thiol-containing amino acid, is crucial for the synthesis of sulfur-containing biomolecules that control ferroptosis. Glutathione (GSH), a reducing tripeptide containing a cysteine residue, serves as a cofactor for the enzyme glutathione peroxidase 4 (GPX4) to donate electrons to peroxides of polyunsaturated fatty acyl phospholipids. Cysteine can be acquired from its extracellular oxidized form, cystine, via the glutamate-cystine antiporter (system xCT) or synthesized de novo via the transsulfuration pathway (TSP). However, whether proliferating ECs (PECs) and QECs differentially modulate the cysteine/GSH/GPX4 axis to protect themselves from ferroptosis is still unknown. Our findings revealed that PECs primarily utilize extracellular cystine to synthesize GSH, which is essential for avoiding ferroptosis. In contrast, QECs exhibit a resilient response to cystine starvation by activating the TSP. Interestingly, chronic and severe hypoxia induces ferroptosis resistance in PECs exposed to cystine limitation, mimicking the metabolic profile of QECs. Molecularly, QECs exhibit high NRF2 expression necessary to support TSP under cystine limitation and protect QECs from ferroptosis. In vivo experiments confirm the susceptibility of ECs to cell death by xCT inhibition in a retinal model of sprouting angiogenesis. These findings highlight differential regulation of cysteine metabolism in PECs and QECs and suggest that the cysteine/GSH/GPX4 axis could be a potential therapeutic target for diseases involving angiogenesis.

## Introduction

The human body houses an intricate network of blood vessels, which are lined by a monolayer of endothelial cells (ECs). This network undergoes a significant expansion during development, wound healing, and specific pathological conditions, such as tumor growth. In adult mammals, most vessel growth is mediated by the expansion of pre-existing vessel beds, a process known as angiogenesis [1]. During angiogenesis, specific stimuli reactivate quiescent endothelial cells (QECs) that are characterized by reversible proliferative arrest. As a result, these cells rapidly re-enter the cell cycle to proliferate [2]. Despite the remarkable diversity, QECs must be able to persist in a non-dividing state over extended periods and enact mechanisms to protect themselves from damage [3]. Thus, QECs extensively remodel their gene expression programs [4–6] and metabolism [7–9] to induce a common dormant and protective cellular state. QECs up-regulate fatty acid oxidation (FAO) to sustain the tricarboxylic acid (TCA) cycle to maintain redox homeostasis through NADPH regeneration [10]. Additionally, QECs increase the catabolic intermediates of branched-chain amino acids (BCAAs) in a FOXO1-dependent manner to promote quiescence [11]. Although these studies show metabolic adaptations in QECs affecting nutrient exchange, redox metabolism, and ATP production, it remains unclear whether metabolic adaptations are needed to protect QECs from adverse environmental conditions, such as increased oxidative stress, metabolite availability, and iron homeostasis occurring in the vascular bed.

Ferroptosis is a unique type of non-apoptotic-regulated cell death culminating with damaging lipid peroxidation downstream of metabolic dysfunctions [12, 13]. Cysteine (Cys) deprivation represents a potent inducer of ferroptosis due to its pivotal role in glutathione (GSH) formation. Cysteine is a nonessential sulfur-containing proteinogenic amino acid whose intracellular levels are predominantly maintained by the cystine (Cys-Cys)/glutamate antiporter xCT (encoded by the *SLC7A11* gene). Despite its extracellular abundance (around 50 μM), Cys-Cys availability may become limiting for rapidly growing tissues [14]. For example, in tumorigenic settings, high Cys-Cys consumption can make the tumor microenvironment Cys-Cys-poor [15]. However, it has been reported that tumor cells can survive extracellular Cys-Cys deprivation through the activity of the transsulfuration pathway (TSP) [16] and that targeting TSP enzymes reduces tumor volume and angiogenesis markers in mouse xenograft model [17].

TSP is a specific metabolic pathway that allows the de novo synthesis of Cys from methionine (Met) in a two-step enzymatic reaction. During the first reaction, cystathionine β-synthase (CBS) condenses homocysteine (Hcy) produced by the methionine cycle and a serine molecule into cystathionine, while in the second step, cystathionine γ-lyase (CTH) hydrolyses cystathionine into α-ketobutyrate and Cys. CBS is a pyridoxal 5′-phosphate (PLP)-dependent heme protein constitutively expressed [18], whereas CTH is inducible by a wide range of stimuli such as nutrient deprivation or oxidative stress. In fact, in ECs, a deficiency of sulfur amino acids can induce the upregulation of CTH through the GCN2/ATF4 axis and trigger angiogenesis [19]. Conversely, the chemical inhibition of xCT through sublethal concentrations of erastin can lead to ECs activation through a ferroptosis-like mechanism [20].

Although nearly all ECs in an adult individual are in a quiescent state, most studies focus on PECs because of their role in driving vessel sprouting. Actively growing ECs are sensitive to ferroptotic cell death [21]; however, to our knowledge, no study has investigated whether quiescent ECs respond in the same way as proliferating ECs to ferroptosis-promoting stimuli. Therefore, it remains unclear whether QECs exhibit a Cys-dependent metabolic ferroptosis-protective mechanism that is differentially modulated in PECs, potentially opening a novel metabolically targetable window in the angiogenic field.

In the present study, we show that QECs exhibit greater resistance to ferroptosis under Cys-Cys restriction compared to angiogenic PECs, and impairment of the TSP leads QECs to become sensitive to ferroptosis triggered by Cys-Cys deficiency. This resistance is dependent on NRF2, which is essential for the induction of TSP enzymes during Cys-Cys limitation. Additionally, hypoxia drives metabolic reprogramming of the TSP, enhancing ferroptosis resistance in PECs.

## Results

### Proliferating ECs require exogenous cystine to avert ferroptosis

**Fig. 1:**
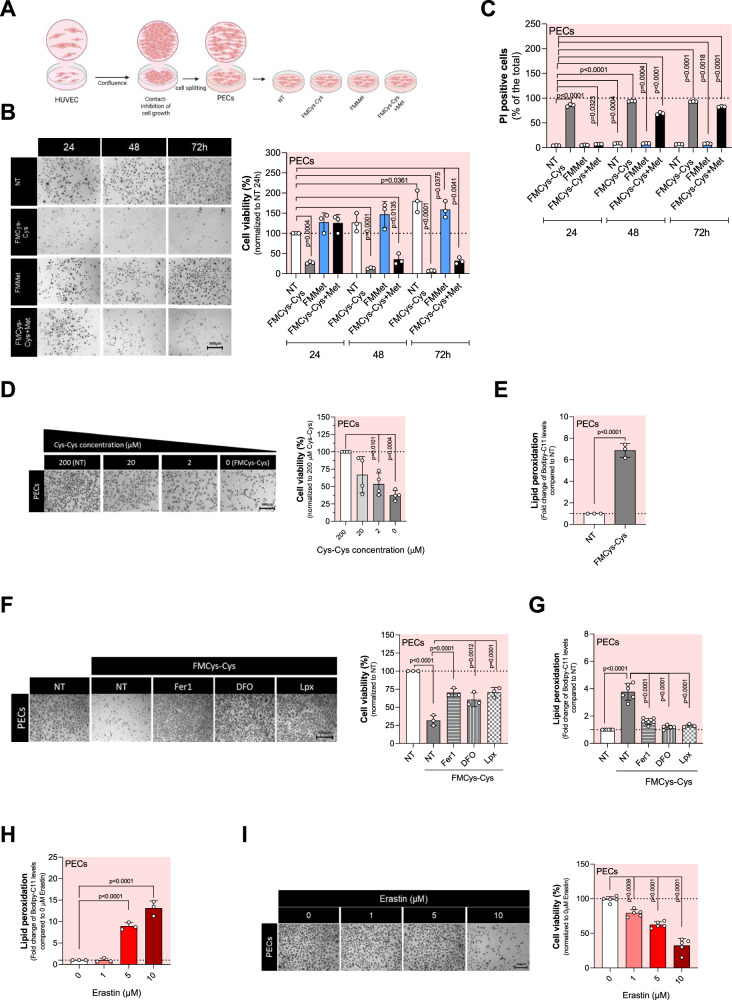

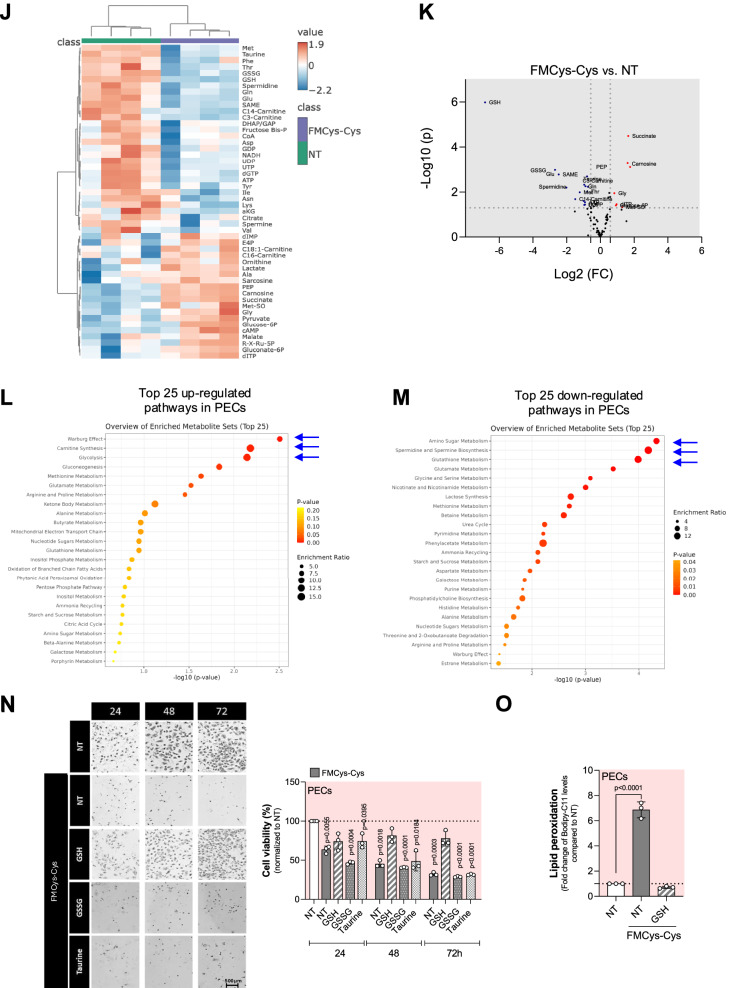
Cystine deprivation induces ferroptosis in proliferating ECs. **A** Diagram explaining the experimental protocol used to assess cellular responses under various metabolic conditions of PECs. **B** Cell viability assay using crystal violet staining on proliferating HUVECs (PECs) grown in cystine-(FMCys-Cys), methionine- (FMMet) or both-(FMCys-Cys+Met) free medium for 24, 48 and 72 h. At least 3 biological replicates were quantified and the percentage of living cells was calculated using cells grown in 200 μM cystine and methionine 100 µM as a reference (untreated, NT). Statistical significance is given by one sample t-test and reported as a p-value in the figure. **C** Cell death analysis using PI staining on proliferating HUVECs (PECs) grown in cystine-(FMCys-Cys), methionine- (FMMet) or both-(FMCys-Cys+Met) free medium for 24, 48 and 72 h. Three biological replicates were quantified and the percentage of living cells was calculated using cells grown in 200 μM cystine as a reference. Statistical significance is given by one sample t-test and reported as a p-value in the figure. **D** Cell viability assay using crystal violet staining on proliferating HUVECs (PECs) grown in different cystine concentration (0; 2; 20; 200 μM) for 24 h. At least 3 biological replicates were quantified and the percentage of living cells was calculated using cells grown in 200 μM cystine as a reference. Statistical significance is given by one sample t-test and reported as a p-value in the figure. **E** Lipid peroxidation levels measured by Bodipy-C11 FACS in PECs grown in 200 μM (NT) and 0 µM cystine (FMCys-Cys) for 24 h. The ratio between oxidized and reduced Bodipy-C11 was measured and reported as fold change over control (NT). Statistical significance is given by one sample t-test and reported as a p-value in the figure. **F** Cell viability assay using crystal violet staining on PECs grown in FMCys-Cys and supplemented with 10 μM Ferrostatin-1 (Fer-1), 100 μM Deferoxamine (DFO) or 2 μM Liprosxtatin-1 (Lpx). At least 3 biological replicates were quantified and the percentage of living cells was calculated using cells grown in 200 μM cystine as a reference. Statistical significance is given by one sample t-test and reported as a p-value in the figure. **G** Lipid peroxidation levels measured by Bodipy-C11 581/591 FACS in PECs grown in 200 μM cystine medium (NT), or in FMCys-Cys and supplemented with 10 μM Fer-1, 100 μM DFO or 2 μM Lpx. Statistical significance is given by one sample t-test and reported as adjusted p-value in the figure. **H** Lipid peroxidation levels measured by Bodipy-C11 FACS in PECs treated with different concentration (0; 1; 5; 10 μM) of erastin for 24 h. Statistical significance is given by one sample t-test and reported as adjusted p-value in the figure. **I** Cell viability assay using crystal violet staining on PECs treated with different concentration (0; 1; 5; 10 μM) of erastin for 24 h. At least 3 biological replicates were quantified and the percentage of living cells was calculated using untreated (NT) condition as a reference. Statistical significance is given by one sample t-test and reported as a p-value in the figure. **J** Heat-map of top 50 polar metabolites differentially present in PECs grown in 200 μM cystine (green) or FMCys-Cys (blue) medium for 24 h. **K** Volcano plot of significantly up- and down-regulate metabolites in PECs grown in 200 μM cystine or FMCys-Cys medium for 24 h. **L** Metabolome enrichment analysis of top 25 up-regulated pathways in PECs grown in 200 μM cystine or FMCys-Cys medium for 24 h. **M** Metabolome enrichment analysis of top 25 down-regulated pathways in PECs grown in 200 μM cystine or FMCys-Cys medium for 24 h. **N** Cell viability assay using crystal violet staining on PECs grown over three days in FMCys-Cys and supplemented with 10 mM GSH, 10 mM GSSG or 1 mM taurine. At least 3 biological replicates were quantified and the percentage of living cells was calculated using cells grown in 200 μM cystine (NT) as a reference. Statistical significance is given by one sample t-test and reported as a p-value in the figure. **O** Lipid peroxidation levels measured by Bodipy-C11 FACS in PECs grown in FMCys-Cys medium supplemented with 10 mM GSH for 24 h. Statistical significance is given by one sample t-test and reported as adjusted p-value in the figure.

To address the role of sulfur amino acids (Cys or Met) for ferroptosis protection in ECs, we assessed the viability of PECs cultured for 24, 48 and 72 h (h) in Met-, Cys-Cys-, or Met-+Cys-Cys- free medium (Fig. 1A). Our findings indicate that PECs cultured in cystine-free medium (FMCys-Cys) exhibit a marked decrease in viability, underscoring the critical reliance of ECs on extracellular Cys-Cys for survival. On the other hand, PECs show resistance to Met starvation. Notably, PECs cultured in the absence of both Met and Cys-Cys displayed a short-term resistance to cystine starvation (Fig. 1B). The reduction of cell viability was accompanied by an increase in cell death, as measured by propidium iodide (PI) incorporation (Fig. 1C). Moreover, the impact of cystine limitation on cell viability was found to be concentration-dependent (Fig. 1D).

To further explore the robustness of endothelial cysteine metabolism and determine whether this dependency is conserved across EC subtypes, we extended our analysis to additional endothelial models. In addition to human umbilical vein endothelial cells (HUVECs), a widely used in vitro model of vascular endothelium, we included human aortic endothelial cells (HAECs) and mouse pancreatic endothelial cells (MS1) to enhance the diversity of our system. While proliferating (PECs) HAECs exhibited resistance to Cys-Cys starvation (Supplementary Fig. 1A), PECs MS1 were highly susceptible (Supplementary Fig. 1B). As later explained (Supplementary Fig. 4A), this difference may stem from different expression of metabolic enzymes and pathways involved in cysteine uptake and compensation (TSP) supported by the existence and complexity of intra- and inter-organ EC heterogeneity [22, 23].

Next, we examined the contribution of ferroptosis to PECs death caused by Cys-Cys starvation. Thus, ferroptosis was assessed by measuring lipid peroxides. In Cys-Cys- free medium, PECs showed a significant increase in lipid peroxides, confirming how exogenous Cys-Cys is critical in PECs to prevent ferroptosis (Fig. 1E). Additionally, by employing various anti-ferroptotic inhibitors [24]; including ferrostatin 1 (Fer1), deferoxamine (DFO), and liproxstatin (Lpx), we were able to rescue EC survival and lipid peroxidation production (Fig. 1F-G). Similar to Cys-Cys deprivation, treatment with inhibitors of the xCT system such as erastin [25] (Fig. 1H-I) or sulfasalazine [26] (Supplementary Fig. 1C) induces ferroptosis and cell death. These data show that PECs utilize extracellular Cys-Cys to prevent ferroptosis cell death through lipid peroxidation production.

To determine which metabolic pathways are involved in the initiation and execution of ferroptosis in PECs under Cys-Cys limitation, we assessed global metabolic changes induced by Cys-Cys deprivation through liquid chromatography coupled with tandem mass spectrometry (LC-MS/MS) analysis. We measured the relative steady-state levels of a total of 81 polar metabolites, highlighting the top 50 metabolites (Fig. 1J). Among these, 28 metabolites were significantly modulated (padj<0.05), including 10 upregulated and 18 downregulated metabolites (Fig. 1K). Metabolome enrichment analyses revealed a significant increase in metabolites associated with Warburg effect, carnitine synthesis, and glycolysis (Fig. 1L and Supplementary Fig. 1D-E), while a decrease in amino sugar metabolism, spermidine and spermine biosynthesis and glutathione metabolism (Fig. 1M) was observed in FMCys-Cys compared to the control.

The cysteine/GSH/GPX4 axis is a significant asset in controlling ferroptosis [27]. Therefore, we next investigated intracellular Cys and glutathione (GSH) levels in PECs after 24 h of FMCys-Cys treatment. We detected a significant reduction in both Cys and Cys-Cys (Supplementary Fig. 1F-G), as well as a substantial decrease in total levels of GSH and GSSG (Supplementary Fig. 1H-I), including their sum and reduced ratio (Supplementary Fig. 1J-K). Additionally, the sulfur-containing amino acid taurine, derived from cysteine, and its precursor hypotaurine were significantly reduced (Supplementary Fig. 1L-M). Rescue experiments using GSH, GSSG, and taurine were conducted to determine which cysteine-derived metabolite is crucial for preventing ferroptosis in PECs. Ultimately, only GSH was able to rescue cell viability and prevent lipid peroxidation (Fig. 1N-O).

This data shows that PECs are dependent on extracellular Cys-Cys, which is vital for maintaining GSH levels necessary for preventing the accumulation of intracellular peroxides and averting ferroptosis.

### Quiescent ECs evade ferroptosis triggered by cystine deficiency in a SLC7A11-independent manner

**Fig. 2:**
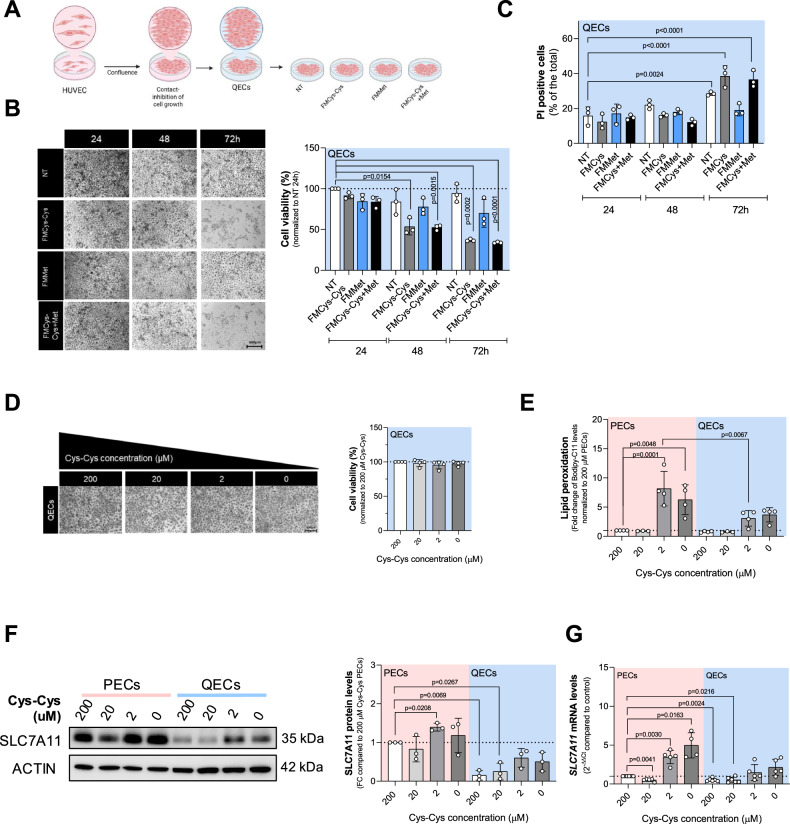

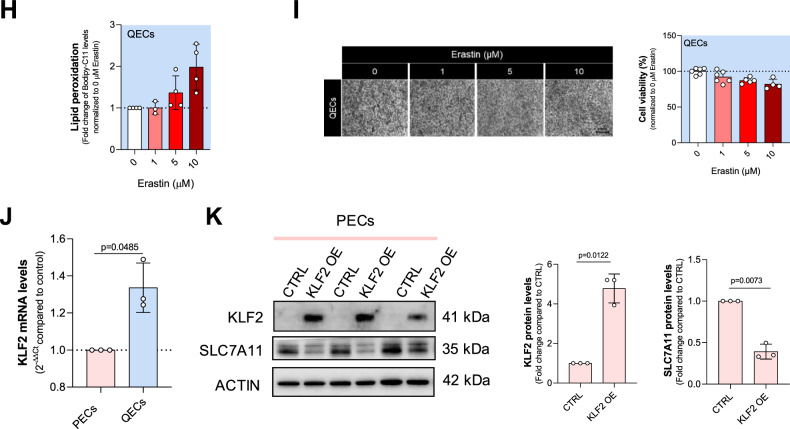
QECs resistance to cystine deprivation is independent from xCT. **A** Diagram explaining the experimental protocol used to achieve quiescent endothelial state and metabolic treatment. **B** Cell viability assay using crystal violet staining on quiescent HUVECs (QECs) grown in cystine-(FMCys-Cys), methionine- (FMMet) or both-(FMCys-Cys+Met) free medium for 24, 48 and 72 h. At least 3 biological replicates were quantified and the percentage of living cells was calculated using cells grown in 200 μM cystine and methionine 100 µM as a reference. Statistical significance is given by one sample t-test and reported as a p-value in the figure. **C** Cell death analysis using PI staining on quiescent HUVECs (QECs) grown in cystine-(FMCys-Cys), methionine- (FMMet) or both-(FMCys-Cys+Met) free medium for 24, 48 and 72 h. Three biological replicates were quantified, and the percentage of living cells was calculated using cells grown in 200 μM cystine as a reference. Statistical significance is given by one sample t-test and reported as a p-value in the figure. **D** Cell viability assay using crystal violet staining on quiescent HUVECs (QECs) grown in different cystine concentration (0; 2; 20; 200 μM) for 24 h. At least 3 biological replicates were quantified and the percentage of living cells was calculated using cells grown in 200 μM cystine as a reference. Statistical significance is given by one sample t-test and reported as a p-value in the figure. **E** Lipid peroxidation levels measured by Bodipy-C11 FACS in PECs and QECs grown in different cystine concentration (0; 2; 20; 200 μM) for 24 h. The ratio between oxidized and reduced Bodipy-C11 was measured and reported as fold change over control (PECs grown in 200 μM cystine). Statistical significance is given by one sample t-test and reported as adjusted p-value in the figure. **F** Western blot analysis for cystine transporter SLC7A11 under different cystine concentration (0; 2; 20; 200 μM) for 24 h in both PECs and QECs. ACTIN was used as loading control and band intensities were normalized on control condition (PECs grown in 200 μM cystine). Statistical significance is given by one sample t-test and reported as a p-value in the figure. **G** The mRNA levels of *SLC7A11* under different cystine concentrations (0; 2; 20; 200 μM) for 24 h in both PECs and QECs. *ACTIN* was used as control. Statistical significance is given by one sample t-test and reported as a p-value in the figure. **H** Lipid peroxidation levels measured by Bodipy-C11 FACS in QECs treated with increasing concentration (0; 1; 5; 10 μM) of erastin for 24 h. The ratio between oxidized and reduced Bodipy-C11 was measured and reported as fold change over control (PECs grown in 200 μM cystine). Statistical significance is given by one sample t-test and reported as adjusted p-value in the figure. **I** Cell viability assay using crystal violet staining on QECs treated with increasing concentration (0; 1; 5; 10 μM) of erastin for 24 h. At least 3 biological replicates were quantified and the percentage of living cells was calculated using untreated (NT) condition as a reference. Statistical significance is given by one sample t-test and reported as a p-value in the figure. **J** The mRNA levels of *KLF2* in PECs and QECs in NT condition. *ACTIN* was used as control. Statistical significance is given by one sample t-test and reported as a p-value in the figure. **K** Protein levels of KLF2 and SLC7A11 of PECs overexpressing KLF2. ACTIN was used as control. Statistical significance is given by one sample t-test and reported as a p-value in the figure.

In adulthood, ECs are primarily present in a quiescent state. This quiescent state is characterized by minimal or absent endothelial proliferation and migration, minimal or no vascular leakage across the endothelial barrier, and minimal (or completely absent) expression of leukocyte adhesion molecules [3]. However, whether PECs and QECs show differential sensitivity to ferroptosis under sulfur amino acid limitation has never been explored. To address this, we investigated whether QECs engage the cysteine/GSH/GPX4 axis to resist ferroptosis. To induce a quiescent state, PECs were grown and maintained at confluence to promote contact inhibition and suppress cell proliferation (Fig. 2A). We validated the quiescent phenotype of ECs by analyzing cell cycle markers and Notch signaling in our model [10]. Immunoblot analysis showed a decrease in the cell cycle markers, cyclin A and phospho-histone 3 (Ser10), and an increase in Notch signaling in QECs compared to PECs. Additionally, Hairy and enhancer of split 1 (Hes1) mRNA, a downstream target of Notch signaling, was increased in QECs compared to PECs (Supplementary Fig. 2A-B). Once we established bona fide QECs, cells were starved for Met (FMMet), Cys-Cys (FMCys-Cys), or both (FMCys-Cys+Met) for 24, 48, and 72 h, and cell viability was analyzed. QECs showed resistance to Cys-Cys deprivation compared to PECs. Similar to PECs, the depletion of Met showed no significant effect (Figs. 2B and 1B). Consistent results were observed in cell death assays, as indicated by PI incorporation (Fig. 2C). QECs viability remained unaffected under decreasing extracellular concentrations of Cys-Cys (Fig. 2D). Additionally, QECs produced significantly lower levels of lipid peroxides than PECs when cultured under equivalent Cys-Cys deprived conditions that were not able to induce cell death (Fig. 2E). Alongside HUVECs, we investigated quiescent HAECs and MS1. Quiescent (QEC) HAECs exhibited resistance to Cys-Cys starvation (Supplementary Fig. 2C), while quiescent MS1 were highly susceptible (Supplementary Fig. 2D).

Considering that SLC7A11, the functional member of the xCT transporter, is a major transporter of Cys-Cys, we investigated whether the differential cystine sensitivity of ECs is associated with SCL7A11 expression and activity. We evaluated SCL7A11 protein levels by immunoblot analysis. Both PECs and QECs increased SCL7A11 expression at low Cys-Cys levels, indicating a compensatory mechanism; however, QECs expressed lower levels of the transporter at physiological (200 and 20 μM) extracellular cystine concentrations compared to PECs (Fig. 2F). These changes in SCL7A11 protein levels were accompanied by transcriptional modulation of the gene (Fig. 2G). Considering the differential expression of SCL7A11, we also measured the uptake of cystine-FITC-A in PECs and QECs. Our results showed reduced Cys-Cys consumption in QECs compared to proliferating ones (Fig. Supplementary. 2E). Next, we determined the erastin sensitivity of QECs. Consistent with the previous results, QECs showed no significative induction of lipid peroxidation production and cell death under erastin treatment (Fig. 2H-I). A similar behavior was observed using sulfasalazine (Supplementary Fig. 2F). These data indicate that QECs are resistant to ferroptosis induced by Cys-Cys limitation possibly explained by a lower dependency on extracellular Cys-Cys by quiescent compared to proliferating ECs. The transcriptional factor Lung Krüppel-like factor (KLF2) activates a transcriptional program that establishes functional quiescence and differentiation of the endothelium [28]. We then evaluated whether KLF2 is differently expressed in QECs compared to PECs, and whether its expression can modulate SCL7A11 and dependency on exogenous cystine. QECs exhibited a significant increase in KLF2 levels compared to PECs (Fig. 2J). Furthermore, KLF2 overexpression in PECs resulted in a significant reduction in SLC7A11 expression (Fig. 2K), indicating that KLF2 regulates SLC7A11 in QECs. These findings highlight KLF2 as a key regulator of EC quiescence and ferroptosis resistance. By suppressing SLC7A11 expression, KLF2 may reduce cystine dependency in QECs, contributing to their resilience under conditions of cystine limitation.

### Ferroptosis resistance in QECs is primarily mediated through the GPX4 pathway rather than FSP1

**Fig. 3:**
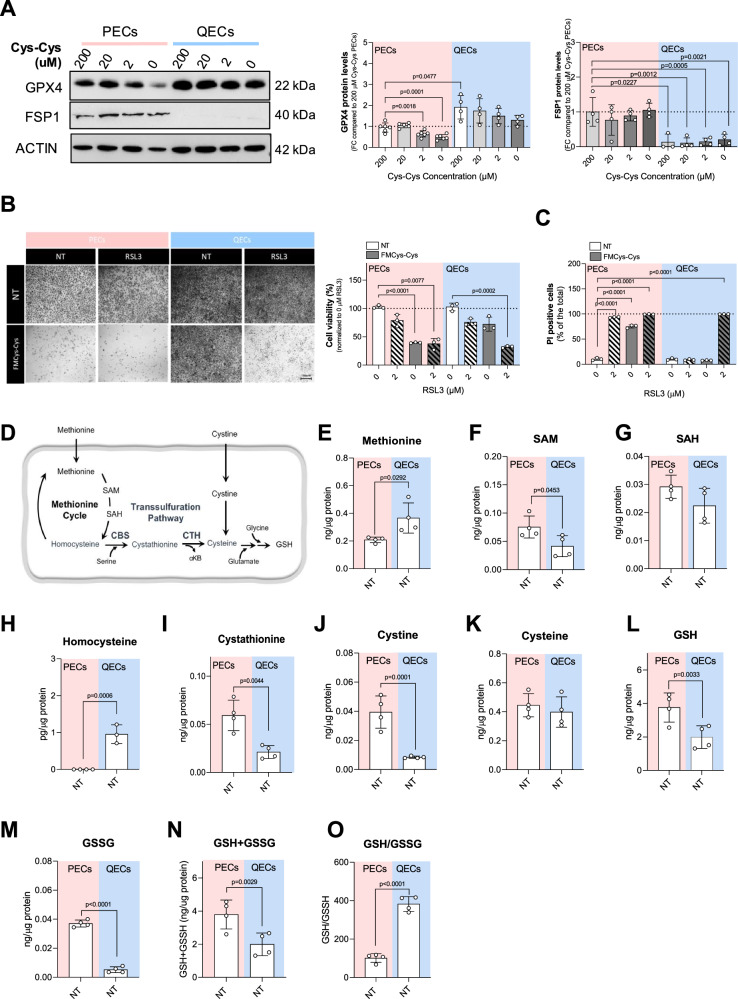
QECs survival under cystine limitation is dependent on GPX4. **A** Western blot analysis for GPX4 and FSP1 under different cystine concentration (0; 2; 20; 200 μM) for 24 h in both PECs and QECs. ACTIN was used as loading control and band intensities were normalized on control condition (PECs grown in 200 μM cystine). Statistical significance is given by one sample t-test and reported as a p-value in the figure. **B** Cell viability assay using crystal violet staining on PECs and QECs grown in control medium (200 μM cystine) or FMCys-Cys and treated with the GPX4 inhibitor RLS3 (100 nM) for 24 h. At least 3 biological replicates were quantified and the percentage of living cells was calculated using untreated (NT) condition as a reference. Statistical significance is given by one sample t-test and reported as a p-value in the figure. **C** Cell death analysis using PI staining on PECs and QECs grown in control medium (200 μM cystine) or FMCys-Cys and treated with the GPX4 inhibitor RLS3 (100 nM) for 24 h. At least 3 biological replicates were quantified and the percentage of living cells was calculated using untreated (NT) condition as a reference. Statistical significance is given by one sample t-test and reported as a p-value in the figure. **D** Schematic overview of synthesis of glutathione derived from sulfur-amino acids. Intracellular levels of methionine (**E**), S-adenosyl methionine (SAM) (**F**), S-adenosyl homocysteine (SAH) (**G**), homocysteine (**H**), cystathionine (**I**), cystine (**J**), cysteine (**K**), reduced glutathione (GSH) (**L**), and oxidized glutathione (GSSG) (**M**), in proliferating and quiescent ECs grown in 200 μM cystine medium (NT) measured by mass-spectrometry. **N** Total intracellular levels of glutathione (GSH + GSSG) and (**O**) Glutathione ratio (GSH/GSSG) in PECs and QECs grown in 200 μM cystine medium (NT) measured by mass-spectrometry. Statistical significance is given by unpaired t-test and reported as adjusted p-value in the figure.

GPX4 and FSP1 are two crucial, parallel ferroptosis suppressors that work by reducing lipid peroxidation, which drives cell death. GPX4 is a glutathione-dependent system that directly neutralizes lipid hydroperoxides using reduced glutathione (GSH). In contrast, FSP1 is a glutathione-independent system that functions as a coenzyme Q10 (CoQ10) reductase, regenerating CoQ10 to its active form (ubiquinol) to trap radical species that cause lipid peroxidation [29]. Next, we determined how GPX4 and FSP1 are regulated in PECs versus QECs. QECs showed increased levels of GPX4 compared to PECs at physiological concentrations of Cys-Cys (200 µg/mL), while PECs showed a significant decrease during cystine starvation. Also, FSP1 was differentially modulated in QECs compared to PECs. Quiescent cells expressed less FSP1 compared to PECs at physiological levels of Cys-Cys (Fig. 3A). The differential protein expression of GPX4 and FSP1 in PECs vs. QECs appears to be associated with post-transcriptional modulation, given that no changes were detected in their transcript levels (Supplementary Fig. 3A–B). Then we analyzed the sensitivity of PECs and QECs to RSL3, a chemical inhibitor of GPX4 [30]. While RSL3 appears to be insufficient to induce ferroptosis in QECs, in FMCys-Cys QECs exhibited an evident sensitivity to RSL3 (Fig. 3B-C). Altogether, these data show that QECs use the GSH/GPX4 axis to protect against ferroptosis under Cys-Cys limitation. However, how quiescent cells maintain intracellular Cys levels necessary for GSH production remains unknown.

To address this point, we analyzed the sulfur metabolites involved in the synthesis of Cys and GSH production by LC-MS/MS analysis (Fig. 3D). While some metabolites, including S-adenosylmethionine (SAM) and cystathionine, were decreased, QECs showed a significant increase in methionine and homocysteine (Hcy), precursors necessary for the de novo synthesis of cysteine through the TSP (Fig. 3E-I). Quiescent ECs exhibited similar intracellular levels of Cys compared to proliferating ones, but a significant decrease in Cys-Cys, confirming a reduced uptake of Cys-Cys (Fig. 3J-K). Although the total levels of glutathione (GSH + GSS) were reduced in QECs, the reduced/oxidized (GSH/GSSG) ratio was significantly higher in quiescent cells (Fig. 3L-O). These results suggest a crucial role of TSP in maintaining the cysteine requirement of QECs to protect themselves against Cys-Cys limitation.

### Quiescent ECs utilize the TSP to survive to cystine limitation

**Fig. 4:**
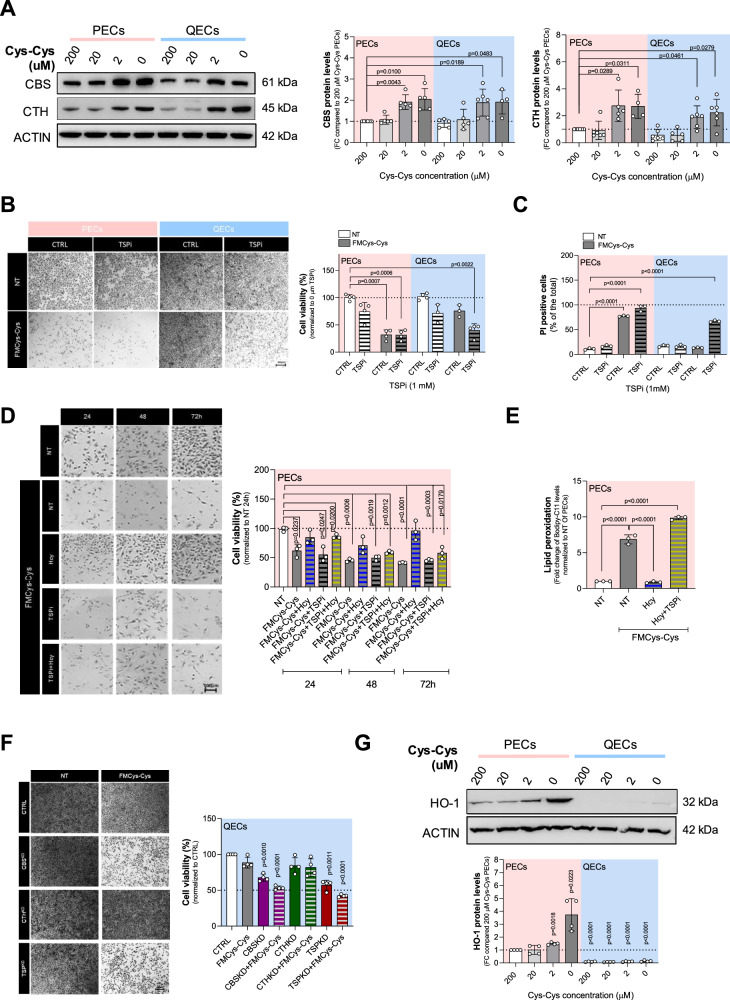
The transsulfuration pathway (TSP) is required for QECs’ survival and ferroptosis resistance during cystine deprivation. **A** Western blot analysis for TSP enzymes: CBS and CTH, under different cystine concentrations (0; 2; 20; 200 μM) for 24 h in both PECs and QECs. ACTIN was used as loading control and band intensities were normalized on control condition (PECs grown in 200 μM cystine). Statistical significance is given by one sample t-test and reported as a p-value in the figure. **B** Cell viability assay using crystal violet staining on PECs and QECs grown in control medium (200 μM cystine) or FMCys-Cys and treated with TSP inhibitors (TSPi, 1 mM TFA + 1 mM PAG) for 24 h. Statistical significance is given by one sample t-test and reported as a p-value in the figure. **C** Cell death analysis using PI staining on PECs and QECs grown in control medium (200 μM cystine) or FMCys-Cys and treated with TSP inhibitors (TSPi, 1 mM TFA + 1 mM PAG) for 24 h. Statistical significance is given by one sample t-test and reported as a p-value in the figure. **D** Cell viability assay using crystal violet staining on proliferating HUVECs grown over 3 days in FMCys-Cys supplemented with 1 mM Hcy, TSPi (1 mM TFA + 1 mM PAG) or both. Statistical significance is given by one sample t-test and reported as a p-value in the figure. **E** Lipid peroxidation levels measured by Bodipy-C11 FACS in PECs grown in FMCys-Cys and treated with 1 mM Hcy or Hcy + TSPi (1 mM TFA + 1 mM PAG) for 24 h. The ratio between oxidized and reduced Bodipy-C11 was measured and reported as fold change over control (PECs grown in 200 μM cystine, also indicated as NT). Statistical significance is given by one sample t-test and reported as adjusted p-value in the figure. **F** Cell viability assay using crystal violet staining on QECs grown in control medium (200 μM cystine) or FMCys-Cys for 24 h and knocked-down for CBS, CTH or both enzymes. Statistical significance is given by one sample t-test and reported as a p-value in the figure. **G** Western blot analysis for HO-1 under different cystine concentration (0; 2; 20; 200 μM) for 24 h in both PECs and QECs. ACTIN was used as loading control and band intensities were normalized on control condition (PECs grown in 200 μM cystine). Statistical significance is given by one sample t-test and reported as a p-value in the figure.

Since TSP activity is involved in de novo Cys synthesis, we evaluated whether this pathway is responsible for the lack of drop in Cys levels in QECs. Thus, we evaluated the protein levels of CBS and CTH, the key enzymes involved in cysteine synthesis from homocysteine (Fig. 3D). Both types of ECs showed a similar response under Cys-Cys limitation increasing the levels of TSP enzymes (Fig. 4A). We also compared the levels of SLC7A11, TSP and GPX4 enzymes in proliferating and quiescent HAEC and MS1 (Supplementary Fig. 4A). Compared to HUVEC, proliferating HAECs express higher levels of SCL7A11 and CBS, suggesting they can utilize TSP pathway to sustain growth under cysteine-deficient conditions. In contrast, MS1 cells show elevated, unmodulated SLC7A11 expression as well as undetectable levels of TSP proteins, indicating a firm reliance on extracellular cysteine uptake.

We then examined whether TSP inhibition impacts on cell death under Cys-Cys deprivation, using TSP inhibitors (TSPi), including the CBS inhibitor trifluoroalanine (TFA) and the CTH inhibitor propargylglycine (PAG) [31]. TSPi treatment alone did not alter viability or induce cell death in either PECs or QECs. However, under cystine-starved conditions, TSPi significantly increases cell death in QECs (Fig. 4B-C). Similarly knock down of both enzymes of TSP pathway, CTH and CBS, (TSP^KD^) (Supplementary Fig. 4B-C), demonstrates that TSP plays a protective role in QECs under cystine depletion.

Next, we explored the opposite scenario and investigated whether the increased availability of TSP substrate could mitigate ferroptotic cell death in PECs induced by cystine limitation. To test this, PECs cultured in FMCys-Cys were treated with Hcy, either in the presence or absence of TSPi. Our results showed that Hcy was able to rescue FMCys-Cys-induced cell death and lipid peroxidation and such rescue effect were blunted by adding TSPi (Fig. 4D-E). These data indicate that increasing Hcy levels under Cys-Cys limitation is sufficient to restore ferroptosis resistance in PECs through TSP.

Considering that Hcy is not only used by TSP for cysteine synthesis but also hydrogen sulfide (H₂S) production, we evaluated whether H₂S can rescue cell death. Therefore, PECs deprived of Cys-Cys were incubated with increasing concentrations of NaHS, and cell viability was analyzed (Supplementary Fig. 4D). Similarly to Hcy, H₂S was able to rescue ECs under cystine limitation, indicating a crucial role for TSP in protecting PECs under Cys-Cys limitation.

Then, we explored which TSP enzyme is crucial for Cys-Cys resistance in QECs. QECs were knocked down for CBS, CTH, or both, and cultured in FMCys-Cys for 24 h. The quiescent cells showed a dependency on CBS activity to resist Cys-Cys starvation (Fig. 4F). Given that CBS is a heme-binding protein that can be inhibited by carbon monoxide (CO) and nitric oxide (NO), we determined the protein levels of the inducible heme oxygenase 1 (HO-1), which produces endogenous CO [32]. Immunoblot analysis showed a clear reduction of HO-1 in QECs, suggesting the possibility that CBS is more active in QECs compared to PECs (Fig. 4G).

To understand the role of cystine utilization during in vivo angiogenesis, we employed a postnatal retina angiogenesis model. This model help to study the response of PECs and QECs, with PECs primarily located at the vascular front and QECs localized in the rear region [33]. Next, we treated pups at P5 with erastin or TSPi, and after 24 h we measured the angiogenic properties of the retinal vasculature, including EC area, branch points, and vessel length per field in both front and rear regions (Supplementary Fig. 4E). Also, the number of ECs was analyzed by ERG staining, a specific nuclear marker of ECs. Our results show that in comparison to control and TSPi-treated animals, erastin induced a significant reduction in the EC area and number of ERG+ cells at the angiogenic front but not in the rear region (Supplementary Fig. 4F-G). These data confirmed the crucial role of cystine uptake to support survival in PECs. Conversely, TSPi did not induce any visible phenotype in both front or rear region, suggesting the possibility that TSP play a different role in vivo. Indeed, when we measure the transcript levels of SCL7A11 and TSP enzymes in mouse retinal and micropulmonary ECs (MPECs) both EC types displayed high levels of the antiporter *Slc7a11* compared to the TSP enzymes, *Cth* and *Cbs* (Supplementary Fig. 4H-I). To ensure that TSP inhibition does not adversely affect retinal vascular development, we selectively knocked down the *Cth* gene in endothelial cells using *Cth*^fl/fl^*; Cdh5-CreERT2* mice (Supplementary Fig. 4J). Following hydroxytamoxifen administration, *Cth* expression was significantly reduced in ECs of inducible knockout mice compared to *Cth*^*fl/fl*^ controls (Supplementary Fig. 4K). Retinal vasculature analysis at postnatal day six, using the blood vessel marker isolectin B4 staining, revealed no significant alterations (Supplementary Fig. 4L), consistent with the outcomes observed following pharmacological TSP inhibition (Supplementary Fig. 4F).

Altogether, these results show that cultured PECs rely on extracellular Cys-Cys to synthesize the GSH, which is essential for protection against ferroptosis. Under conditions of Cys-Cys deprivation, the TSP is insufficient to sustain the cysteine levels required for GSH production. In contrast, QECs exhibit higher availability of Hcy, supporting cysteine synthesis via the TSP, and display lower levels of HO-1. These features enable QECs to maintain GSH synthesis and resist ferroptosis during Cys-Cys starvation.

### PECs rewire cysteine metabolism to protect themselves from ferroptosis under hypoxic conditions

**Fig. 5:**
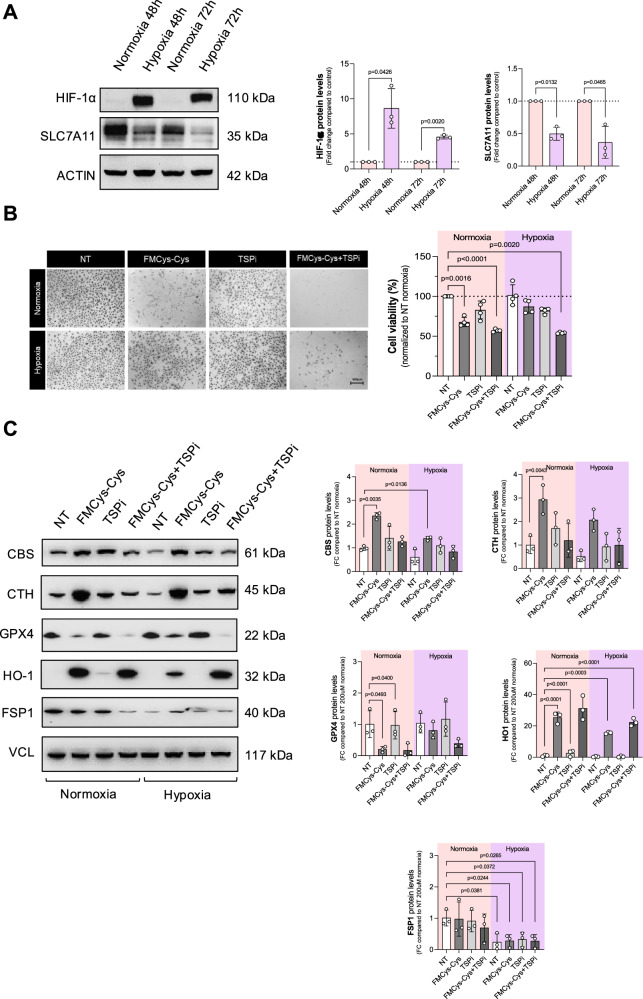
Hypoxia promotes metabolic rewiring in PECs, resulting in resistance to cysteine deprivation. **A** Western blot analysis for HIF-1α and SCL7A11 in HUVECs exposed to hypoxia (1% O_2_) or normoxia (21% O_2_) for 48 and 72 h. ACTIN was used as loading control, and band intensities were normalized on control condition (Normoxia 24 h). Statistical significance is given by one sample t-test and reported as a p-value in the figure. **B** Cell viability assay using crystal violet staining on PECs exposed to hypoxia (1% O_2_) or normoxia (21% O_2_) for 48 h, incubated in control medium (200 μM cystine) or FMCys-Cys for 24 h in presence or absence of TSPi. Statistical significance is given by one sample t-test and reported as a p-value in the figure. **C** Western blot analysis for CBS, CTH, GPX4, HO-1 and FSP1 in PECs exposed to hypoxia (1% O_2_) or normoxia (21% O_2_) for 48 h, incubated in control medium (200 μM cystine, NT) or FMCys-Cys for 24 h. Statistical significance is given by one sample t-test and reported as a p-value in the figure.

Exposure of the ECs to hypoxia, and the decrease in oxygen supply can trigger an endothelial response critical in inflammatory diseases, tumorigenesis, and the microvascular damage associated with aging. While it has been shown that hypoxia induces a severe metabolic shift in ECs [34, 35], it remains unexplored whether PECs under chronic hypoxia rewire cysteine metabolism to protect themselves from ferroptosis. To evaluate this, ECs were exposed to standard growth conditions, where the oxygen concentration (%O₂) ranged between 18 and 21%, commonly referred to as ‘normoxic conditions’; as well as to severe hypoxia (1% O_2_) for 48 and 72 h. We verified the hypoxic condition by measuring hypoxia-inducible factor 1α (HIF-1α) levels. Immunoblotting analysis revealed an accumulation of HIF-1α under hypoxic conditions compared to normoxia, confirming the hypoxic state (Fig. 5A). Next, the protein levels of SCL7A11 were evaluated by immunoblotting. Our results showed that low levels of O_2_ induced a significant decrease in SCL7A11 like QECs (Fig. 5A). At this point, we hypothesized that PECs exposed to low levels of O_2_ rewire their cysteine metabolism to protect themselves from ferroptosis. To validate this hypothesis, we incubated PECs under normoxic and hypoxic conditions, and then, we cultured the cells in Cys-Cys deprivation. Interestingly, PECs exposed to hypoxia showed resistance to Cys-Cys starvation (Fig. 5B). ECs exposed to hypoxia showed an increase of GPX4 after Cys-Cys limitation. While TSP levels did not change under stress conditions, we noticed a significant decrease of HO-1 during hypoxia in Cys-Cys limitation that may explain increased CBS activity and TSP-mediated protective function (Fig. 5C). Interestingly, similarly to QECs, hypoxic ECs showed a reduction in FSP1 (Fig. 5C). ECs treated with dimethyloxalylglycine (DMOG), a prolyl hydroxylase inhibitor that can mimic the effect of hypoxia, also showed resistance to Cys-Cys limitation (Supplementary Fig. 5A-B).

These data indicate that chronic and severe hypoxia inhibits ferroptosis in proliferating ECs, allowing the cells to survive under cystine limitations, suggesting an adaptive survival mechanism when metabolites and O_2_ are limited.

### NRF2-driven TSP activation promotes ferroptosis resistance in QECs under cystine starvation

**Fig. 6:**
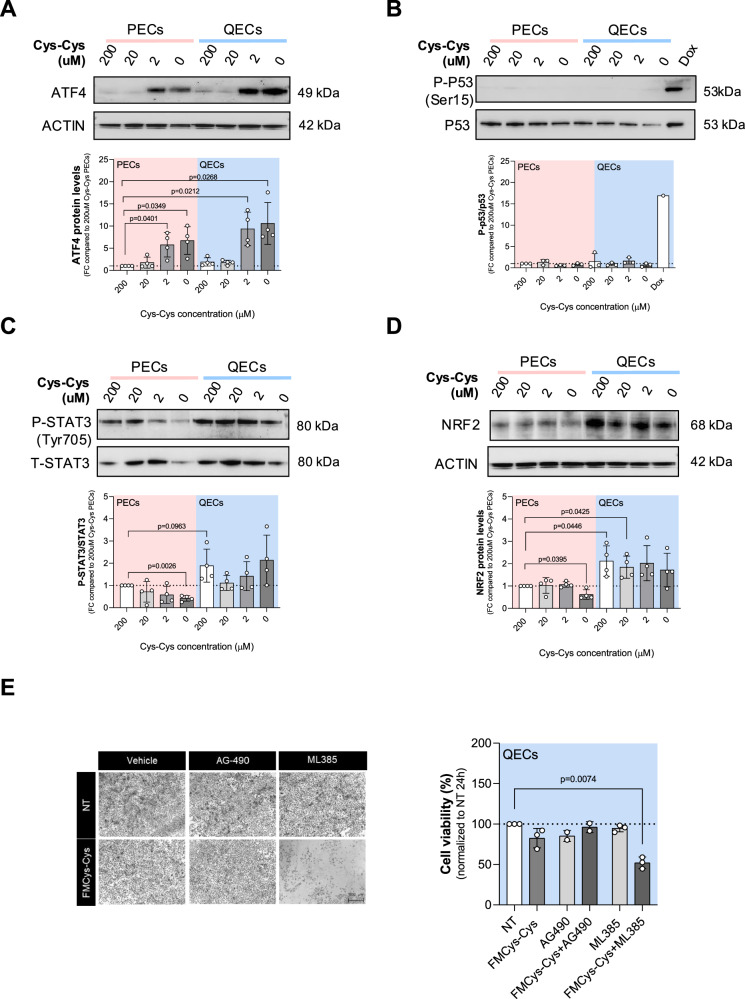

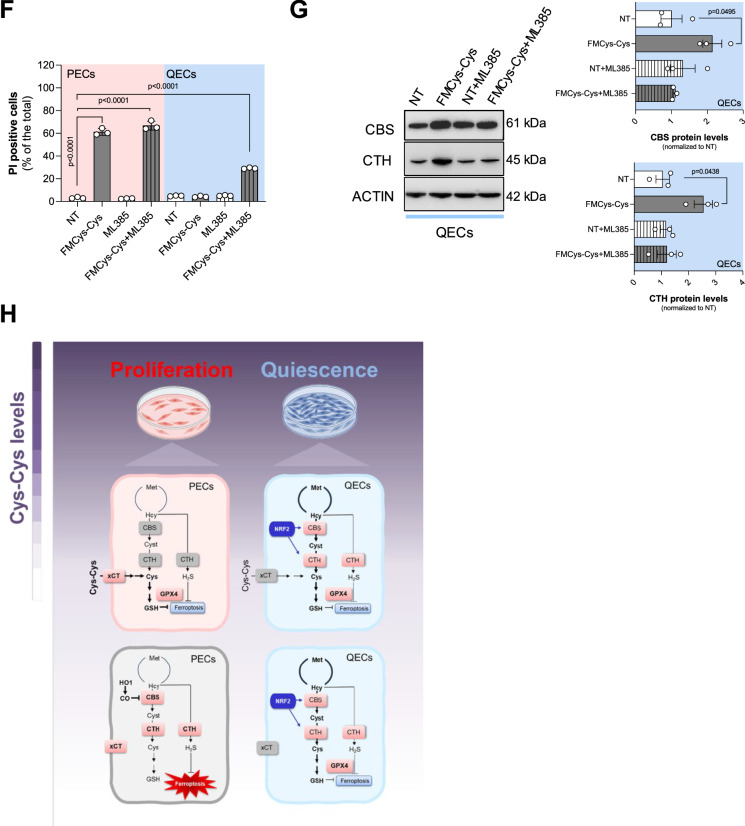
NRF2 drives TSP-dependent resistance in QECs under cystine deprivation. Western blot analysis of transcriptional factors: ATF4 (**A**), P-P53 (Ser15)/P53 (**B**), P-STAT3 (Tyr705)/STAT3 (**C**) and NRF2 (**D**), under different cystine concentrations (0; 2; 20; 200 μM) for 24 h in both PECs and QECs. ACTIN was used as loading control. At least 3 biological replicates were quantified and band intensities were normalized on control condition (PECs grown in 200 μM cystine). Lysates of HUVECs treated with 20 µM doxycycline for 24 h were used as positive control for p53 activation. Statistical significance is given by one sample t-test and reported as a p-value in the figure. **E** Cell viability assay using crystal violet staining on QECs grown in control medium (200 μM cystine) or FMCys-Cys for 24 h, and treated with 40 µM AG490 (STAT3 signalling inhibitor) or 7 µM of ML385 (NRF2 inhibitor). At least 3 biological replicates were performed. Statistical significance is given by one sample t-test and reported as a p-value in the figure. **F** Cell death assay using PI staining on PECs and QECs grown in control medium (200 μM cystine) or FMCys-Cys for 24 h, and treated with 7 µM of ML385. At least 3 biological replicates were performed. Statistical significance is given by one sample t-test and reported as a p-value in the figure. **G** Analysis of TSP enzyme by immunoblotting in QECs grown in control medium (200 μM cystine) or FMCys-Cys for 24 h, and treated with 7 µM of ML385. At least 3 biological replicates were performed. Statistical significance is given by one sample t-test and reported as a p-value in the figure. **H** Schematic overview of the distinct metabolic reliance on the cysteine/GSH/GPX4 pathway in PECs and QECs under varying cystine availability.

To decipher how QECs are able to resist to cystine deprivation by TSP activity, we evaluated the expression of transcription factors known to regulate metabolic stress conditions in cells. These included the integrated stress response (ISR) responder ATF4 (activating transcriptional factor 4) [36], the controller of damaged cells p53 [37], the signal transducer and activator of transcription STAT3 [38], and the oxidative stress sensor NRF2 (nuclear factor erythroid 2-related factor 2) [39]. ATF4 was found upregulated under cystine-limiting conditions, consistent with previous findings [16] (Fig. 6A). However, the basal and cystine-starved ATF4 levels did not significantly differ between proliferating and quiescent ECs, indicating that ATF4 induction alone does not explain the differential cystine dependency observed in these two cellular states. Similarly, p53 signaling remained unchanged in PECs and QECs (Fig. 6B). Conversely, QECs exhibited a significant increase in phosphorylated (on Tyr705) STAT3 (P-STAT3/STAT3 ratio) and NRF2 levels compared to PECs (Fig. 6C-D). To identify which pathway contributes most to QEC tolerance to Cys-Cys limitation, we treated QECs with specific inhibitors targeting STAT3 and NRF2 activity: AG-490 [40] and ML385 [41], respectively. While STAT3 inhibition did not sensitize QECs to Cys-Cys deprivation (Fig. 6E), blocking NRF2 resulted in a marked increase in Cys-Cys starvation-induced cell death after 24 hours of treatment (Fig. 6E, F). The increase in cell death due to ML385 was accompanied by a reduction in CBS and CTH induction under Cys-Cys-limiting conditions (Fig. 6G), indicating that TSP induction via NRF2 contributes to resistance against Cys-Cys starvation-induced ferroptosis. Overall, these findings demonstrate that TSP, induced by NRF2 under cystine-limiting conditions, plays a crucial role in promoting quiescent cell tolerance to ferroptosis triggered by cystine deprivation (Fig. 6H).

## Discussion

EC metabolism drives angiogenesis [42, 43]. Indeed, metabolic pathways, such as glycolysis [7], fatty acid oxidation [10], pentose phosphate pathway [44] and glutamine metabolism [8, 9], have essential roles during vessel formation and maturation. Limited information is available on the metabolic pathways regulating the redox state in ECs, which might regulate ferroptosis resistance [13]. This type of non-apoptotic-regulated cell death is governed by three different metabolic-antioxidant axes: the cysteine/GSH/GPX4 axis [45, 46], the GCH1/BH4/DHFR axis [47], and the FSP1/CoQ10 axis [48]. Recognized as the mainstay in ferroptosis control [49], understanding whether the cysteine/GSH/GPX4 axis is crucial to support the survival of PECs and is differentially modulated in QECs remains unclear. Here, we demonstrate that PECs display a ferroptotic-sensitive program governed by cysteine/GSH/GPX4 axis. Proliferating cells consume actively extracellular cystine to produce GSH that GPX4 can use to decrease peroxidized membrane lipids and protect from ferroptosis. On the contrary, QECs display protection against limited cystine availability. This ferroptosis-resistant program is associated with a mechanism that blunts uptake of cysteine while activating TSP, favoring cysteine de novo synthesis and protection by the cysteine/GSH/GPX4 axis.

The discrepancy between in vitro and in vivo results, evidenced by the absence of a strong phenotype in mice treated with erastin or knock-out for *Cth* can be explained by several reasons. It is reported that in vitro settings (cultured medium), cysteine is readily oxidized to cystine, which is transported via the xCT system. On the contrary, in vivo conditions (such as plasma and extracellular body fluids), cysteine predominantly exists in its reduced form and is taken up by neutral amino acid transporters that function independently of the xCT system [50, 51]. This alternative transport mechanism likely attenuates the impact of xCT inhibition in vivo, thereby reducing the phenotype associated with its blockade and with the use of TSP pathway. Moreover, the uptake and catabolism of cysteine-rich extracellular proteins, such as albumin, may provide an additional source of cysteine in the body [52], thereby buffering the dependency of ECs in the retinal vasculature on extracellular cystine. These compensatory mechanisms are further supported by the observation that *Slc7A11* knockout mice are viable and non-lethal [53].

In our study, we identified an original mechanism used by quiescent ECs to protect themselves from a lack of cystine availability that relies on the NRF2 pathway. The NRF2 pathway is activated by stress conditions like oxidative stress and involves its release from the inhibitor KEAP1, translocation into the nucleus, and the induction of genes that produce antioxidant and detoxification enzymes [54]. This activation protects against damage from free radicals and metabolic stresses by upregulating cellular defense systems. We showed that QECs rely on the NRF2 pathway to survive in cystine-deprived conditions, possibly through the transcriptional activation of TSP. Interestingly, a putative antioxidant response element (ARE) has been reported in the human CBS promoter region [55].

In conclusion, we demonstrated that the difference in TSP utilization between proliferating and quiescent ECs allows the latter to cope with lipid oxidation triggered by cystine deprivation. Additionally, our study revealed that hypoxic reprogramming of the TSP pathway allows PECs to better resist cystine limitation. Thus, our findings indicate TSP as a rational drug target for anti-angiogenesis therapy in different disease settings.

### Limitation of the study

This work has potential limitations. The study does not address the pathological aspect of transsulfuration pathway impairment in vivo, requiring speculation on the possible implications of TSP inhibition in cancer vasculature. To further exploit the therapeutic potential of our findings, it would be appropriate to design a specific study to answer the role TSP plays in tumor endothelial cells pre- and post-angiogenic switch. Conceptually, total cysteine deprivation is an extreme condition for which there is no data available on how often it occurs. It would be useful to investigate the extracellular levels of various metabolites, including gas signaling molecules, in the tumor microenvironment compared to the corresponding healthy tissue.

## Materials and methods

### Primary endothelial cells (ECs) culture

Human umbilical vein endothelial cells (HUVECs) were purchased from Lonza (cat. N° 00191027). And, cultured in M199 medium (Thermo, cat. N°.11150059) supplemented with 20% heat-inactivated fetal bovine serum (FBS) (Carlo Erba Reagents, cat. N°. ECS5000L), 1 mg/mL heparin, 0.2% bovine brain extract (BBE, Lonza cat. N°. CC-4098), 100 U/mL penicillin and 100 μg/mL streptomycin. HUVECs were cultured until the sixth passage in 0.2% pre-coated gelatin (Sigma, cat. N°G2500) plates. Human aortic endothelial cells (HAECs) were purchased form American Type Culture Collection (ATCC, cat. N° CRL-4052) and cultured in Endothelial Cell Growth Medium 2 (PromoCell) supplemented with 100 U/ml penicillin and 100 μg/mL streptomycin. MS1 were purchased from ATCC (cat. N° CRL-2279) and maintained in DMEM 10% FBS supplemented with 100 U/ml penicillin and 100 μg/mL streptomycin. All cells were cultured in a humidified incubator at 37 °C with 5% CO₂, and regularly tested negative for mycoplasma.

### Free-medium cystine medium

Free-medium cystine (FMCys-Cys) was prepared using Dulbecco’s Modified Eagle’s Medium (Thermo, cat. N°. 21013) supplemented with 20% dialyzed fetal bovine serum (dFBS) (Carlo Erba Reagents, cat. N° 26400044), 1 mg/mL heparin, 0.2% bovine brain extract (BBE), 100 U/mL penicillin, 100 μg/mL streptomycin, 2 mM glutamine, 200 μM aspartate, 100 μM methionine. Similarly, free-medium methionine (FMMet) was lacking only the amino acid methionine. While the respective control medium was prepared in the same way and supplemented with 200 μM cystine (Cys-Cys) and 0.5 μM cysteine (Cys).

### Quiescence induction of endothelial cells

Endothelial cells were induced into quiescence through contact-inhibition. To that, 1 × 10^5^ HUVECs were seeded in a 6 cm dish in complete M199 medium, refreshing the medium every 2 days. When the confluence was reached, the medium was replaced with EGM2 (Lonza, cat. N°. CC-3162) and half of the plates were split 1:6 to obtain proliferating cells. After 48 h, the medium was replaced with experimental medium FMCys-Cys, or control medium (Cys and Cys-Cys added), and both proliferating endothelial cells (PECs) and quiescent endothelial cells (QECs) were treated. This protocol has been adapted by Kalucka et al.

### Hypoxia

Cells were incubated under hypoxic conditions (1% O_2_) in an Eppendorf Galaxy 48 R incubator or under standard growing conditions (21% O_2_, referred to as “normoxia”) in Esco CelMate® CO₂ incubator. Temperature (37 °C) and humidity with 5% CO_2_ and nitrogen balance were maintained in each incubator.

### Lentiviral production and silencing in ECs

Lentiviral vectors containing a KLF2-expressing construct, in which the human KLF2 gene was inserted downstream of the PGK promoter in the pRRL-cPPT-PGK-MCS-PRE-SIN backbone, a short hairpin against CBS (shCBS, 5’-CCGTCAGACCAA GTGGCAAA-3’), CTH (shCTH, sigma, TRCN0000078263) or a scramble as a control (pLKO, Addgene #17920), were produced in HEK293T (ATCC, CRL-11268) cells with packaging vectors pMD2.G (VSV-G envelope, Addgene #12259), pMDLg/pRRE (Gag/Pol, Addgene #12251), and pRSV-Rev (Rev, Addgene #12253) by polyethylenimine (PEI)-mediated transfection. A number of 4×10^6^ HEK293T cells were plated in a 10 cm dish, and 48 h post-seeding, transfected with a DNA mix (6 µg of Gag/Pol, 3 µg of Rev, 1 µg of VSV-G; and, 6 µg of experimental plasmid (pLKO or pLKOshRNA-CBS/CTH) prepared in 500 μL of 0.1 mg/mL of PEI diluted in Opti-MEM GlutaMAX™ media (Gibco, cat. N° 51985034). The medium was replaced after overnight incubation with 10% FBS DMEM without antibiotics. Lentivirus-containing supernatant was harvested 48 h later, filtered by 0.45 μm syringe filters, concentrated by ultracentrifugation at 31,900 rpm 2 h at 4 °C and resuspended in sterile 1% w/v BSA in phosphate buffered saline (PBS). Virus aliquots were stored at −80 °C. Lentiviruses were quantified by Lenti-XTM p24 Rapid Titer Kit. To knock-down ECs, HUVECs were transduced with 100 MOI lentiviral particles containing pLKO empty plasmid (control) or pLKO plasmid containing shRNAs. Fresh media was replaced after 24 h. After 72 h post-infection the cells were recovered and silencing was assessed through western-blot and qPCR.

### Cell viability assay

Cells were tested for viability using crystal-violet staining as described by Feoktistova et al [56]. Briefly, after precoating with 0.2% w/v gelatin, HUVECs were seeded in 96-well plates (1 × 10^4^ cells/well) or in 3.5 cm dishes (2 × 10^5^ cells/dish). The day after cells were treated and 24 h post-treatment the medium was removed, the cells were washed in PBS and stained with 0.5% w/v crystal-violet (Sigma, cat. N° C0775) staining solution. After 20 min of incubation at room temperature on a bench rocker with a frequency of 20 oscillations per minute, the plates were washed with tap water. The residual dye in the air-dry plates was resuspended in 200 μL (96 well) or 1 mL (dish) of methanol. The optical density (OD) was measured using an Infinite M1000PRO Tecan microplate spectrophotometer at a wavelength of 590 nm.

### Cytometric analysis of lipid peroxidation

Lipid peroxidation was evaluated by flow cytometry using Bodipy-C11 581/591 (Thermo, cat. N° D3861). After 24 h of treatment, the cell medium was supplemented with 2.5 μM Bodipy-C11 for 20 min at 37 °C. Floating cells were collected and combined with adherent cells detached by trypsinization (Euro Clone, cat. N° ECB3052). Cellular pellets were washed in PBS and then resuspended in 300 μL. Flow cytometry analysis was performed using BD FACSCanto™ II Cell Analyzer (BD Biosciences). Non-treated cells without Bodipy-C11 staining were used as blank. The ratio between oxidized (FITC-A) and reduced (PE-A) Bodipy-C11 was measured. At least 20,000 events were evaluated per sample.

Cystine uptake experiments were carried out by incubating cells with 5 μM of FITC-labeled cystine (Millipore Sigma, cat. N° SCT047) for 30 min before collection. Living cells were washed in 1X HBSS, trypsinized, pelleted, and resuspended in 300 μL of 1X HBSS. The median fluorescence intensity (MFI) was measured for each sample using FITC-A filter and normalized to mean intensity in control.

### Cytometric analysis of cell death using propidium iodide

Cell death was analyzed by flow cytometry using propidium iodide staining. After the indicated treatments, floating cells were collected and combined with adherent cells detached by trypsinization (EuroClone, Cat. N° ECB3052). The cell pellets were washed with PBS, then resuspended in 300 µL of PBS. Propidium iodide (Sigma, cat. N° P4864) was added at a final concentration of 10 µg/mL prior to analysis. Flow cytometry was performed using the BD FACSCanto™ II Cell Analyzer (BD Biosciences).

### Real-time q-PCR

RNA isolation was performed with the TRIzol™ Reagent (Thermo, cat. N°.15596018) according to the manufacturer’s instructions. RNA concentration and purity were determined using the NanoDrop 1000 (Thermo Fisher Scientific) spectrophotometer. cDNA was synthesized from 1 μg of total RNA using High-Capacity cDNA Reverse Transcription Kit (Thermo, cat. N°. 4368814). For each gene of interest, the qPCR was run in triplicate using specific primers and 5x HOT FIREPol®EvaGreen® qRT-PCR Mix Plus (Solis BioDyne, cat. N°. 08-24-00001) on QuantStudio™ 5 (Applied Biosystems™). The data obtained were analysed using the 2-ΔΔCt method, as described by Schmittgen & Livak [57], normalized on the actin levels and graphed with GraphPad v.8.0.2.

The following human primers were used: SLC7A11-F: 5′-CCTCTATTCGGACCCATTTAGT-3′; SLC7A11-R: 5′-CTGGGTTTCTTGTCCCATATAA-3′, ACTIN-F: 5’-CCAACCGCGAGAAGATGA-3’; ACTIN-R: 5’-TCCATCACGATGCCAGTG-3’; HES-1-F: 5’-CGGACTCTAAACAGGAACTT-3’; HES-1-R: 5’-TACAAAGGCGCAATCCAATA-3’. GPX4-F: 5’-GCCTGGCCGGGACCAT-3’; GPX4-R: 5’-TCGATGTCCTTGGCGGAAAA-3’; FSP1-F: 5’-GGGGCTAGTAGTGGGGATAG-3’; FSP1-R: 5’-TCCTCATAGGCCTGGATAGC-3’; KLF2-F: 5’-GCACCGCCACTCACACCTG-3’; KLF2-R: 5’-CCGCAGCCGTCCCAGTTG-3’.

The following mouse primers were used: *Slc7A11*-F: 5′-TGGCTATCATCACAGTGGGC-3’; *Slc7A11*-R: 5′-GCAACAAAGATCGGGACTGC-3’; *Cbs*-F: 5’-TGGGAACACCCCTATGGTCA-3’; *Cbs*-R: 5’-CTGATGCGGTCCTTCACACT-3’; *Cth*-F: 5’-GATGGGGCAAAGCACAGTTT-3’; Cth-R: 5’-AATTCAGATGCCACCCTCCTG-3’; *Actin*-F: 5’-GTACTCTGTGTGGATCGGTGG-3’; *Actin*-R: 5’-AAACGCAGCTCAGTAACAGTCC-3’.

### Western blotting

Floating cells were collected and combined with adherent cells detached by scraping. Samples were washed twice on ice with cold PBS, and lysed in modified RIPA buffer (Thermo, cat. N° 89900) supplemented with protease and phosphatase inhibitor cocktail (Roche, cat. N° 04693116001 and 04906845001, respectively). Soluble lysates were clarified by centrifugation at 18,000 rpm for 10 min at 4 °C. Micro BCA assay (Pierce Biotechnology, cat. N° 23235) was used to determine protein concentration. A total of 10 μg of protein was loaded per well on 4-12% precast polyacrylamide gel (Thermo, cat. N°. NP0321BOX) and transferred to a nitrocellulose membrane (Amersham^TM^Protran^TM^, cat. N°. 10600015). After blocking with 5% milk in tris-buffered saline-tween (TBS-T) for 1 h, the membranes were incubated with primary antibodies overnight at 4 °C in 1% bovine serum albumin (BSA). The day after, the membranes were washed 3 times in TBS-T and incubated the appropriate horseradish peroxidase (HRP)-conjugated secondary antibodies (IgG) (1:10,000, Sigma). The signal was detected using Immobilon Forte Western HRP Substrate ECL (Millipore, cat. N°. WBLUF0500) and the images were acquired with the ChemiDoc MP system (Bio-Rad). The band intensities on developed films were quantified using ChemiDoc™ MP Image Analysis Software (Bio-Rad, USA).

The following antibodies were used: anti-SLC7A11 (1:1000; Novus Biological, cat. N° #NB300-318), anti-CBS (1:1000; Abcam, cat. N° ab140600), anti-CTH (1:1000; Proteintech, cat. N° 12217-1-AP), anti-GPX4 (1:2000; CST, cat. N° 52455), anti-FSP1 (1:1000; Santa Cruz Biotechnology, cat. N° sc-377120), anti-HO-1 (1:1000; Santa Cruz Biotechnology, cat. N° sc-10789), anti-NCID (1:1000; CST, cat. N° 4147S), anti-pHH3 (1:1000; Millipore, cat. N° 06-570), anti-CYCLIN A (1:1000; Sigma, cat. N° C4710), anti-HIF1alpha (1:1000; Genetex, cat. N° GTX127309), anti-ATF4 (1:1000; CST, cat. N° 11815), anti-p-P53 (1:1000; CST, cat. N° 9284), anti-P53 (1:500; Santa Cruz Biotechnology, cat. N° sc-126), anti-P-STAT3 (1:1000; CST, cat. N°9145), anti-STAT3 (1:1000; CST, cat. N° 9139), anti-NRF2 (1:1000, abcam, cat. N° ab76026), anti-ACTIN (1:2000; Sigma, cat. N° A5316), anti-VINCULIN (VCL) (1:1000; Sigma, cat. N° V9131), Goat anti-Rabbit IgG-HRP (1:10000; Sigma, cat. N° A6154), Goat anti-Mouse IgG-HRP (1:10000; Sigma, cat. N° A4416).

### Targeted metabolomics

The endothelial intracellular metabolites were extracted as previously reported by Vande Voorde et al. [58] HUVECs were plated in 10 cm pre-coated with 0.2% w/v gelatin dishes in complete M199 medium and let to reach 100% of confluence, then the medium was replaced with EGM2 and half of the plates were split 1:6 and re-plated in 10 cm dishes. After 48 h, the cells were treated with FMCys-Cys or control medium.

Metabolomic data were obtained using liquid chromatography coupled to tandem mass spectrometry. We used an API-3500 triplequadrupole mass spectrometer (AB Sciex, Framingham, MA, USA) coupled with an ExionLC™ AC System (AB Sciex). Cells were lysate in 250 µL ice-cold methanol/water/acetonitrile 55:25:20 containing 1 ng/µL [U-^13^C_6_]-D-glucose and 1 ng/µL [U-^13^C_5_]-L-glutamine as internal standards. Lysates were spun at 15,000 × *g* for 15 min at 4 °C. Samples were then dried under N_2_ flow at 40 °C and resuspended in 120 µL ice-cold MeOH/H_2_O 70:30 for subsequent analyses.

As previously described, quantification of amino acids, their derivatives, and biogenic amines was performed through previous derivatization [59]. Briefly, 10 µL of each sample were resuspended in 50 µL of 5% phenyl-isothiocyanate in EtOH, pyridine, and water (v/v, 1:1:1), then incubated for 20 min at RT, dried under N_2_ flow at 40 °C for 90 min, and finally resuspended in 100 µL 5 mM ammonium acetate in MeOH/H_2_O 50:50. Quantification of different amino acids was performed in positive ion mode, using a C18 column (Biocrates, Innsbruck, Austria) maintained at 50 °C. The mobile phases for the analysis were phase A: 0.2% formic acid in water and phase B: 0.2% formic acid in acetonitrile. The gradient was T0: 100% A, T5.5: 5% A, T7: 100% A with a flow rate of 500 µL/min.

The quantification of energy metabolites and cofactors was performed using a cyano-phase LUNA column (50 mm × 4.6 mm, 5 µm; Phenomenex) with a 5 min run in negative ion mode with two separated runs. Protocol A: mobile phase A was water and phase B was 2 mM ammonium acetate in MeOH, with a gradient of 10% A and 90% B for all analyses and a flow rate of 500 µL/min. Protocol B: mobile phase A was water and phase B was 2 mM ammonium acetate in MeOH, with a gradient of 50% A and 50% B for all analyses and a flow rate of 500 µL/min. Acylcarnitine quantification was performed on the same samples using a Varian Pursuit XRs Ultra 2.8 Diphenyl column (Agilent). Samples were analyzed in a 10 min run in positive ion mode. Mobile phases were A: 0.1% formic acid in H_2_O, B: 0.1%formic acid in MeOH, and the gradient was T0: 35% A, T2.0: 35% A, T5.0: 5% A, T5.5: 5% A, T5.51: 35% A, T9.0: 35% A with a flow rate of 300 µL/min. All metabolites were previously validated by pure standards and internal standards were used to check instrument sensitivity.

MultiQuant™ software (version 3.0.3, AB Sciex) was used for data analysis and peak review of chromatograms. Data processing and analysis were performed using the MetaboAnalyst 5.0 web tool [60]. Using MetaboAnalyst 5.0 for LC-HRMS spectra processing, multi-omics integration and covariate adjustment of global metabolomics data Nature Protocols [60], normalizing raw areas on median, transforming data in log_10_ scale and applying Pareto scaling. Obtained data were then compared to controls and expressed as fold change.

### Transsulfuration pathway metabolite analysis

As described above the cells were treated with FMCys-Cys or control medium. At recovery time, the cells were washed in PBS and scraped in 1 mL of ice-cold PBS. Part of the suspension (50 μL) was used to count the cells and measure protein concentration by BCA method. The remaining volume was centrifuged to remove the PBS and the dry cell pellet was stored at −80 °C. The protein concentration was then used to normalize the metabolite peak area value of transsulfuration pathway metabolites.

Cells were lysed in 500 µL of extraction solution (90% methanol with 0.03% (v/v) trifluoroacetic acid and 1.3 mM DTT), and then incubated for 10 min at RT, followed by centrifugation at 16,000 g for 10 min at 20°C. The supernatants were transferred to clean vials and dried under N_2_. The samples were reconstituted in 100 µL of ACN/H_2_O (v/v 90:10, 0.5% formic acid, 1 mM ammonium formate). Quantification of different metabolites was performed with a liquid chromatography/tandem mass spectrometry method using a Raptor Polar X (Restek, cat. N° 9311A12; 100 mm × 2.1 mm ID, 2.7 μm, 90 Å) column kept at 35 °C. Samples were analyzed by a 15 min run in positive ion mode with 13 multiple-reaction monitoring (MRM) transitions.

The mobile phases for the amino acid analysis were: phase A: 0.5% formic acid, 1 mM ammonium formate in water; and phase B: 0.5% formic acid in ACN/H_2_O (90:10 v/v), 1 mM ammonium formate. The gradient was: T0 4% A, T10 min 70% A, T10.1 min 95% A, T11.1 min 4% A, with a flow rate of 300 µL/min. Data were acquired on a Sciex Triple Quad 3500 (AB Sciex) with an HPLC system (AB Sciex) and a built-in autosampler (AB Sciex). MultiQuant software (version 3.0.2) was used for data analysis and peak review of chromatograms. The identification and quantification of the metabolites involved in the TSP was performed using standard curves generated with pure standards and using [U-^13^C_5_]-L-Methionine as internal standard (50 ng/sample).

### Retina angiogenesis study

For analysis of retinal angiogenesis, C57BL/6 pups at postnatal day 5 (P5) were administered intraperitoneally with 10 mg/kg of erastin (30 mg/kg high dose), 50 mg/kg of PAG and TFA. After 24 h, pups were sacrificed and whole mouse eyes were collected. The eyes were washed twice in cold PBS and fixed in 4% PFA on ice for 30 min. As Astone et al. described [61], eyes were washed in PBS, and the retinas were dissected and stored in methanol at −20 °C. Retinas were permeabilized in 1% BSA and 0.5% Triton X-100 in PBS at 4°C overnight. Retinas were rinsed in PBS, washed twice in PBLEC buffer (0.1 mM CaCl_2_, 0.1 mM MgCl_2_, 0.1 mM MnCl_2_ and 1% Triton X-100 in PBS), and incubated in 20 μg/mL isolectin GS-IB4 Alexa Fluor TM 488-conjugate (Thermo Fisher Scientific) for 4 h at 4°C. Retinas were washed five times for 20 minutes in PBS and left in PBS at 4°C overnight. After blocking in 2% goat serum, 1% BSA and 0.5% Triton X-100 (in PBS) for 1 h at room temperature, the retinas were incubated at 4 °C overnight in blocking buffer containing the ERG (1:100) primary antibody. After five washes with PBS, retinas were incubated with Alexa Fluor 568-conjugated secondary antibodies (1:500) in blocking buffer for 2 h at room temperature. Retinas were then washed four times for 20 min in PBS and partially cut into four leaflets. All quantifications were done on high-resolution confocal SP8 Leica images. EC area, vessel length and number of branching points were quantified using the Angiotool software [62]. All parameters were quantified in a minimum of two vascularized fields per sample.

Analysis of the retinal angiogenesis as described above was also performed in mice in which *Cth* gene was endothelial deleted by Cre-LoxP system previously reported by Xia et al [63]. Specifically, mice expressing tamoxifen-inducible Cre-recombinase (Cre-ERT2) under the regulation of vascular endothelial cadherin (*Cdh5*) promoter were injected intraperitoneally with 25 μl of 4-hydroxytamoxifen (4-OH-TAM) (4 mg/ml) at P1, P2, and P3 and retinas were harvested on P6. Control animals were littermates without CreERT2 expression. The deletion of *Cth* gene was confirmed by qPCR in ECs isolated from lungs as previously described [8].

All experiments conducted on the animals were following the Ethics Committee of the University of Padova and authorized by the Italian Ministry of Health (Permit Numbers: n. 398/2023-PR).

### Statistical analyses

GraphPad Prism v.8.0.2 was used to perform statistical analysis. The figures report the mean values ± SD calculated from at least three independent biological replicates (n). Statistical differences between two groups were evaluated by using two-tailed unpaired t-test or ordinary one-way ANOVA in the case of multiple comparisons. The statistical tests used for experiments are indicated in the corresponding figure legends. Statistical significance is reported as p-value.

## Supplementary information

Supplementary figures and legends

Uncropped gels

qPCR data

## Data Availability

The datasets generated during and/or analysed during the current study are available from the corresponding author on reasonable request.

## References

[1] Risau W, Flamme I. Vasculogenesis. Annu Rev Cell Dev Biol. 1995;11:73–91. 8689573 10.1146/annurev.cb.11.110195.000445

[2] Ehling M, Adams S, Benedito R, Adams RH. Notch controls retinal blood vessel maturation and quiescence. Development. 2013;140:3051–61. 23785053 10.1242/dev.093351

[3] Ricard N, Bailly S, Guignabert C, Simons M. The quiescent endothelium: signalling pathways regulating organ-specific endothelial normalcy. Nat Rev Cardiol. 2021;18:565–80. 33627876 10.1038/s41569-021-00517-4 PMC7903932

[4] Wälchli T, Ghobrial M, Schwab M, Takada S, Zhong H, Suntharalingham S, et al. Single-cell atlas of the human brain vasculature across development, adulthood and disease. Nature. 2024;632:603–13. 38987604 10.1038/s41586-024-07493-y PMC11324530

[5] Kalucka J, de Rooij LPMH, Goveia J, Rohlenova K, Dumas SJ, Meta E, et al. Single-cell transcriptome atlas of murine endothelial cells. Cell. 2020;180:764–79.e20. 32059779 10.1016/j.cell.2020.01.015

[6] Paik DT, Tian L, Williams IM, Rhee S, Zhang H, Liu C, et al. Single-cell RNA sequencing unveils unique transcriptomic signatures of organ-specific endothelial cells. Circulation. 2020;142:1848–62. 32929989 10.1161/CIRCULATIONAHA.119.041433 PMC7658053

[7] De Bock K, Georgiadou M, Schoors S, Kuchnio A, Wong BW, Cantelmo AR, et al. Role of PFKFB3-driven glycolysis in vessel sprouting. Cell. 2013;154:651–63. 23911327 10.1016/j.cell.2013.06.037

[8] Oberkersch RE, Pontarin G, Astone M, Spizzotin M, Arslanbaeva L, Tosi G, et al. Aspartate metabolism in endothelial cells activates the mTORC1 pathway to initiate translation during angiogenesis. Dev Cell. 2022;57:1241–56.e8. 35580611 10.1016/j.devcel.2022.04.018

[9] Kim B, Li J, Jang C, Arany Z. Glutamine fuels proliferation but not migration of endothelial cells. EMBO J. 2017;36:2321–33. 28659379 10.15252/embj.201796436 PMC5556269

[10] Kalucka J, Bierhansl L, Conchinha NV, Missiaen R, Elia I, Brüning U, et al. Quiescent endothelial cells upregulate fatty acid β-oxidation for vasculoprotection via redox homeostasis. Cell Metab. 2018;28:881–94.e13. 30146488 10.1016/j.cmet.2018.07.016

[11] Andrade J, Shi C, Costa ASH, Choi J, Kim J, Doddaballapur A, et al. Control of endothelial quiescence by FOXO-regulated metabolites. Nat Cell Biol. 2021;23:413–23. 33795871 10.1038/s41556-021-00637-6 PMC8032556

[12] Dixon SJ, Olzmann JA. The cell biology of ferroptosis. Nat Rev Mol Cell Biol. 2024;25:424–42. 38366038 10.1038/s41580-024-00703-5 PMC12187608

[13] Zheng J, Conrad M. The metabolic underpinnings of ferroptosis. Cell Metab. 2020;32:920–37. 33217331 10.1016/j.cmet.2020.10.011

[14] Combs JA, DeNicola GM. The non-essential amino acid cysteine becomes essential for tumor proliferation and survival. Cancers. 2019;11:678. 31100816 10.3390/cancers11050678 PMC6562400

[15] Sullivan MR, Danai LV, Lewis CA, Chan SH, Gui DY, Kunchok T, et al. Quantification of microenvironmental metabolites in murine cancers reveals determinants of tumor nutrient availability. Elife. 2019;8:e44235. 30990168 10.7554/eLife.44235 PMC6510537

[16] Zhu J, Berisa M, Schwörer S, Qin W, Cross JR, Thompson CB. Transsulfuration activity can support cell growth upon extracellular cysteine limitation. Cell Metab. 2019;30:865–876.e5. 31607565 10.1016/j.cmet.2019.09.009 PMC6961654

[17] Erdélyi K, Ditrói T, Johansson HJ, Czikora Á, Balog N, Silwal-Pandit L, et al. Reprogrammed transsulfuration promotes basal-like breast tumor progression via realigning cellular cysteine persulfidation. Proc Natl Acad Sci USA. 2021;118:e2100050118. 34737229 10.1073/pnas.2100050118 PMC8609449

[18] Meier M, Janosik M, Kery V, Kraus JP, Burkhard P. Structure of human cystathionine β-synthase: a unique pyridoxal 5′-phosphate-dependent heme protein. EMBO J. 2001;20:3910–6. 11483494 10.1093/emboj/20.15.3910 PMC149156

[19] Longchamp A, Mirabella T, Arduini A, MacArthur MR, Das A, Treviño-Villarreal JH, et al. Amino acid restriction triggers angiogenesis via GCN2/ATF4 regulation of VEGF and H2S production. Cell. 2018;173:117–29.e14. 29570992 10.1016/j.cell.2018.03.001 PMC5901681

[20] Lopes-Coelho F, Martins F, Hipólito A, Mendes C, Sequeira CO, Pires RF, et al. The activation of endothelial cells relies on a ferroptosis-like mechanism: novel perspectives in management of angiogenesis and cancer therapy. Front Oncol. 2021;11:656229. 34041026 10.3389/fonc.2021.656229 PMC8141735

[21] Han WM, Hong YX, Xiao GS, Wang RY, Li G. NMDARs activation regulates endothelial ferroptosis via the PP2A-AMPK-HMGB1 axis. Cell Death Discov. 2024;10:34. 38233385 10.1038/s41420-023-01794-3 PMC10794209

[22] Wakabayashi T, Naito H. Cellular heterogeneity and stem cells of vascular endothelial cells in blood vessel formation and homeostasis: Insights from single-cell RNA sequencing. Front Cell Dev Biol. 2023;11:1146399. 37025170 10.3389/fcell.2023.1146399 PMC10070846

[23] Zhong J, Gao RR, Zhang X, Yang JX, Liu Y, Ma J, et al. Dissecting endothelial cell heterogeneity with new tools. Cell Regen. 2025;14:10. 40121354 10.1186/s13619-025-00223-3 PMC11929667

[24] Du Y, Guo Z. Recent progress in ferroptosis: inducers and inhibitors. Cell Death Discov. 2022;8:501. 36581640 10.1038/s41420-022-01297-7 PMC9800531

[25] Dolma S, Lessnick SL, Hahn WC, Stockwell BR. Identification of genotype-selective antitumor agents using synthetic lethal chemical screening in engineered human tumor cells. Cancer Cell. 2003;3:285–96. 12676586 10.1016/s1535-6108(03)00050-3

[26] Gout PW, Buckley AR, Simms CR, Bruchovsky N. Sulfasalazine, a potent suppressor of lymphoma growth by inhibition of the x(c)- cystine transporter: a new action for an old drug. Leukemia. 2001;15:1633–40. 11587223 10.1038/sj.leu.2402238

[27] Poltorack CD, Dixon SJ. Understanding the role of cysteine in ferroptosis: progress & paradoxes. FEBS J. 2022;289:374–85. 33773039 10.1111/febs.15842 PMC8473584

[28] Dekker RJ, Boon RA, Rondaij MG, Kragt A, Volger OL, Elderkamp YW, et al. KLF2 provokes a gene expression pattern that establishes functional quiescent differentiation of the endothelium. Blood. 2006;107:4354–63. 16455954 10.1182/blood-2005-08-3465

[29] Santoro MM. The antioxidant role of non-mitochondrial CoQ10: mystery solved!. Cell Metab. 2020;31:13–5. 31951565 10.1016/j.cmet.2019.12.007

[30] Liu H, Forouhar F, Lin AJ, Wang Q, Polychronidou V, Soni RK, et al. Small-molecule allosteric inhibitors of GPX4. Cell Chem Biol. 2022;29:1680–93.e9. 36423641 10.1016/j.chembiol.2022.11.003 PMC9772252

[31] Asimakopoulou A, Panopoulos P, Chasapis CT, Coletta C, Zhou Z, Cirino G, et al. Selectivity of commonly used pharmacological inhibitors for cystathionine β synthase (CBS) and cystathionine γ lyase (CSE). Br J Pharm. 2013;169:922–32. 10.1111/bph.12171 PMC3687671 23488457

[32] Kabil O, Yadav V, Banerjee R. Heme-dependent Metabolite Switching Regulates H2S Synthesis in Response to Endoplasmic Reticulum (ER) Stress. J Biol Chem. 2016;291:16418–23. 27365395 10.1074/jbc.C116.742213 PMC4974357

[33] Pontes-Quero S, Fernández-Chacón M, Luo W, Lunella FF, Casquero-Garcia V, Garcia-Gonzalez I, et al. High mitogenic stimulation arrests angiogenesis. Nat Commun. 2019;10:2016. 31043605 10.1038/s41467-019-09875-7 PMC6494832

[34] Koziel A, Jarmuszkiewicz W. Hypoxia and aerobic metabolism adaptations of human endothelial cells. Pflug Arch. 2017;469:815–27. 10.1007/s00424-017-1935-9 PMC5438427 28176017

[35] Cohen EB, Geck RC, Toker A. Metabolic pathway alterations in microvascular endothelial cells in response to hypoxia. PLoS One. 2020;15:e0232072. 32645038 10.1371/journal.pone.0232072 PMC7347218

[36] He F, Zhang P, Liu J, Wang R, Kaufman RJ, Yaden BC, et al. ATF4 suppresses hepatocarcinogenesis by inducing SLC7A11 (xCT) to block stress-related ferroptosis. J Hepatol. 2023;79:362–77. 36996941 10.1016/j.jhep.2023.03.016 PMC11332364

[37] Jiang L, Kon N, Li T, Wang SJ, Su T, Hibshoosh H, et al. Ferroptosis as a p53-mediated activity during tumour suppression. Nature. 2015;520:57–62. 25799988 10.1038/nature14344 PMC4455927

[38] Linher-Melville K, Haftchenary S, Gunning P, Singh G. Signal transducer and activator of transcription 3 and 5 regulate system Xc- and redox balance in human breast cancer cells. Mol Cell Biochem. 2015;405:205–21. 25896132 10.1007/s11010-015-2412-4

[39] Fan Z, Wirth AK, Chen D, Wruck CJ, Rauh M, Buchfelder M, et al. Nrf2-Keap1 pathway promotes cell proliferation and diminishes ferroptosis. Oncogenesis. 2017;6:e371. 28805788 10.1038/oncsis.2017.65 PMC5608917

[40] Meydan N, Grunberger T, Dadi H, Shahar M, Arpaia E, Lapidot Z, et al. Inhibition of acute lymphoblastic leukaemia by a Jak-2 inhibitor. Nature. 1996;379:645–8. 8628398 10.1038/379645a0

[41] Singh A, Venkannagari S, Oh KH, Zhang YQ, Rohde JM, Liu L, et al. Small molecule inhibitor of NRF2 selectively intervenes therapeutic resistance in KEAP1-deficient NSCLC tumors. ACS Chem Biol. 2016;11:3214–25. 27552339 10.1021/acschembio.6b00651 PMC5367156

[42] Falkenberg KD, Rohlenova K, Luo Y, Carmeliet P. The metabolic engine of endothelial cells. Nat Metab. 2019;1:937–46. 32694836 10.1038/s42255-019-0117-9

[43] Li X, Sun X, Carmeliet P. Hallmarks of endothelial cell metabolism in health and disease. Cell Metab. 2019;30:414–33. 31484054 10.1016/j.cmet.2019.08.011

[44] Facchinello N, Astone M, Audano M, Oberkersch RE, Spizzotin M, Calura E, et al. Oxidative pentose phosphate pathway controls vascular mural cell coverage by regulating extracellular matrix composition. Nat Metab. 2022;4:123–40. 35102339 10.1038/s42255-021-00514-4 PMC7612297

[45] Liu Y, Wan Y, Jiang Y, Zhang L, Cheng W. GPX4: The hub of lipid oxidation, ferroptosis, disease and treatment. Biochim Biophys Acta Rev Cancer. 2023;1878:188890. 37001616 10.1016/j.bbcan.2023.188890

[46] Dixon SJ, Lemberg KM, Lamprecht MR, Skouta R, Zaitsev EM, Gleason CE, et al. Ferroptosis: an iron-dependent form of nonapoptotic cell death. Cell. 2012;149:1060–72. 22632970 10.1016/j.cell.2012.03.042 PMC3367386

[47] Soula M, Weber RA, Zilka O, Alwaseem H, La K, Yen F, et al. Metabolic determinants of cancer cell sensitivity to canonical ferroptosis inducers. Nat Chem Biol. 2020;16:1351–60. 32778843 10.1038/s41589-020-0613-y PMC8299533

[48] Bersuker K, Hendricks JM, Li Z, Magtanong L, Ford B, Tang PH, et al. The CoQ oxidoreductase FSP1 acts parallel to GPX4 to inhibit ferroptosis. Nature. 2019;575:688–92. 31634900 10.1038/s41586-019-1705-2 PMC6883167

[49] Friedmann Angeli JP, Conrad M. Selenium and GPX4, a vital symbiosis. Free Radic Biol Med. 2018;127:153–9. 29522794 10.1016/j.freeradbiomed.2018.03.001

[50] Mishima E, O’Neill TJ, Hoefig KP, Chen D, Behrens G, Henkelmann B, et al. MALT1 inhibitor MI-2 induces ferroptosis by direct targeting of GPX4. Proc Natl Acad Sci USA. 2025;122:e2507997122. 40343971 10.1073/pnas.2507997122 PMC12107143

[51] Sato H, Shiiya A, Kimata M, Maebara K, Tamba M, Sakakura Y, et al. Redox imbalance in cystine/glutamate transporter-deficient mice. J Biol Chem. 2005;280:37423–9. 16144837 10.1074/jbc.M506439200

[52] Armenta DA, Laqtom NN, Alchemy G, Dong W, Morrow D, Poltorack CD, et al. Ferroptosis inhibition by lysosome-dependent catabolism of extracellular protein. Cell Chem Biol. 2022;29:1588–1600.e7. 36306785 10.1016/j.chembiol.2022.10.006 PMC9762237

[53] Chen Y, Lu T, Liu Y, Liu Y, Bai S, Chen Q, et al. Establishment of SLC7A11-knockout mouse and its preliminary investigation in melanoma. Vitr Cell Dev Biol Anim. 2023;59:729–37. 10.1007/s11626-023-00819-6 37932516

[54] Zgorzynska E, Dziedzic B, Walczewska A. An overview of the Nrf2/ARE pathway and its role in neurodegenerative diseases. Int J Mol Sci. 2021;22:9592. 34502501 10.3390/ijms22179592 PMC8431732

[55] Liu N, Lin X, Huang C. Activation of the reverse transsulfuration pathway through NRF2/CBS confers erastin-induced ferroptosis resistance. Br J Cancer. 2020;122:279–92. 31819185 10.1038/s41416-019-0660-x PMC7052275

[56] Feoktistova M, Geserick P, Leverkus M. Crystal violet assay for determining viability of cultured cells. Cold Spring Harb Protoc. 2016;2016:pdb.prot087379. 27037069 10.1101/pdb.prot087379

[57] Schmittgen TD, Livak KJ. Analyzing real-time PCR data by the comparative C(T) method. Nat Protoc. 2008;3:1101–8. 18546601 10.1038/nprot.2008.73

[58] Vande Voorde J, Ackermann T, Pfetzer N, Sumpton D, Mackay G, Kalna G, et al. Improving the metabolic fidelity of cancer models with a physiological cell culture medium. Sci Adv. 2019;5:eaau7314. 30613774 10.1126/sciadv.aau7314 PMC6314821

[59] Audano M, Pedretti S, Cermenati G, Brioschi E, Diaferia GR, Ghisletti S, et al. Zc3h10 is a novel mitochondrial regulator. EMBO Rep. 2018;19:e45531. 29507079 10.15252/embr.201745531 PMC5891430

[60] Pang Z, Zhou G, Ewald J, Chang L, Hacariz O, Basu N, et al. Using MetaboAnalyst 5.0 for LC-HRMS spectra processing, multi-omics integration and covariate adjustment of global metabolomics data. Nat Protoc. 2022;17:1735–61. 35715522 10.1038/s41596-022-00710-w

[61] Astone M, Oberkersch RE, Tosi G, Biscontin A, Santoro MM. The circadian protein BMAL1 supports endothelial cell cycle during angiogenesis. Cardiovasc Res. 2023;119:1952–68. 37052172 10.1093/cvr/cvad057

[62] Zudaire E, Gambardella L, Kurcz C, Vermeren S. A computational tool for quantitative analysis of vascular networks. PLoS One. 2011;6:e27385. 22110636 10.1371/journal.pone.0027385 PMC3217985

[63] Xia H, Li Z, Sharp TE, Polhemus DJ, Carnal J, Moles KH, et al. Endothelial cell cystathionine γ-lyase expression level modulates exercise capacity, vascular function, and myocardial ischemia reperfusion injury. J Am Heart Assoc. 2020;9:e017544. 32990120 10.1161/JAHA.120.017544 PMC7792404

