# Supplementary material for: Transsulfuration metabolism is essential for ferroptosis resistance in quiescent endothelial cells
Source: Cell Death Dis. 2025 Dec 20;17(1):107. doi: 10.1038/s41419-025-08333-1 (PMC12847926; doi:10.1038/s41419-025-08333-1)
Supplement: Supplementary file 1 — Supplementary figures and legends [file 41419_2025_8333_MOESM1_ESM.pdf]

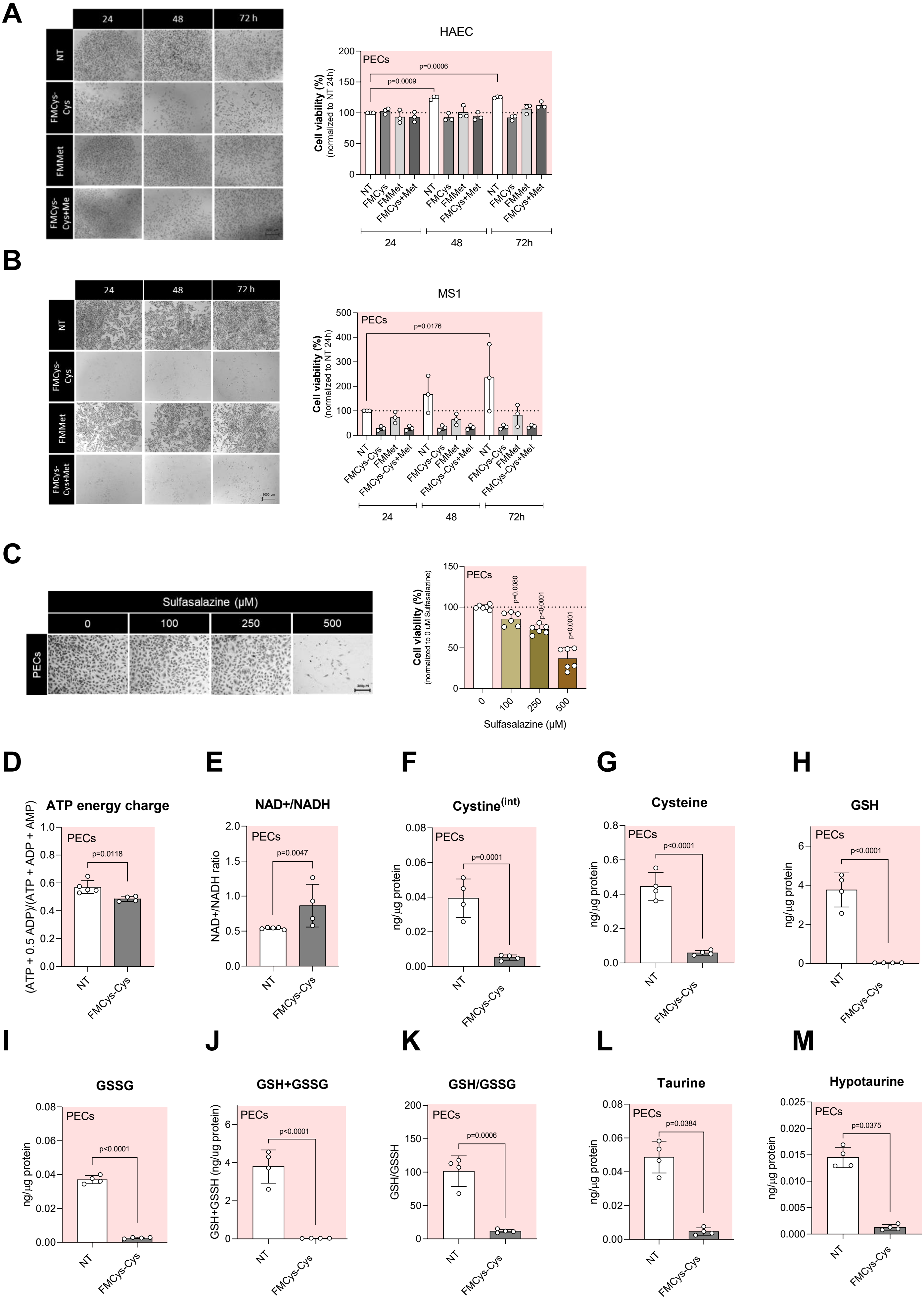
A
24
48
72 h
NT
FMCys-Cys
FMMet
FMCys-Cys+Me
HAEC
PECs
p=0.0006
p=0.0009
Cell viability (%)
(normalized to NT 24h)
NT
FMCys
FMMet
FMCys+Met
24
48
72h
B
MS1
p=0.0176
FMCys-Cys
FMCys-Cys+Met
C
Sulfasalazine (µM)
0
100
250
500
p=0.0080
p<0.0001
Cell viability (%)
(normalized to 0 uM Sulfasalazine)
D
ATP energy charge
p=0.0118
(ATP + 0.5 ADP)/(ATP + ADP + AMP)
E
NAD+/NADH
p=0.0047
NAD+/NADH ratio
F
Cystine(int)
p=0.0001
ng/µg protein
G
Cysteine
p<0.0001
H
GSH
I
GSSG
J
GSH+GSSG
GSH+GSSH (ng/ug protein)
K
GSH/GSSG
p=0.0006
GSH/GSSH
L
Taurine
p=0.0384
M
Hypotaurine
p=0.0375

Sup. Figure 1

**Sup. Figure 1. Metabolomics signature of PECs in Cystine deprivation conditions.**

(**A**) Cell viability assay using crystal violet staining on proliferating HAECs (PECs) grown in cystine-(FMCys-Cys), methionine- (FMMet) or both-(FMCys-Cys+Met) free medium for 24, 48 and 72h. The percentage of living cells was calculated using cells grown in 200 µM cystine and methionine 100 µM as a reference (NT). Statistical significance is given by one sample t-test and reported as a p-value in the figure.

(**B**) Cell viability assay using crystal violet staining on proliferating MS1 (PECs) grown in cystine-(FMCys-Cys), methionine- (FMMet) or both-(FMCys-Cys+Met) free medium for 24, 48 and 72h. The percentage of living cells was calculated using cells grown in 200 µM cystine and methionine 100 µM as a reference (NT). Statistical significance is given by one sample t-test and reported as a p-value in the figure.

(**C**) Cell viability assay using crystal violet staining on PECs treated with different concentrations (0; 100; 250; 500 µM) of sulfasalazine for 24h. At least 3 biological replicates were quantified and the percentage of living cells was calculated using 200 µM cystine (NT) condition as a reference. Statistical significance is given by one sample t-test and reported as a p-value in the figure.

(**D**) Adenylate energy charge (ATP+0.5XADP/(ATP+ADP+AMP)) in PECs grown in 200 µM cystine (NT) or FMCys-Cys medium for 24h measured by mass-spectrometry. Statistical significance is given by unpaired t-test and reported as adjusted p-value in the figure.

(**E**) NAD+/NADH ratio in PECs grown in 200 µM cystine (NT) or FMCys-Cys medium for 24h measured by mass-spectrometry. Statistical significance is given by unpaired t-test and reported as adjusted p-value in the figure.

Intracellular levels of cystine (**E**), cysteine (Cy) (**F**), reduced glutathione (GSH) (**G**), and oxidized glutathione (GSSG) (**H**) in proliferating ECs grown in 200 µM cystine (NT) or FMCys-Cys medium for 24h measured by mass-spectrometry. Statistical significance is given by unpaired t-test and reported as adjusted p-value in the figure.

(**I**) Total amount of glutathione (GSH+GSSG) in proliferating ECs grown in 200 µM cystine (NT) or FMCys-Cys medium for 24h measured by mass-spectrometry. Statistical significance is given by unpaired t-test and reported as adjusted p-value in the figure.

(**I**) Glutathione rate (GSH/GSSG) in proliferating ECs grown in 200 µM cystine (NT) or FMCys-Cys medium for 24h measured by mass-spectrometry. Statistical significance is given by unpaired t-test and reported as adjusted p-value in the figure.

Intracellular levels of taurine (**L**) and hypotaurine (**M**) in proliferating ECs grown in 200 µM cystine (NT) or FMCys-Cys medium for 24h measured by mass-spectrometry. Statistical significance is given by unpaired t-test and reported as adjusted p-value in the figure.

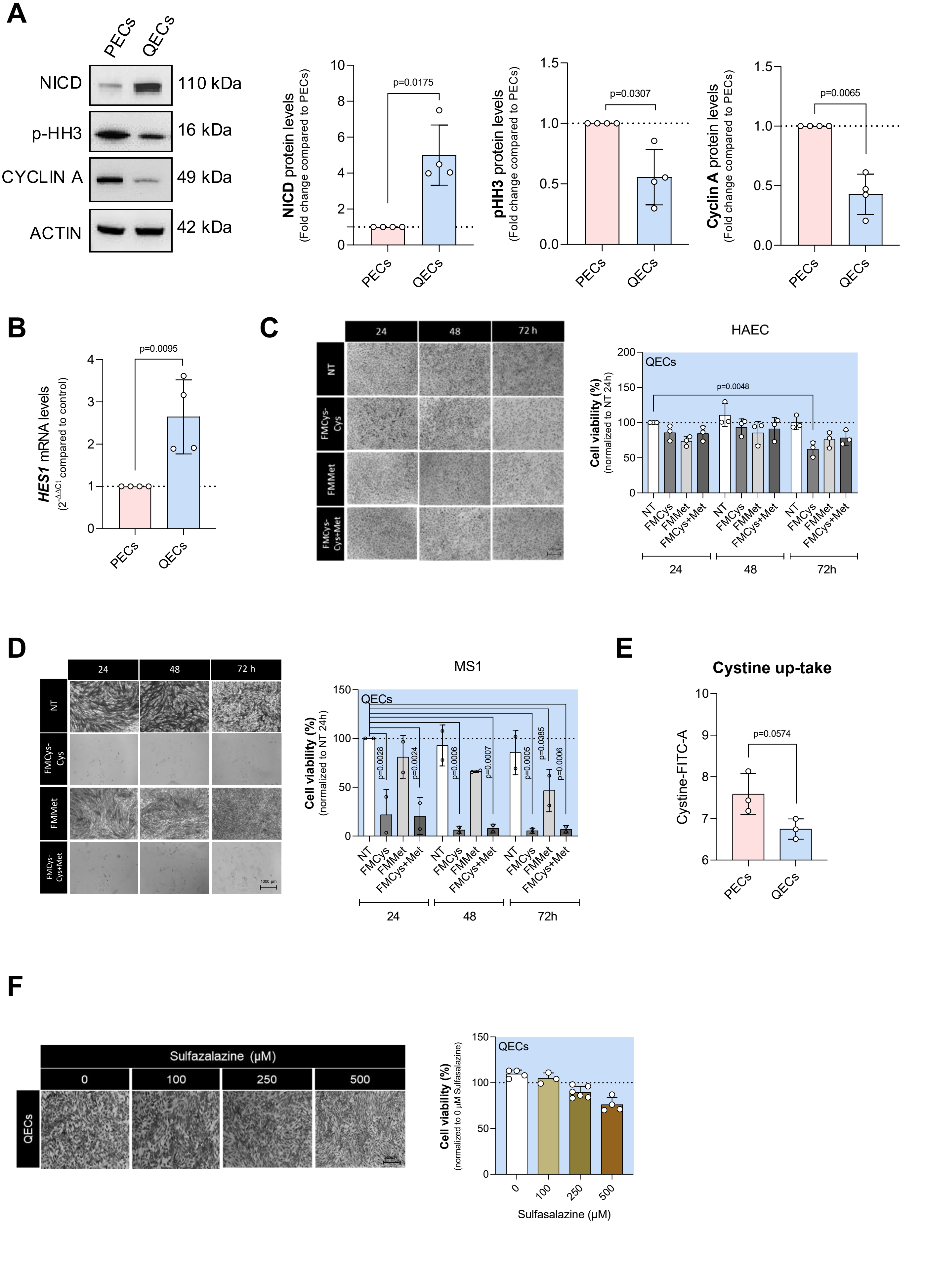
A
PECs
QECs
NICD
p-HH3
CYCLIN A
ACTIN
110 kDa
16 kDa
49 kDa
42 kDa
NICD protein levels (Fold change compared to PECs)
p=0.0175
pHH3 protein levels (Fold change compared to PECs)
p=0.0307
Cyclin A protein levels (Fold change compared to PECs)
p=0.0065
B
HES1 mRNA levels ($2^{-\Delta\Delta Ct}$ compared to control)
p=0.0095
C
24
48
72 h
NT
FMCys-Cys
FMMet
FMCys-Cys+Met
HAEC
QECs
Cell viability (%) (normalized to NT 24h)
p=0.0048
NT
FMCys
FMMet
FMCys+Met
24
48
72h
D
MS1
p=0.0028
p=0.0024
p=0.0006
p=0.0007
p=0.0005
p=0.0385
p=0.0006
1000 µm
E
Cystine up-take
Cystine-FITC-A
p=0.0574
F
Sulfazalazine (µM)
0
100
250
500
Cell viability (%) (normalized to 0 µM Sulfasalazine)
Sulfasalazine (µM)

Sup. Figure 2

**Sup. Figure 2. Quiescence of EC by contact inhibition.**

(**A**) Western blot analysis for cell cycle markers, including phosphohistone 3 (pHH3) (Ser10) and Cyclin A; and, NOTCH intracellular domain (NICD) in PECs versus QECs. ACTIN was used as loading control and band intensities were normalized on PECs. Statistical significance is given by one sample t-test and reported as adjusted p-value in the figure.

(**B**) Transcript levels of HES1 in PECs and QECs. ACTIN was used as control. Statistical significance is given by one sample t-test and reported as adjusted p-value in the figure.

(**C**) Cell viability assay using crystal violet staining on quiescent HAECs (QECs) grown in cystine-(FMCys-Cys), methionine- (FMMet) or both-(FMCys-Cys+Met) free medium for 24, 48 and 72h. The percentage of living cells was calculated using cells grown in 200 µM cystine and methionine 100 µM as a reference (NT). Statistical significance is given by one sample t-test and reported as a p-value in the figure.

(**D**) Cell viability assay using crystal violet staining on quiescent MS1 (QECs) grown in cystine-(FMCys-Cys), methionine- (FMMet) or both-(FMCys-Cys+Met) free medium for 24, 48 and 72h. The percentage of living cells was calculated using cells grown in 200 µM cystine and methionine 100 µM as a reference (NT). Statistical significance is given by one one-sample t-test and reported as a p-value in the figure.

(**E**) Uptake of FITC-labeled cystine by flow cytometry in PECs and QECs. Statistical significance is given by unpaired t-test and reported as adjusted p-value in the figure.

(**F**) Cell viability assay using crystal violet staining on QECs treated with increasing concentration (0; 100; 250; 500 µM) of sulfasalazine for 24h. At least 3 biological replicates were quantified and the percentage of living cells was calculated using untreated condition as a reference. Statistical significance is given by one sample t-test and reported as a p-value in the figure.

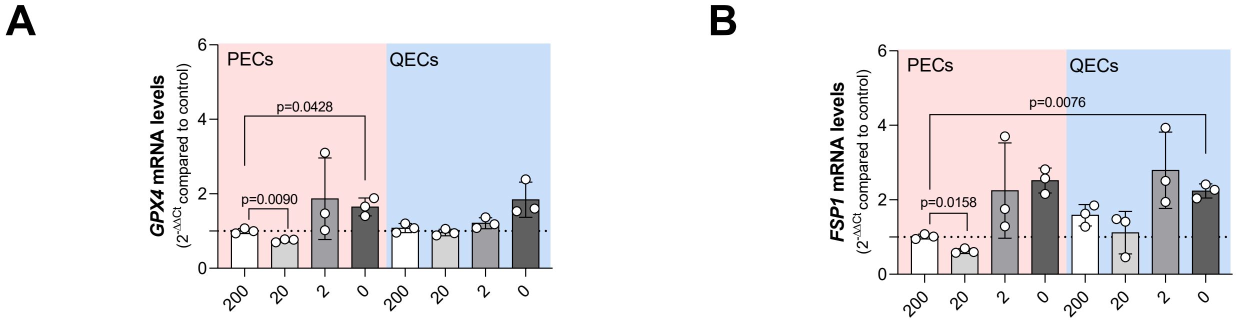


**Sup. Figure 3. Modulation of GPX4 and FSP1 mRNA expression in PECs vs QECs under decreasing cystine concentrations.**

The GPX4 (**A**) and FSP1 (**B**) mRNA levels of PECs and QECs under decreasing concentration of cystine for 24h. ACTIN was used as control. Statistical significance is given by one sample t-test and reported as a p-value in the figure.

**Sup. Figure 3**

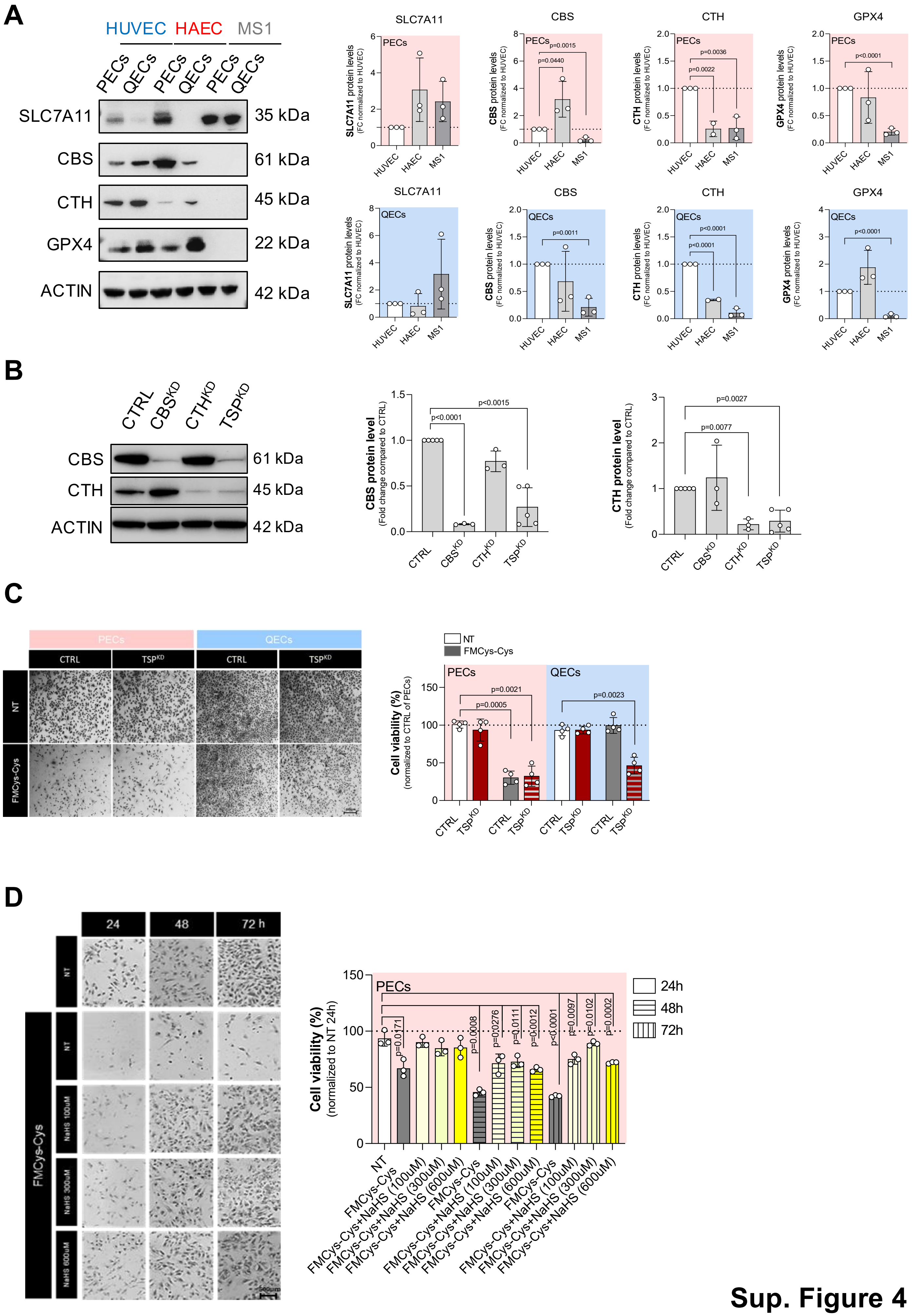
A
HUVEC HAEC MS1
PECs QECs PECs QECs PECs QECs
SLC7A11 35 kDa
CBS 61 kDa
CTH 45 kDa
GPX4 22 kDa
ACTIN 42 kDa
SLC7A11
CBS
CTH
GPX4
PECs
QECs
SLC7A11 protein levels (FC normalized to HUVEC)
CBS protein levels (FC normalized to HUVEC)
CTH protein levels (FC normalized to HUVEC)
GPX4 protein levels (FC normalized to HUVEC)
HUVEC HAEC MS1
p=0.0015
p=0.0440
p=0.0036
p=0.0022
p<0.0001
p=0.0011
p<0.0001
p<0.0001
p<0.0001
B
CTRL CBSKD CTHKD TSPKD
CBS 61 kDa
CTH 45 kDa
ACTIN 42 kDa
CBS protein level (Fold change compared to CTRL)
CTH protein level (Fold change compared to CTRL)
p<0.0001
p<0.0015
p=0.0027
p=0.0077
C
PECs QECs
CTRL TSPKD CTRL TSPKD
NT
FMCys-Cys
NT
FMCys-Cys
Cell viability (%) (normalized to CTRL of PECs)
p=0.0021
p=0.0005
p=0.0023
D
24 48 72 h
NT
FMCys-Cys
NaHS 100uM
NaHS 300uM
NaHS 600uM
PECs
24h
48h
72h
Cell viability (%) (normalized to NT 24h)
p=0.0171
p=0.0008
p=0.0276
p=0.0111
p=0.0012
p<0.0001
p=0.0097
p=0.0102
p=0.0002
NT
FMCys-Cys
FMCys-Cys+NaHS (100uM)
FMCys-Cys+NaHS (300uM)
FMCys-Cys+NaHS (600uM)

**Sup. Figure 4**

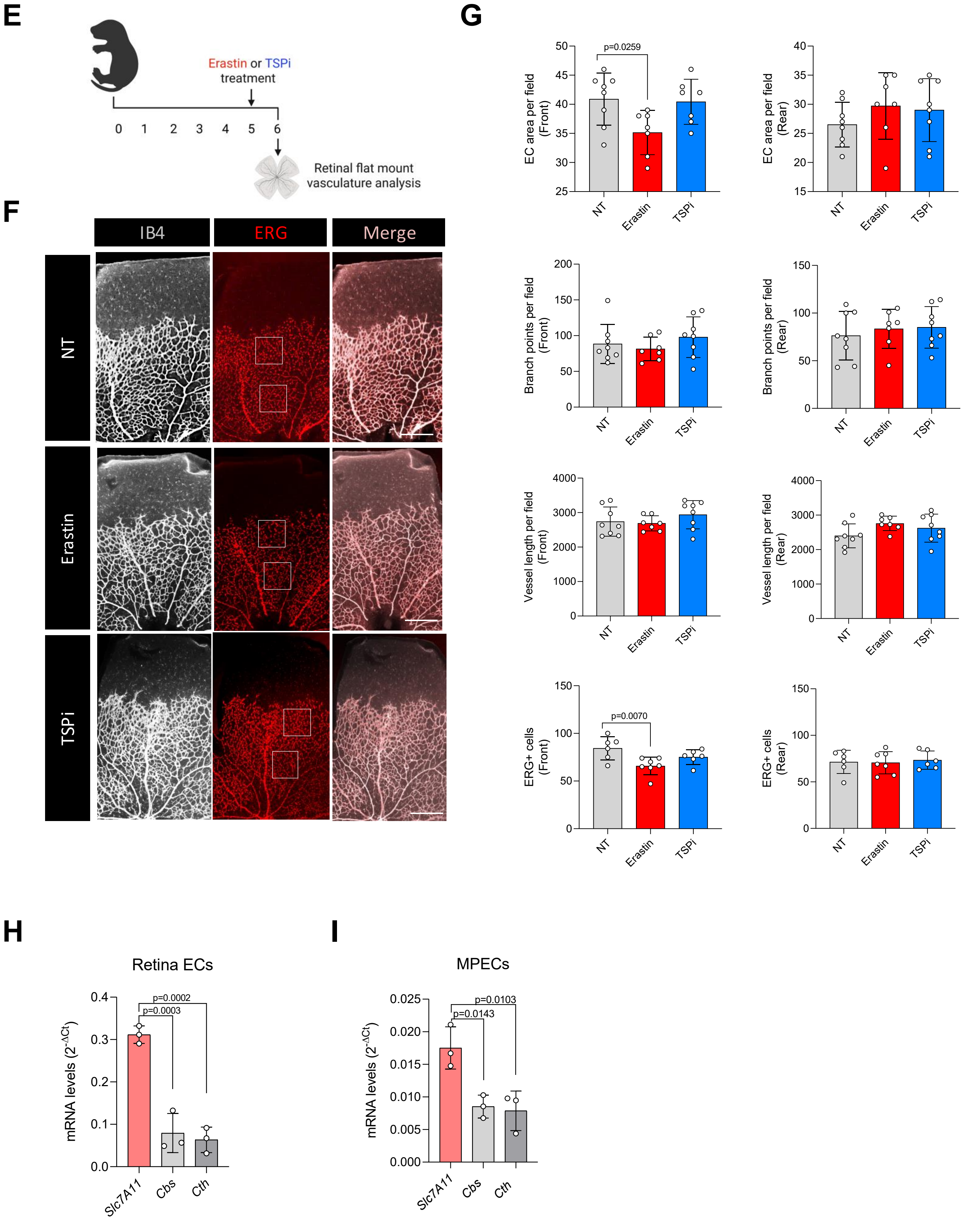

E
Erastin or TSPi
treatment
0 1 2 3 4 5 6
Retinal flat mount
vasculature analysis
F
IB4
ERG
Merge
NT
Erastin
TSPi
G
EC area per field (Front)
p=0.0259
EC area per field (Rear)
Branch points per field (Front)
Branch points per field (Rear)
Vessel length per field (Front)
Vessel length per field (Rear)
ERG+ cells (Front)
p=0.0070
ERG+ cells (Rear)
NT
Erastin
TSPi
H
Retina ECs
p=0.0002
p=0.0003
mRNA levels (2^-ΔCt)
Slc7A11
Cbs
Cth
I
MPECs
p=0.0103
p=0.0143
mRNA levels (2^-ΔCt)
Slc7A11
Cbs
Cth


**Sup. Figure 4**

J

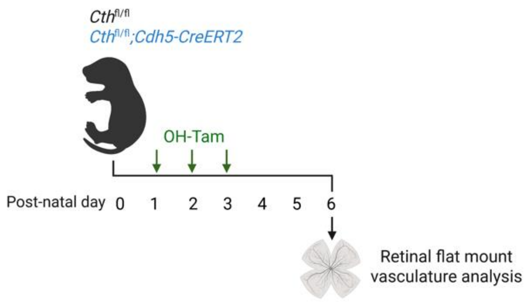


K

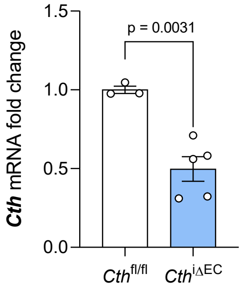


L

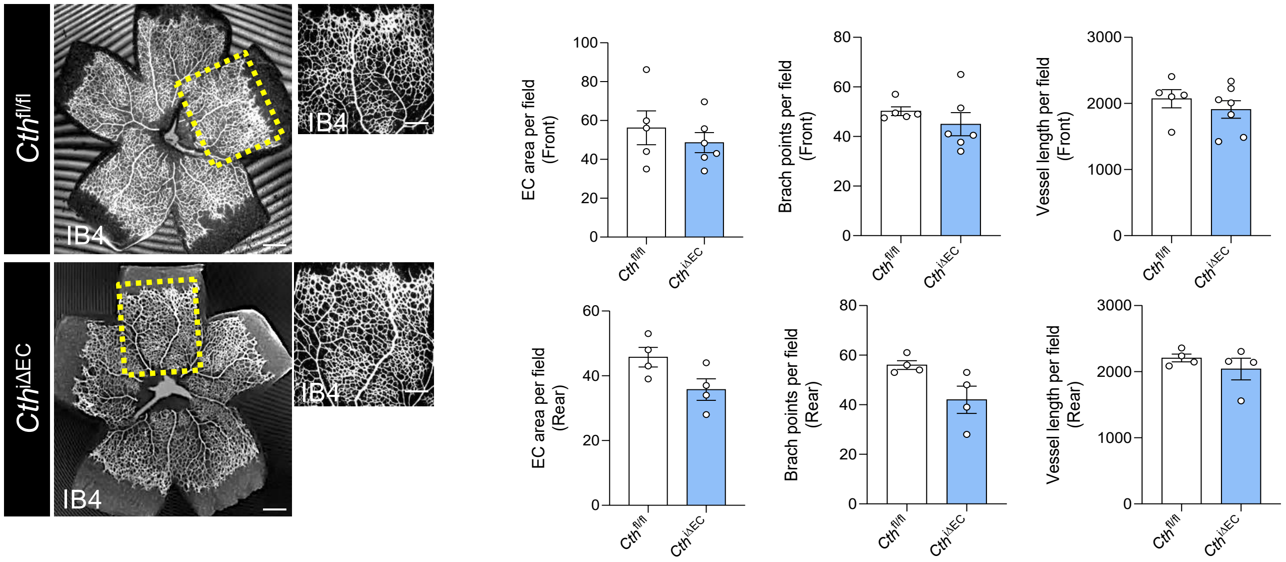


**Sup. Figure 4**

**Sup. Figure 4. TSP-derived $H_2S$ rescues ferroptosis in PECs under cystine starvation and sprouting angiogenesis *in vivo*, relying on extracellular cysteine.**

(**A**) Western blot analysis for SLC7A11, CBS, CTH and GPX4 in proliferating and quiescent endothelial cell lines (HUVEC, HAEC and MS1). ACTIN was used as loading control and band intensities were normalized on HUVEC (control). Statistical significance is given by one sample t-test and reported as a p-value in the figure.

(**B**) Western blot analysis for CBS and CTH in HUVECs knocked down for both TSP enzymes (shCBS + shCTH). ACTIN was used as loading control and band intensities were normalized on control condition. Statistical significance is given by one sample t-test and reported as a p-value in the figure.

(**C**) Cell viability assay using crystal violet staining on PECs and QECs knocked down for both TSP enzymes (CBS + CTH) and grown in control medium (200 µM cystine) or FMCys-Cys for 24h. Statistical significance is given by one sample t-test and reported as a p-value in the figure.

(**D**) Cell viability assay using crystal violet staining on PECs grown over 5 days in FMCys-Cys and treated with increasing concentrations (100; 300; 600 µM) of NaHS. Statistical significance is given by one sample t-test and reported as a p-value in the figure.

(**E**) Schematic representation of the treatment protocol used to evaluate the role of TSPi and erastin in a retinal angiogenesis model.

(**F**) Representative confocal images of isolectin B4-stained (IB4) and ERG of postnatal P6 mouse retinas, either untreated or treated with erastin or TSPi. Scale bar 150 µm.

(**G**) Quantification of different angiogenic parameters of retinas NT, or treated with erastin, or TSPi. N ≥ 7 biologically independent samples, pooled across at least three independent experiments. Data are shown as mean ± SEM. Statistics were done using one-way ANOVA, Dunnett's test.

Analysis of mRNA levels of *Scl7a1*11, *Cbs*, and *Cth* of retina ECs (**H**) and micropulmonary ECs (MPECs) (**I**) isolated from mouse retinas and lungs, respectively, at P6. Statistical significance is given by unpaired t-test and reported as adjusted p-value in the figure.

(**J**) Diagram illustrating the experimental procedure used to analyse retinal angiogenesis in $Cth^{fl/fl}$ and $Cth^{fl/fl}$; Cdh5-CreERT2 ($Cth^{i\Delta EC}$) mice.

(**K)** Analysis of *Cth* mRNA levels at postnatal day 6 in pulmonary microvascular ECs isolated from lung tissue.

**(L)** Representative images of IB4 staining in retinal flat-mount vasculature, along with corresponding quantification. N ≥ 5 mice per group.

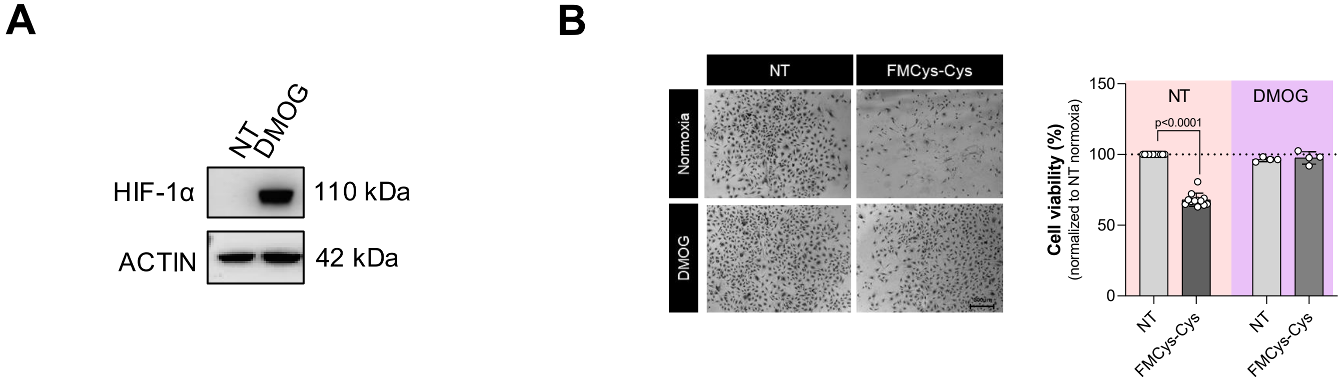


**Sup. Figure 5. DMOG-induced hypoxic PECs resist ferroptosis under cystine limitation.**

(**A**) Western blot analysis for HIF-1α in HUVECs exposed to DMOG 1 mM for 48h. ACTIN was used as loading control.

(**B**) Cell viability assay using crystal violet staining on PECs untreated or treated with DMOG 1mM for 48h, incubated in control medium (200 µM cystine) or FMCys-Cys for 24h. Statistical significance is given by one sample t-test and reported as a p-value in the figure.

**Sup. Figure 5**
