# Supplementary material for: Transsulfuration metabolism is essential for ferroptosis resistance in quiescent endothelial cells
Source: Cell Death Dis. 2025 Dec 20;17(1):107. doi: 10.1038/s41419-025-08333-1 (PMC12847926; doi:10.1038/s41419-025-08333-1)
Supplement: Supplementary file 2 — Uncropped gels [file 41419_2025_8333_MOESM2_ESM.pdf]

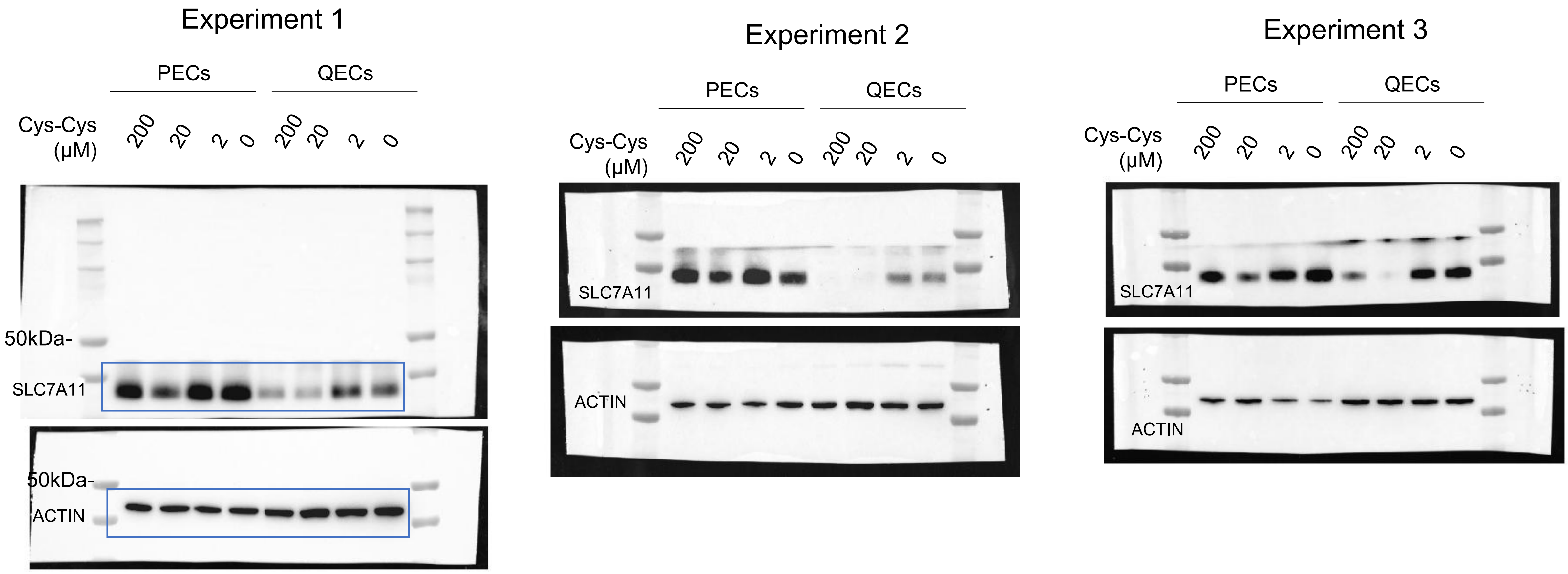

Figure 2F

The protein ladder utilized is Precision Plus Protein Dual Color Standards, #1610374, Biorad.

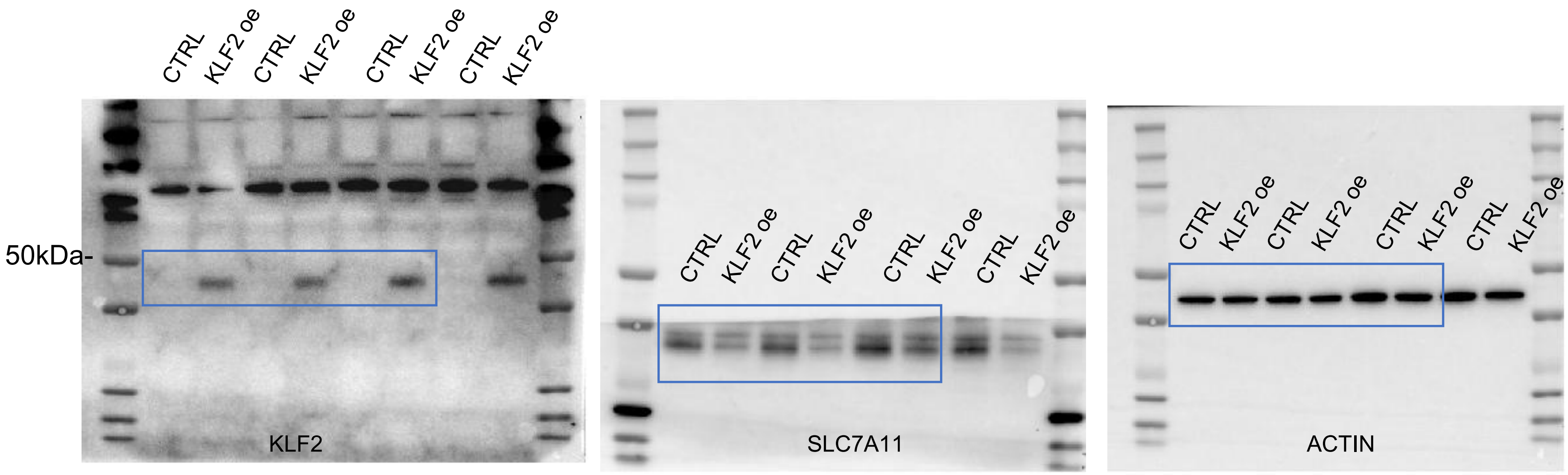
CTRL
KLF2 oe
CTRL
KLF2 oe
CTRL
KLF2 oe
CTRL
KLF2 oe
50kDa-
KLF2
CTRL
KLF2 oe
CTRL
KLF2 oe
CTRL
KLF2 oe
CTRL
KLF2 oe
SLC7A11
CTRL
KLF2 oe
CTRL
KLF2 oe
CTRL
KLF2 oe
CTRL
KLF2 oe
ACTIN

Figure 2K

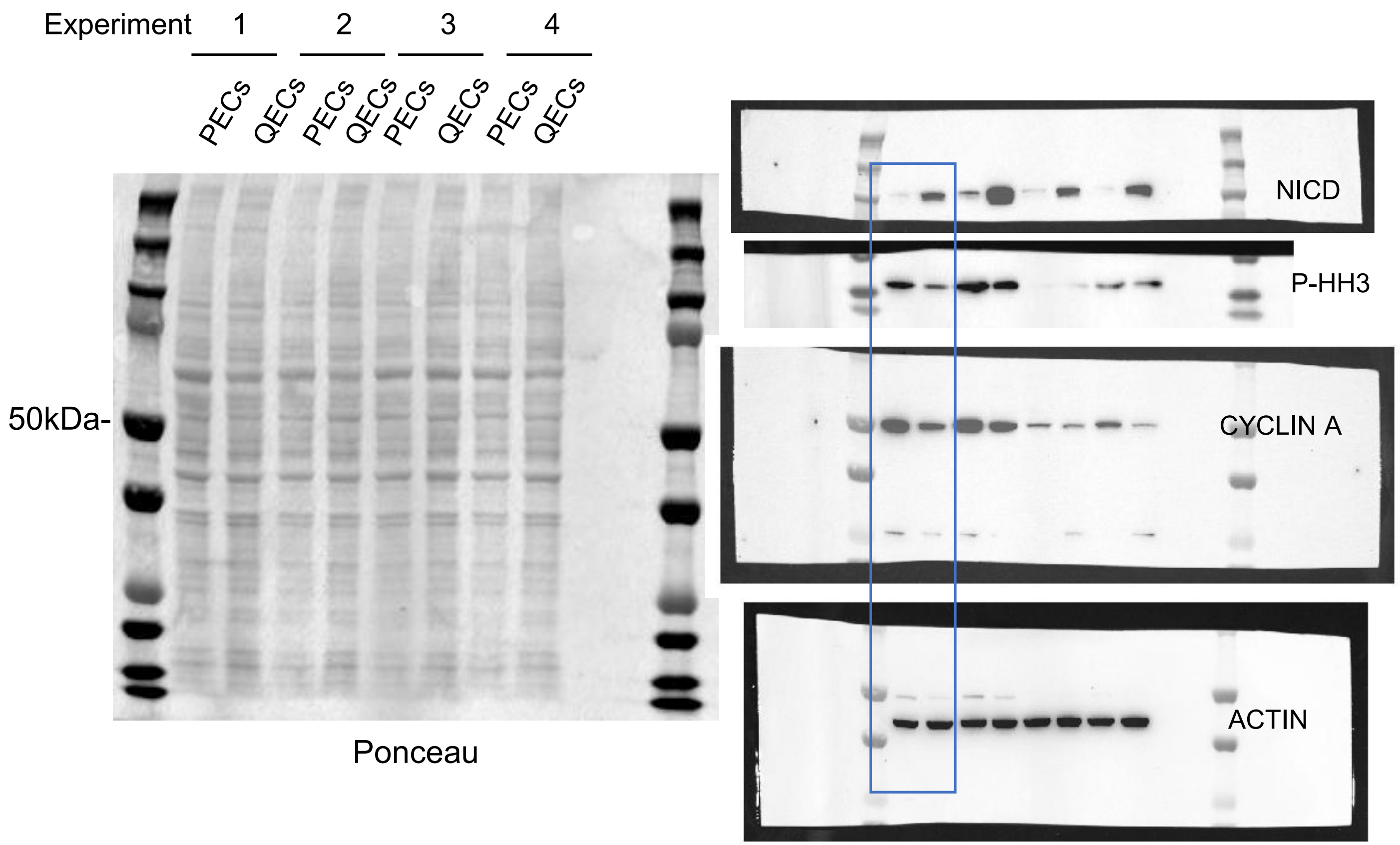

Sup. Figure 2A

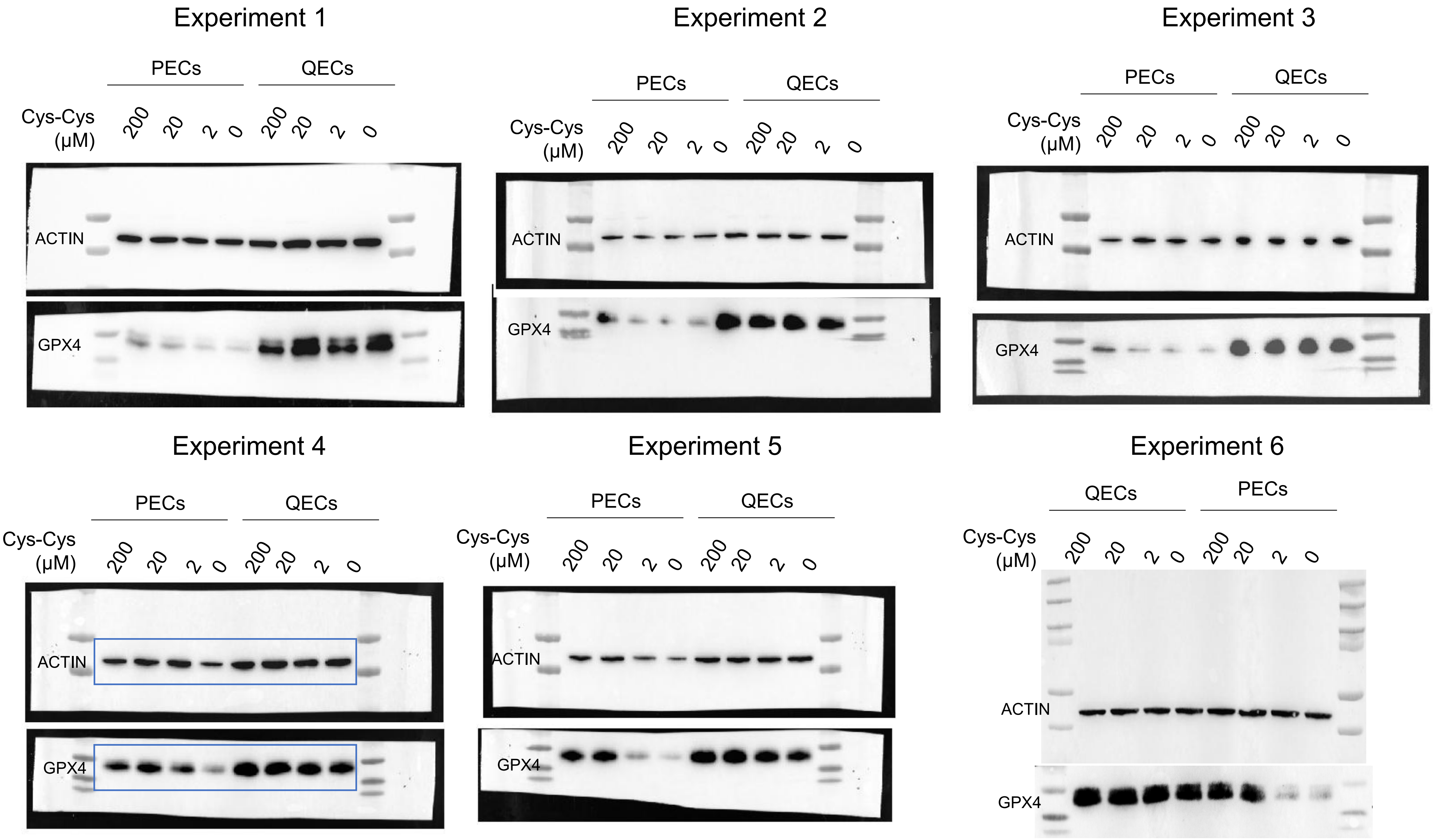
Experiment 1
PECs
QECs
Cys-Cys (μM)
200 20 2 0 200 20 2 0
ACTIN
GPX4
Experiment 2
PECs
QECs
Cys-Cys (μM)
200 20 2 0 200 20 2 0
ACTIN
GPX4
Experiment 3
PECs
QECs
Cys-Cys (μM)
200 20 2 0 200 20 2 0
ACTIN
GPX4
Experiment 4
PECs
QECs
Cys-Cys (μM)
200 20 2 0 200 20 2 0
ACTIN
GPX4
Experiment 5
PECs
QECs
Cys-Cys (μM)
200 20 2 0 200 20 2 0
ACTIN
GPX4
Experiment 6
QECs
PECs
Cys-Cys (μM)
200 20 2 0 200 20 2 0
ACTIN
GPX4

Figure 3A

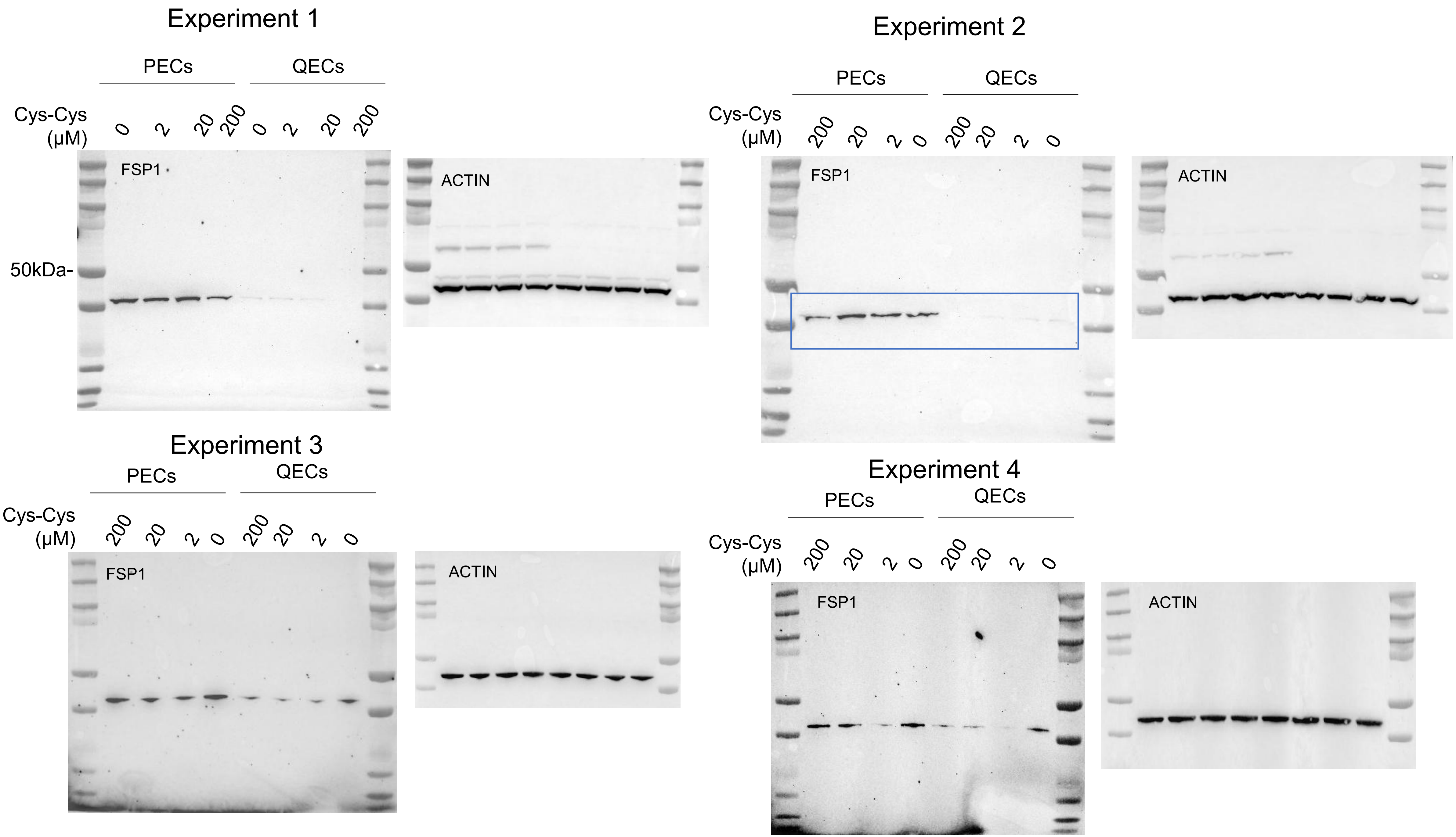
Experiment 1
PECs
QECs
Cys-Cys (μM)
0 2 20 200 0 2 20 200
FSP1
50kDa-
ACTIN
Experiment 2
PECs
QECs
Cys-Cys (μM)
200 20 2 0 200 20 2 0
FSP1
ACTIN
Experiment 3
PECs
QECs
Cys-Cys (μM)
200 20 2 0 200 20 2 0
FSP1
ACTIN
Experiment 4
PECs
QECs
Cys-Cys (μM)
200 20 2 0 200 20 2 0
FSP1
ACTIN

Figure 3A

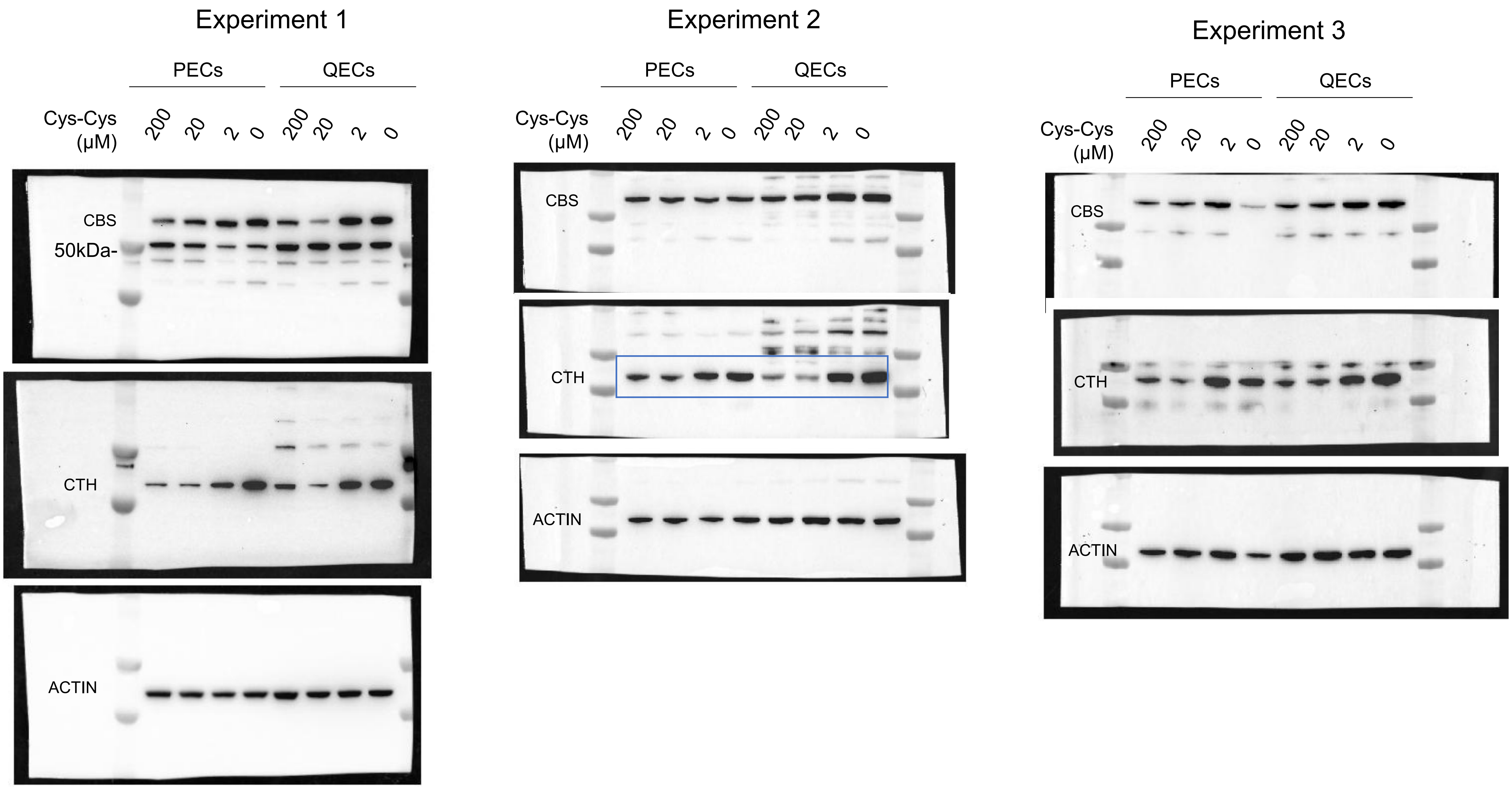
Experiment 1
PECs
QECs
Cys-Cys (μM)
200 20 2 0 200 20 2 0
CBS
50kDa-
CTH
ACTIN
Experiment 2
PECs
QECs
Cys-Cys (μM)
200 20 2 0 200 20 2 0
CBS
CTH
ACTIN
Experiment 3
PECs
QECs
Cys-Cys (μM)
200 20 2 0 200 20 2 0
CBS
CTH
ACTIN

Figure 4A

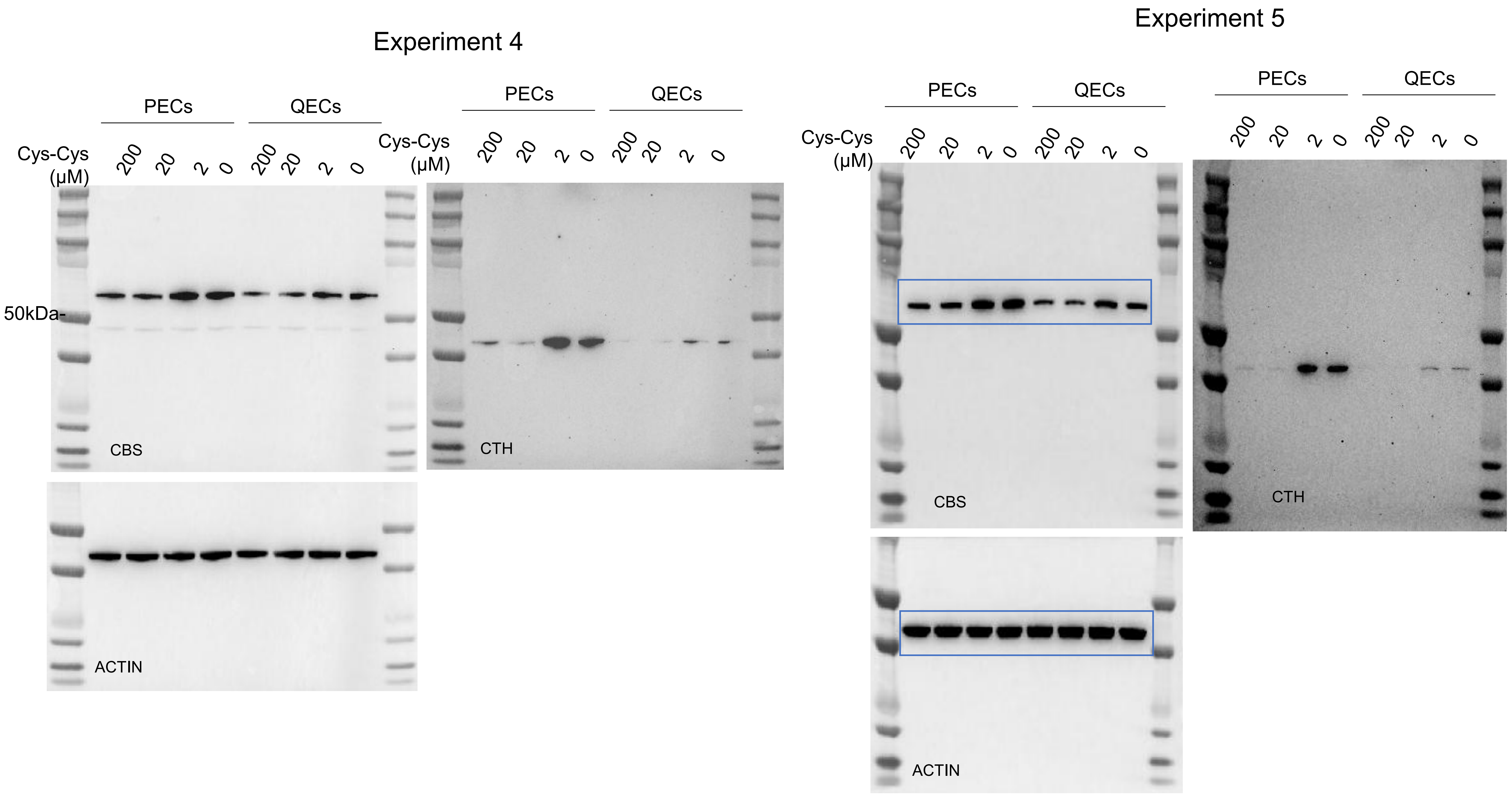
Experiment 4
PECs
QECs
Cys-Cys (μM)
200
20
2
0
200
20
2
0
50kDa-
CBS
ACTIN
PECs
QECs
Cys-Cys (μM)
200
20
2
0
200
20
2
0
CTH
Experiment 5
PECs
QECs
Cys-Cys (μM)
200
20
2
0
200
20
2
0
CBS
ACTIN
PECs
QECs
200
20
2
0
200
20
2
0
CTH

Figure 4A

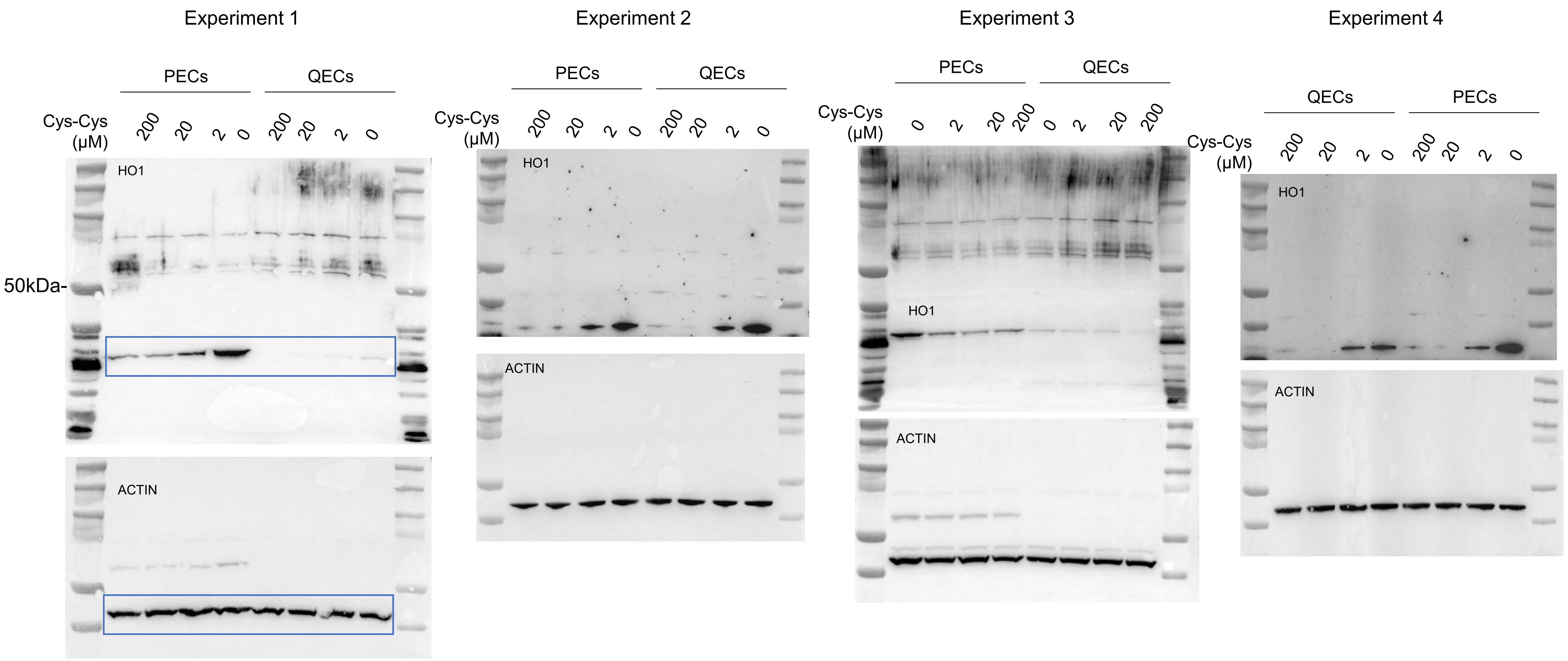
Experiment 1
PECs
QECs
Cys-Cys (µM)
200 20 2 0 200 20 2 0
HO1
50kDa-
ACTIN
Experiment 2
PECs
QECs
Cys-Cys (µM)
200 20 2 0 200 20 2 0
HO1
ACTIN
Experiment 3
PECs
QECs
Cys-Cys (µM)
0 2 20 200 0 2 20 200
HO1
ACTIN
Experiment 4
QECs
PECs
Cys-Cys (µM)
200 20 2 0 200 20 2 0
HO1
ACTIN

Figure 4G

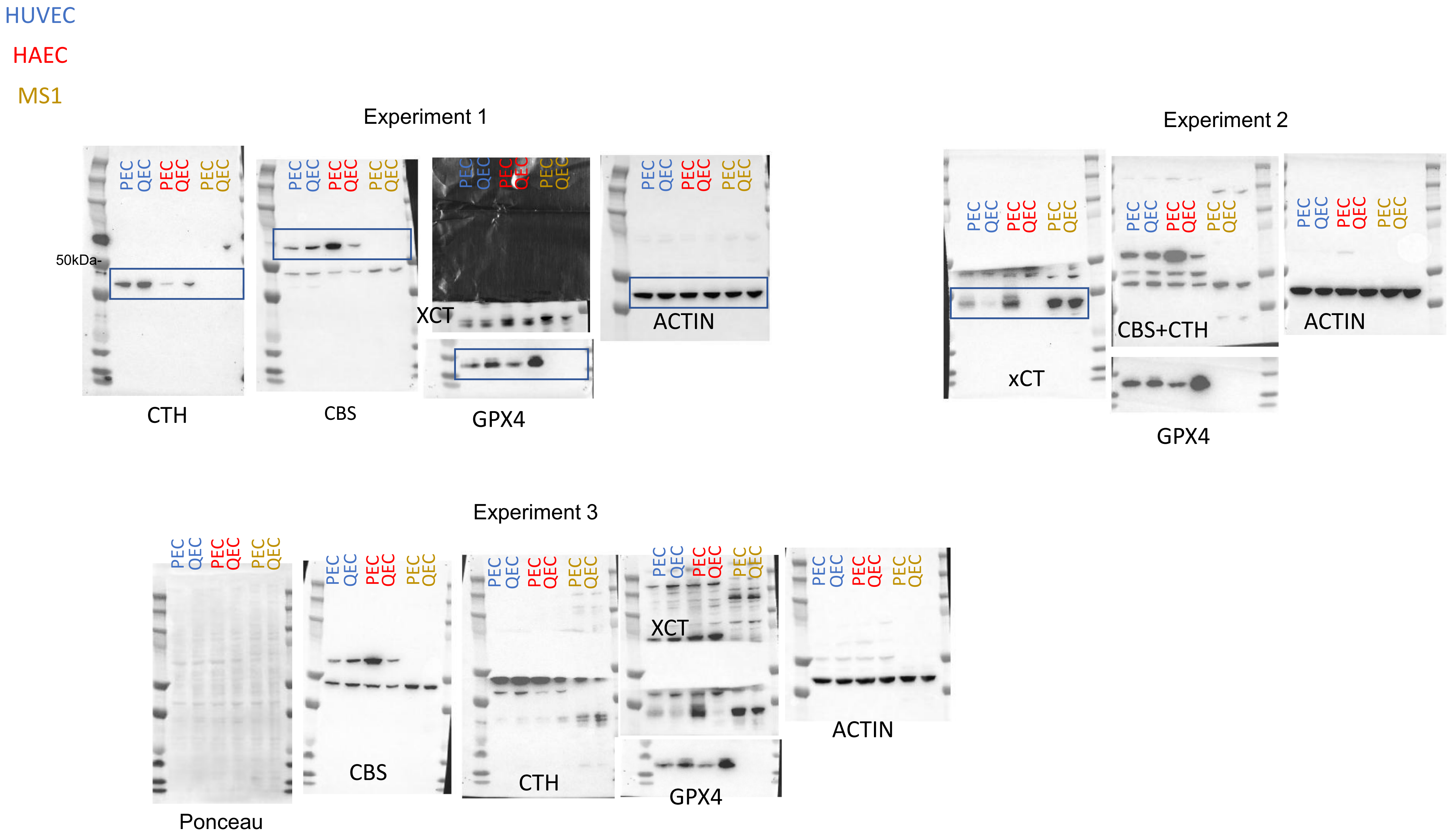

Sup. Figure 4A

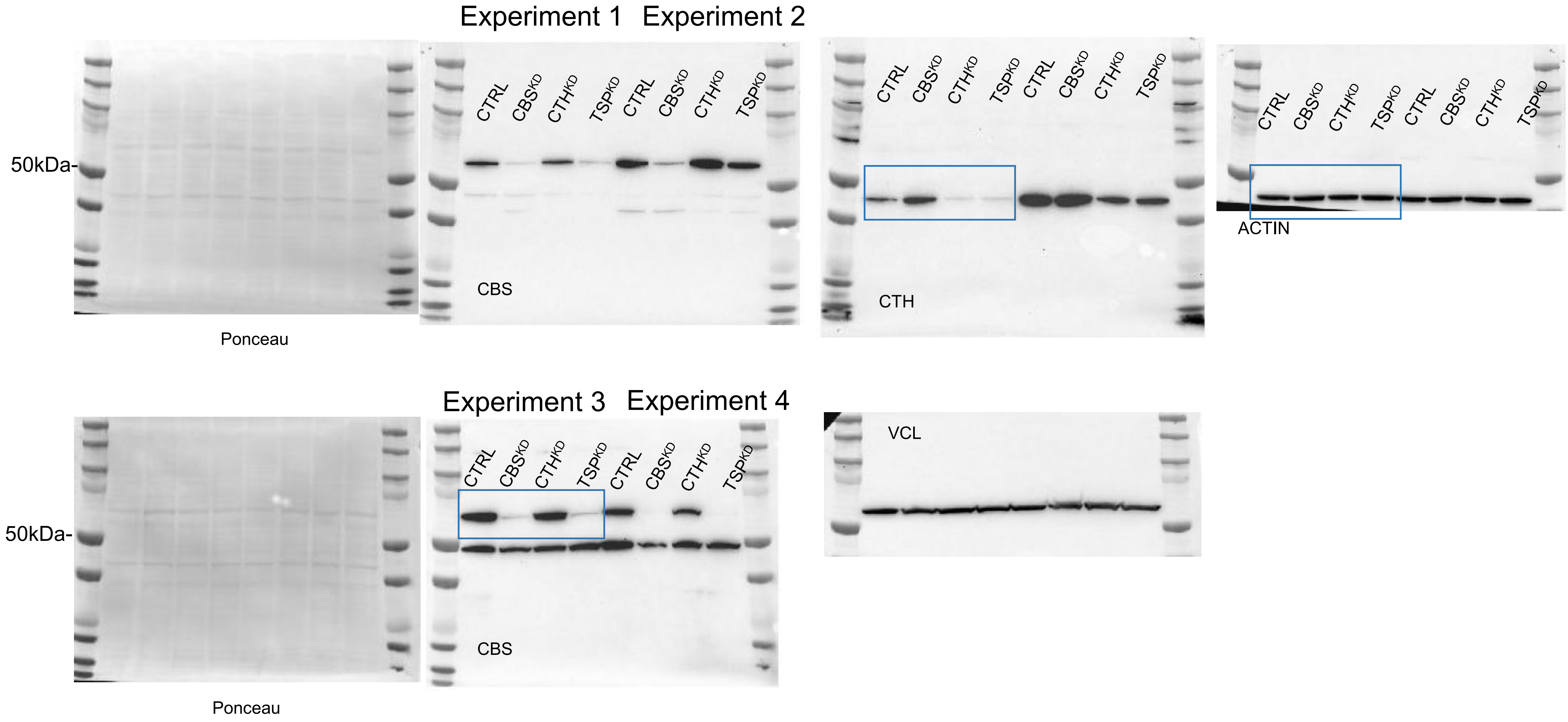

Sup. Figure 4B

Experiment 5

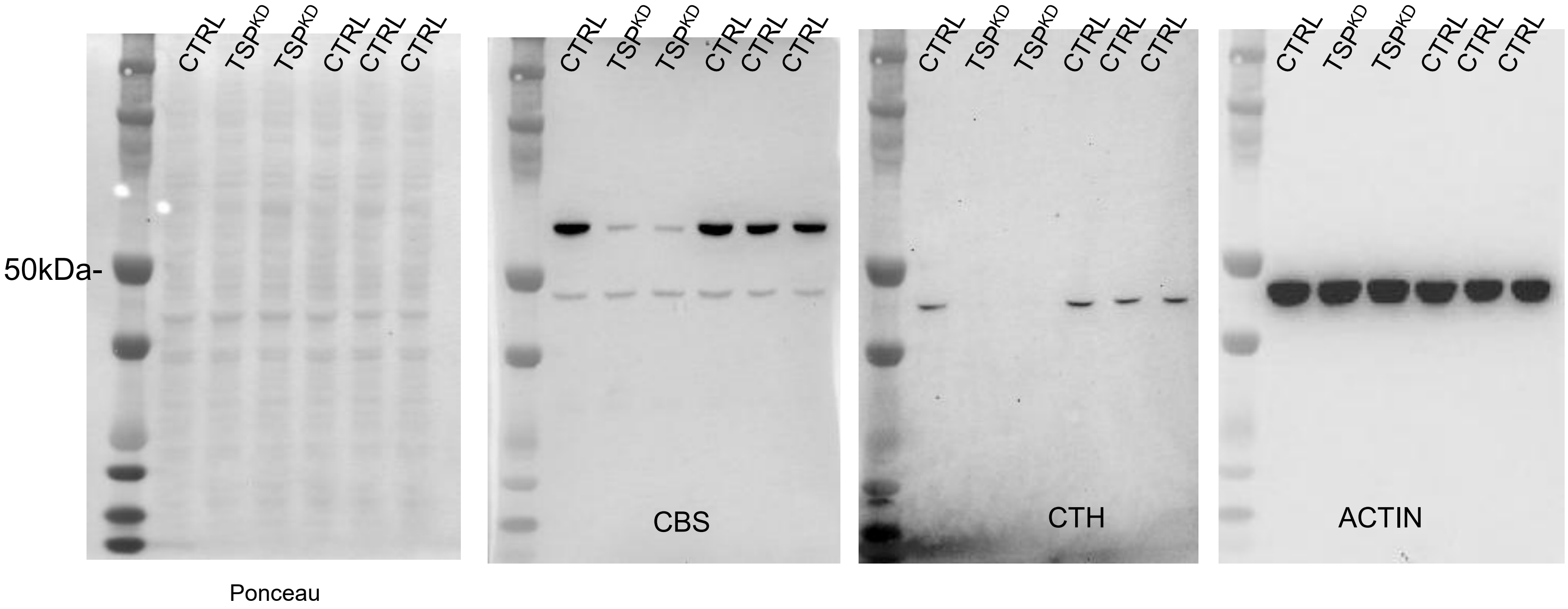

Sup. Figure 4B

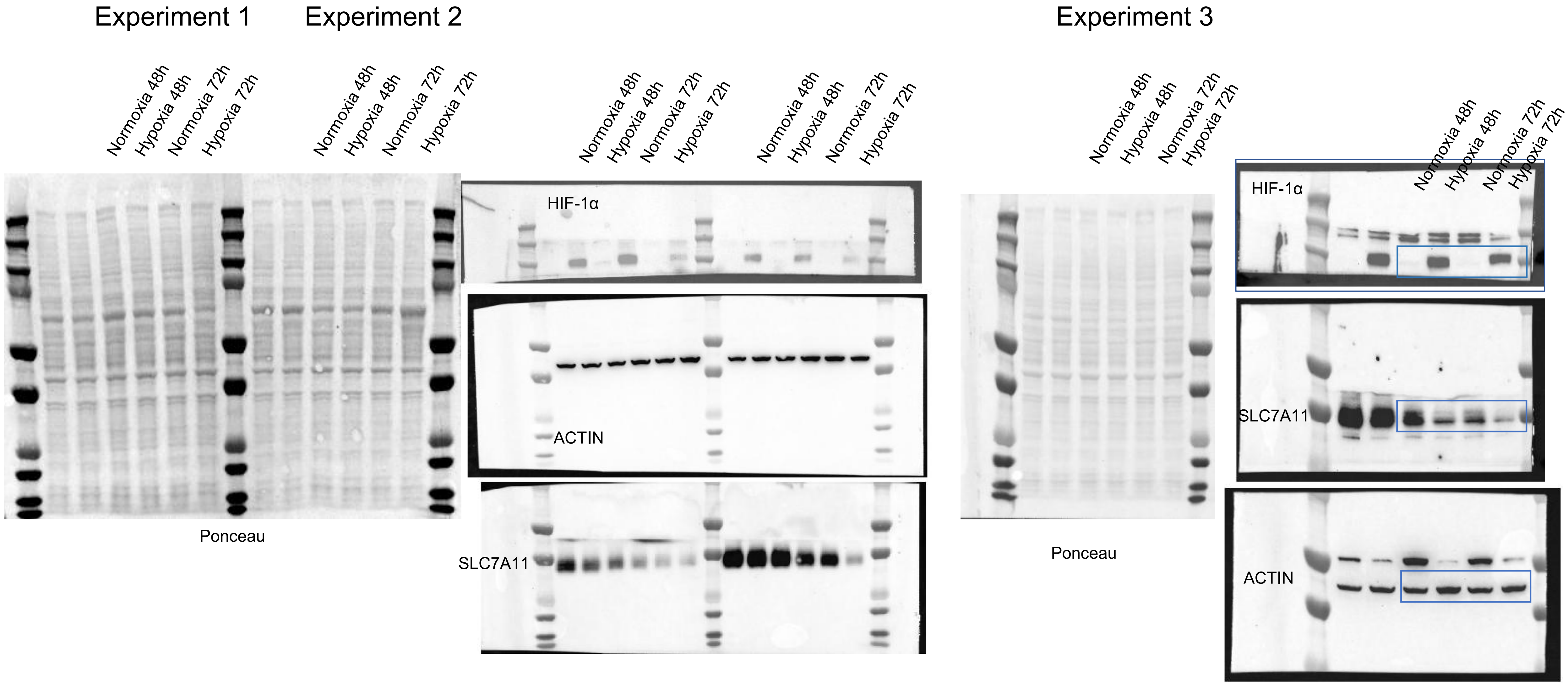

Experiment 1
Experiment 2
Experiment 3
Normoxia 48h
Hypoxia 48h
Normoxia 72h
Hypoxia 72h
HIF-1α
ACTIN
SLC7A11
Ponceau

Figure 5A

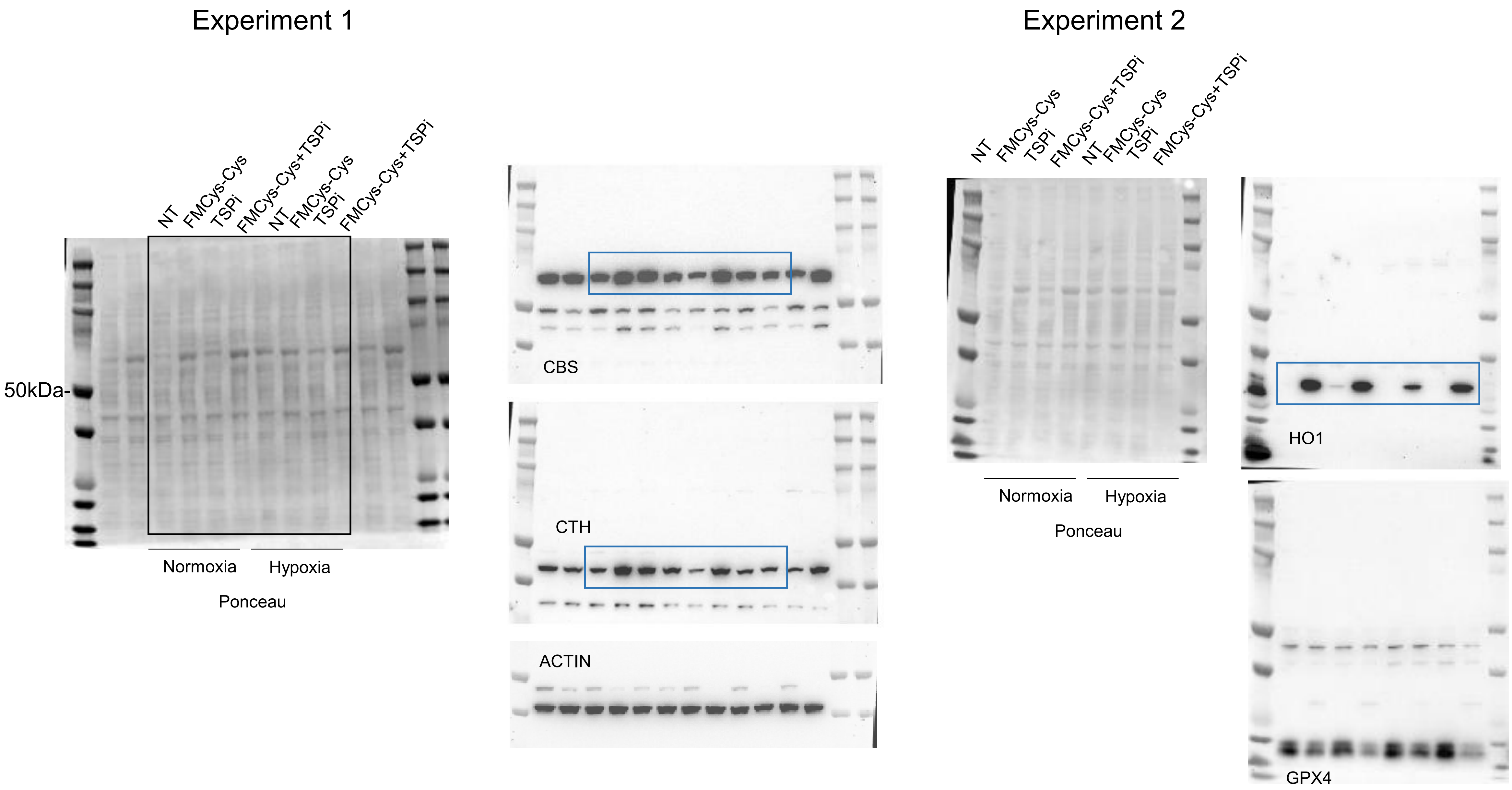

Experiment 1
NT
FMCys-Cys
TSPi
FMCys-Cys+TSPi
NT
FMCys-Cys
TSPi
FMCys-Cys+TSPi
50kDa-
Normoxia
Hypoxia
Ponceau
CBS
CTH
ACTIN
Experiment 2
NT
FMCys-Cys
TSPi
FMCys-Cys+TSPi
NT
FMCys-Cys
TSPi
FMCys-Cys+TSPi
Normoxia
Hypoxia
Ponceau
HO1
GPX4

Figure 5C

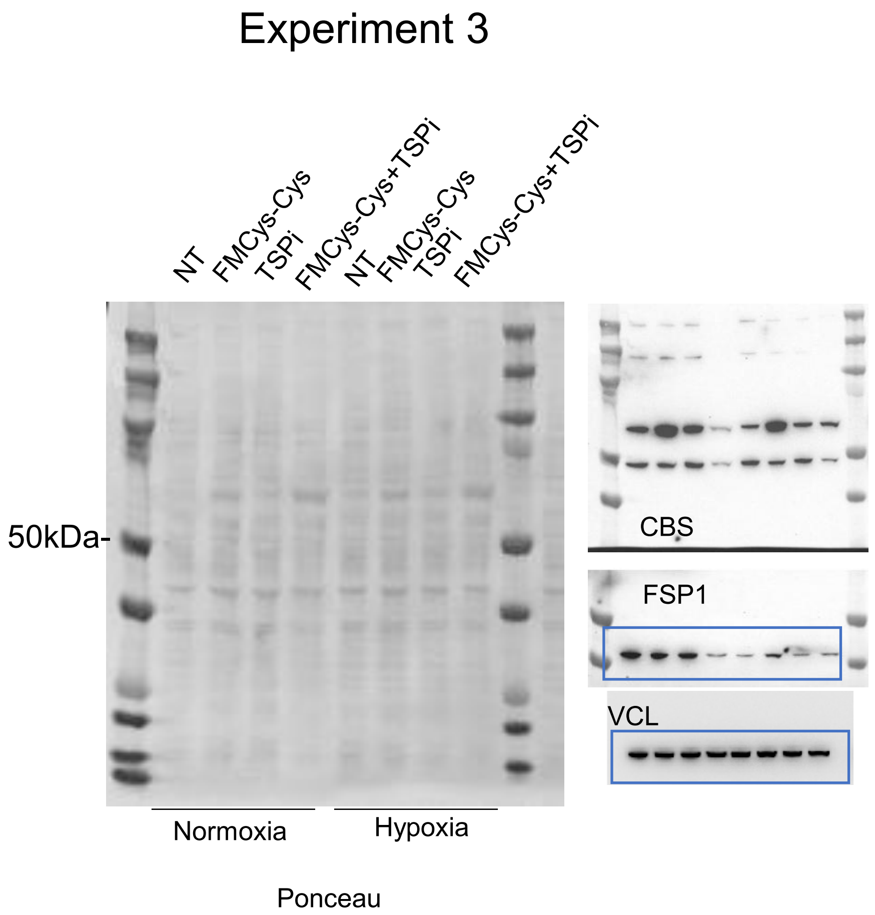
Experiment 3
NT
FMCys-Cys
TSPi
FMCys-Cys+TSPi
NT
FMCys-Cys
TSPi
FMCys-Cys+TSPi
50kDa-
CBS
FSP1
VCL
Normoxia
Hypoxia
Ponceau

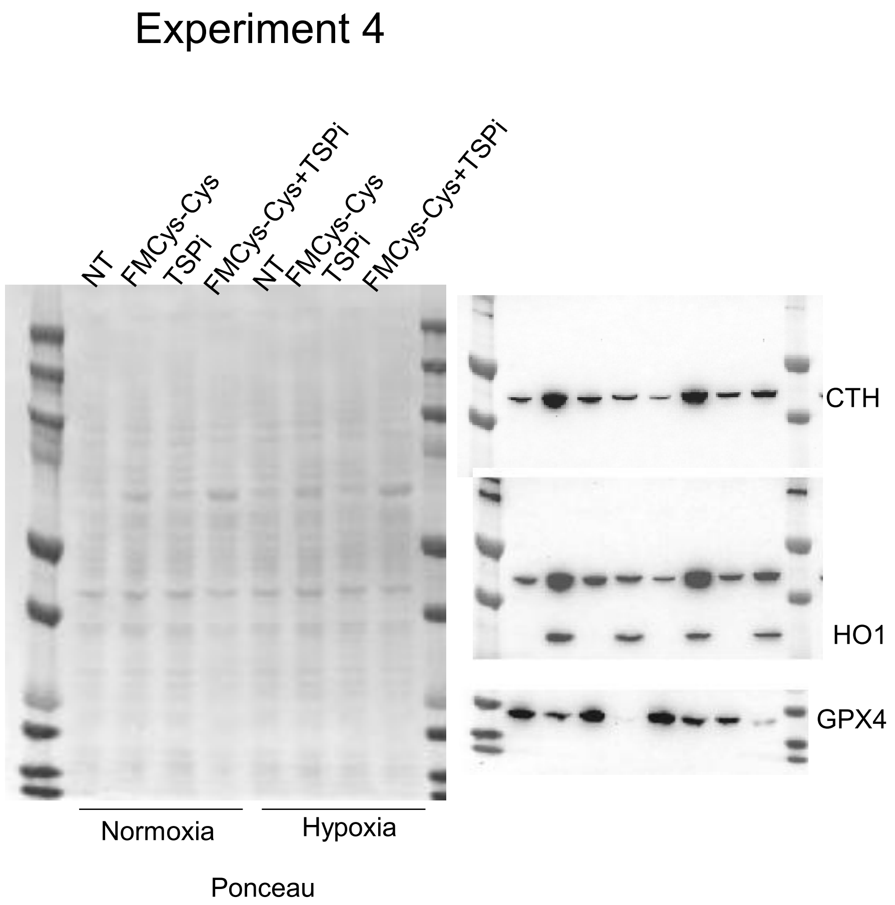
Experiment 4
NT
FMCys-Cys
TSPi
FMCys-Cys+TSPi
NT
FMCys-Cys
TSPi
FMCys-Cys+TSPi
CTH
HO1
GPX4
Normoxia
Hypoxia
Ponceau

Figure 5C

Experiment 5

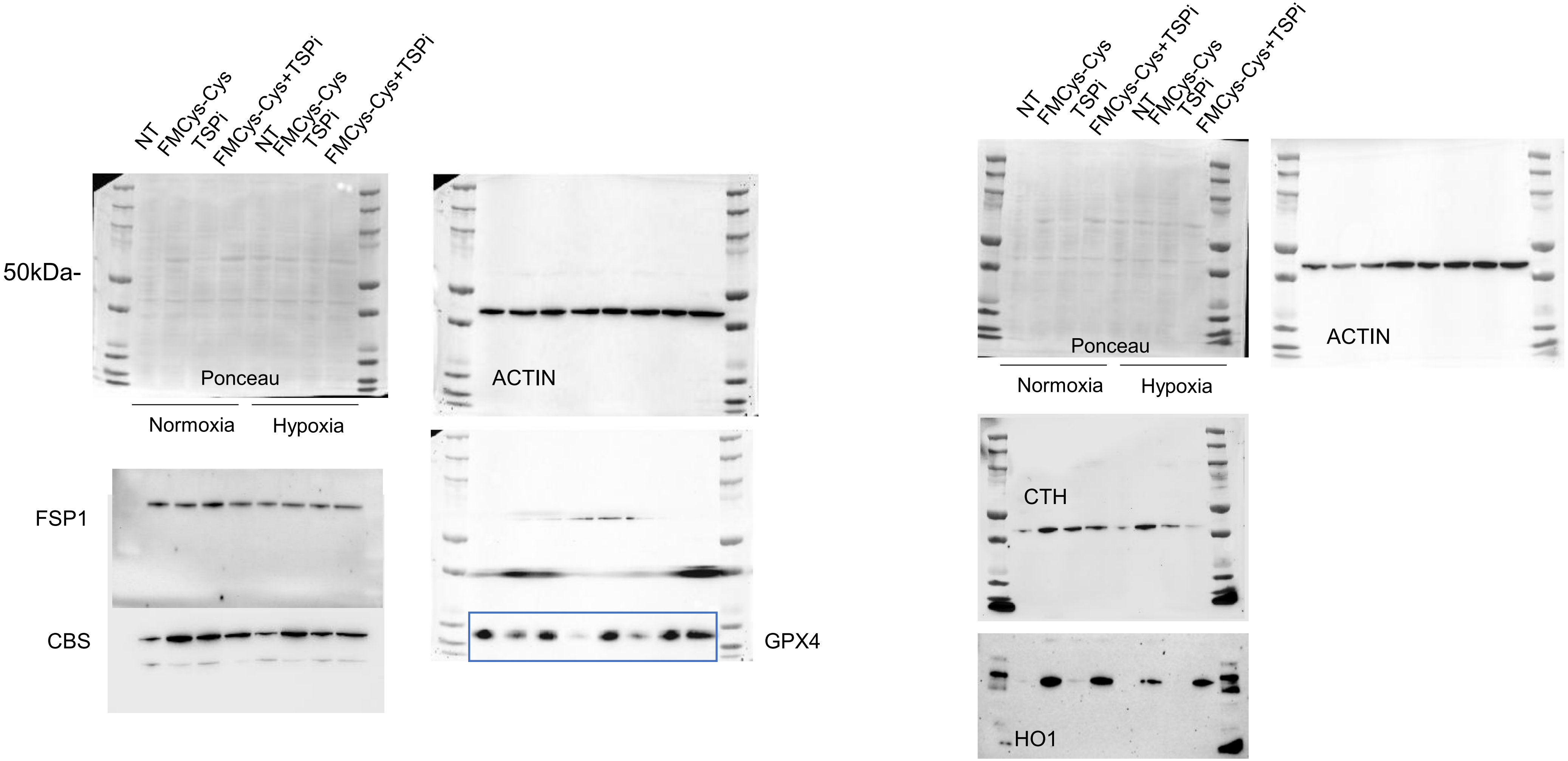

Figure 5C

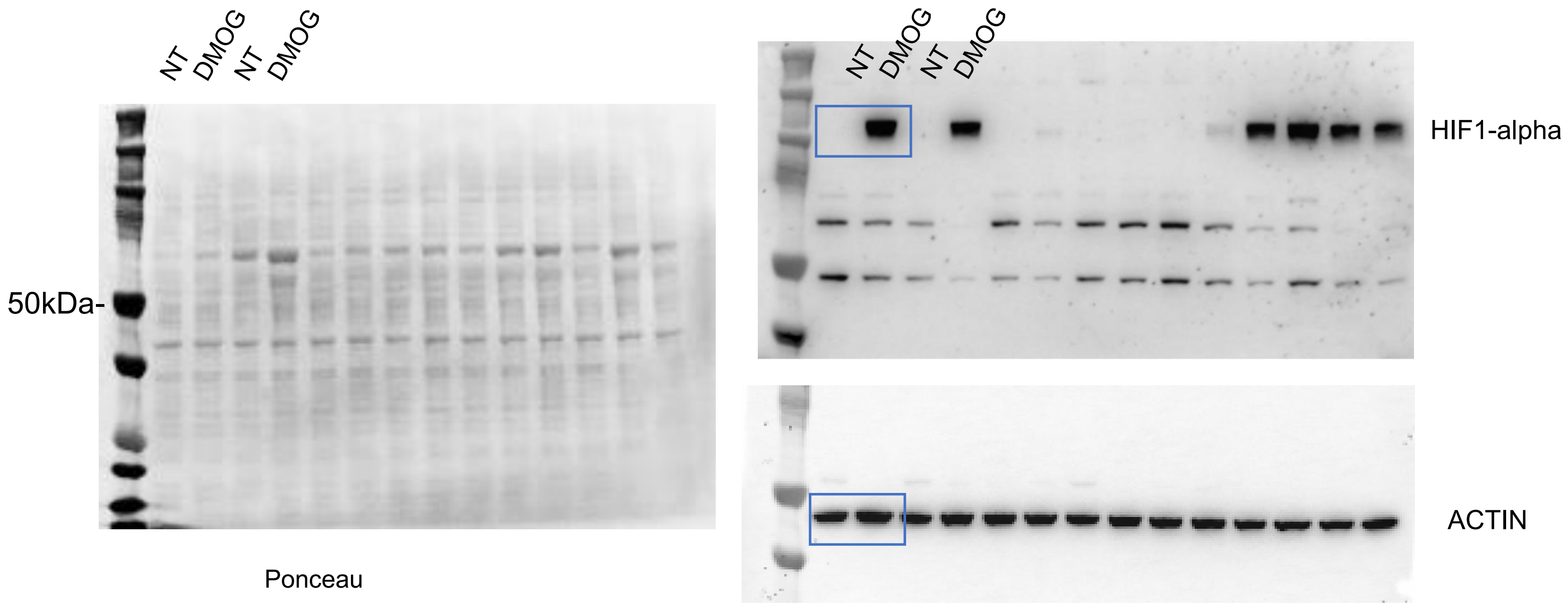

Sup. Figure 5A

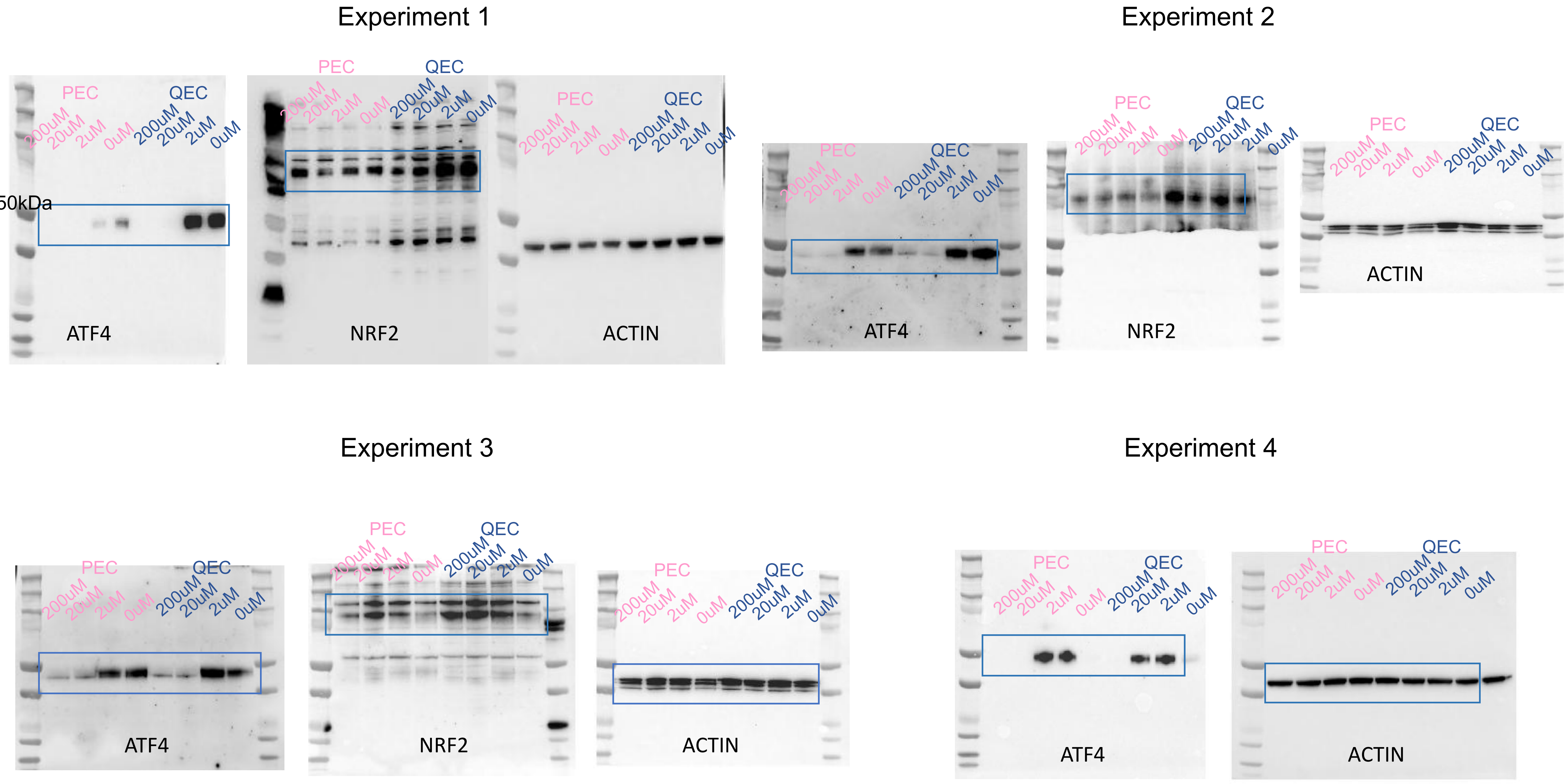

Figure 6A and 6D

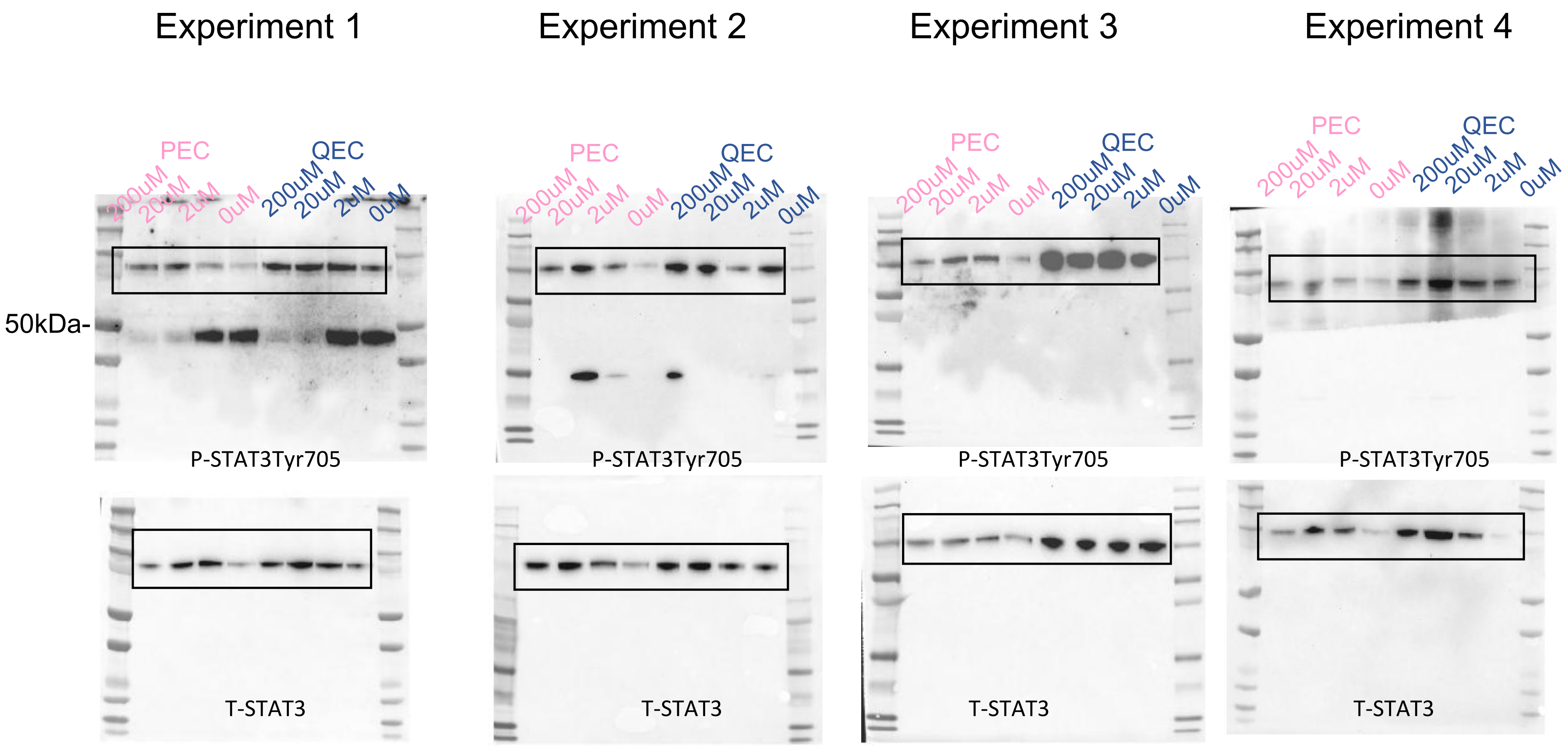
Experiment 1
Experiment 2
Experiment 3
Experiment 4
PEC
QEC
200uM
20uM
2uM
0uM
50kDa-
P-STAT3Tyr705
T-STAT3

Figure 6C

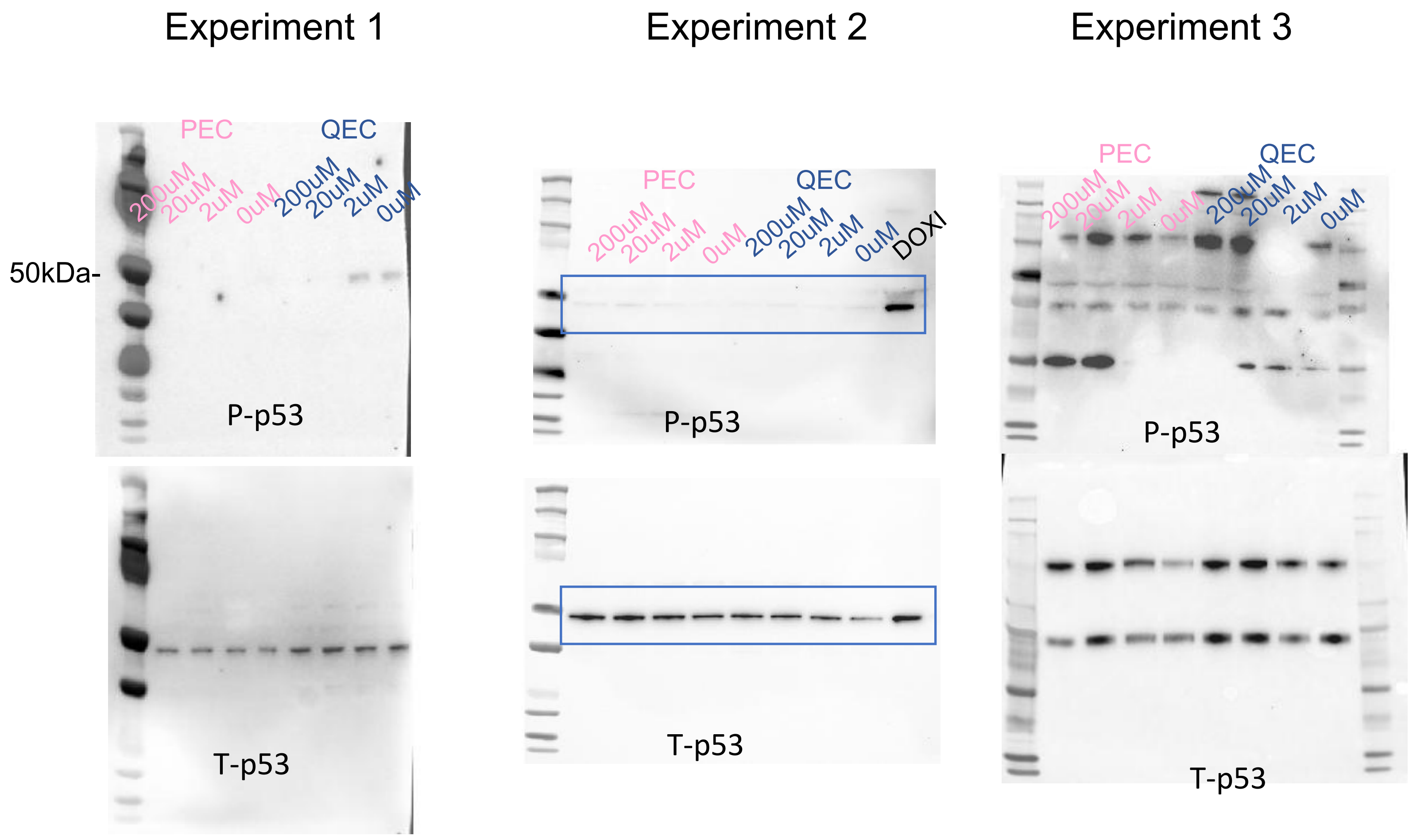
Experiment 1
Experiment 2
Experiment 3
PEC
QEC
200uM
20uM
2uM
0uM
200uM
20uM
2uM
0uM
50kDa-
P-p53
T-p53
PEC
QEC
200uM
20uM
2uM
0uM
200uM
20uM
2uM
0uM
DOXI
P-p53
T-p53
PEC
QEC
200uM
20uM
2uM
0uM
200uM
20uM
2uM
0uM
P-p53
T-p53

Figure 6B

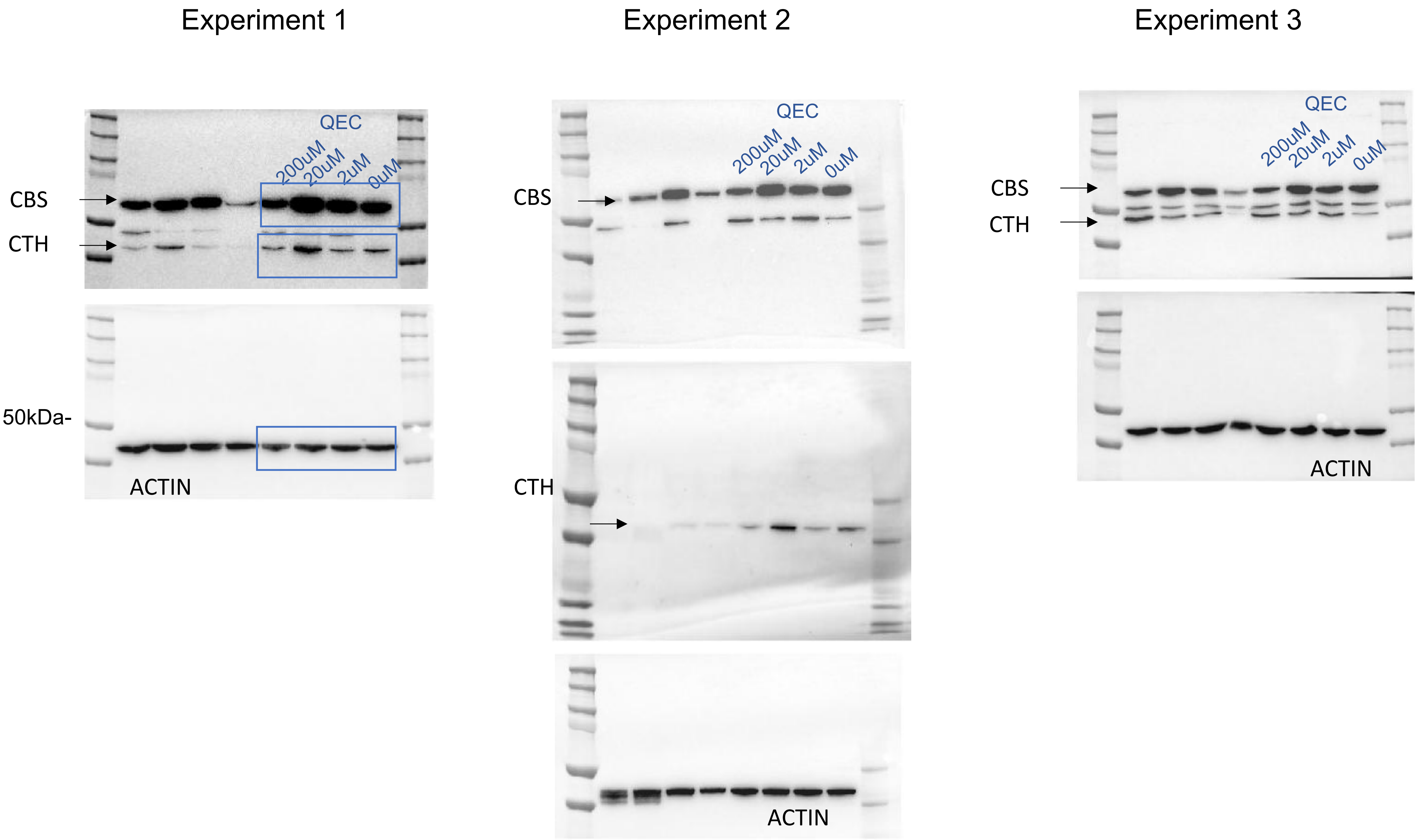
Experiment 1
Experiment 2
Experiment 3
QEC
200uM
20uM
2uM
0uM
CBS
CTH
50kDa-
ACTIN
QEC
200uM
20uM
2uM
0uM
CBS
CTH
ACTIN
QEC
200uM
20uM
2uM
0uM
CBS
CTH
ACTIN

Figure 6G
